# Synthesis of 7α-Methoxy-7-(4-phenyl-1*H*-1,2,3-triazol-1-yl)acetamino-3′-arylthio-cephalosporic Acid Derivatives from 7-Aminocephalosporic Acid

**DOI:** 10.3390/molecules28217338

**Published:** 2023-10-30

**Authors:** Wendy Y. Cun, Paul A. Keller, Stephen G. Pyne

**Affiliations:** School of Chemistry and Molecular Bioscience, Molecular Horizons Research Institute, University of Wollongong, Wollongong, NSW 2522, Australia; ywc538@uowmail.edu.au (W.Y.C.); keller@uow.edu.au (P.A.K.)

**Keywords:** cephamycin, cefotetan, antibiotics, triazole, thioallylation

## Abstract

The aim of this project was to develop a synthetic protocol for the preparation of a cephamycin scaffold that would readily allow the synthesis of its analogues with variations at the C-7 amino group and the C-3′ position. We also aimed to develop a method that avoided the use of toxic and potentially explosive diphenyldiazomethane. These aims were achieved via the synthesis of the novel α-bromo acetamide **18** which allowed functionalization at the α-bromo acetamide position by azide and then the introduction of a 4-phenyl-1*H*-1,2,3-triazol-1-yl moiety via a Cu(I)-catalysed azide–alkyne cycloaddition reaction with phenylacetylene. Palladium-catalyzed arylthioallylation reactions then allowed the introduction of 3′-arylthiol substituents. We also report for the first time the synthesis of the 4-methoxybenzyl ester of (6*R*,7*S*)-3-[(acetyloxy)methyl]-7-amino-7-methoxy-8-oxo-5-thia-1-azabicyclo[4.2.0]oct-2-ene-2-carboxylic acid and the use of diphenyl trichloroacetimidate, instead of diphenyldiazomethane, and 4-methoxybenzyl trichloroacetimidate to prepare related 4-methoxybenzyl esters. The chemistry described, and several of the synthetic intermediates reported here, are potentially valuable methods and scaffolds, respectively, for further development of β-lactam antibiotics.

## 1. Introduction

**Scheme 1:**
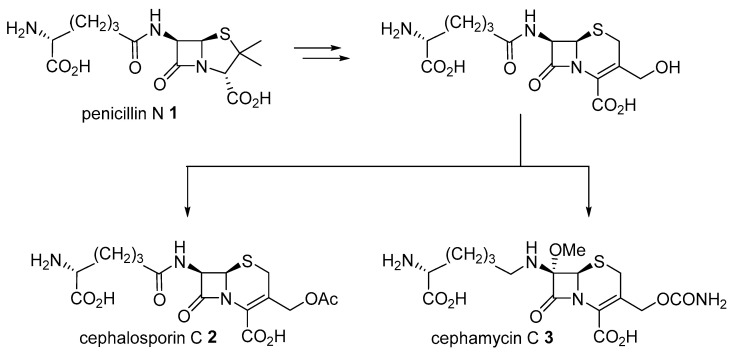
Simplified biosynthetic pathway for penicillin N **1**, cephalosporin C **2**, and cephamycin C **3**.

**Figure 1:**
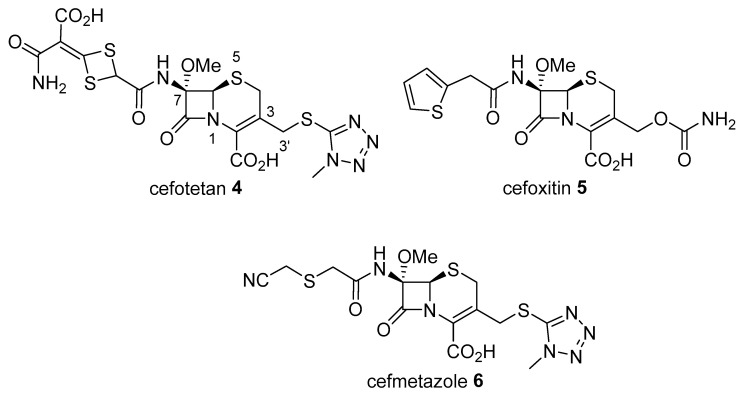
Chemical structures of the cephamycin antibiotics cefotetan **4**, cefoxitin **5**, and cefmetazole **6**.

The penicillins, cephalosprins, and cephamycins are important classes of clinically used β-lactam antibiotics [1]. Well-known representatives of each of these groups are penicillin N **1**, cephalosporium C **2,** and cephamycin C **3**, respectively. The latter two compounds are biosynthetically connected to penicillin N **1** by ring-expansion and, in the case of cephamycin C **3**, enzymatic introduction of a 7-α-methoxy substituent (Scheme 1) [1]. Examples of important cephamycin antibiotics are cefotetan **4**, cefoxitin **5**, and cefmetazole **6** (Figure 1) which are ascribed as second-generation cephalosporins with broad-spectrum in vitro antibacterial activity. Additionally, these compounds have anti-anaerobic activities making these valuable agents against intraabdominal infections [2]. Furthermore, their 7α-methoxy substituent decreases their vulnerabilities to β-lactamases [1], which potentially increases their antibacterial efficacies.

**Scheme 2:**
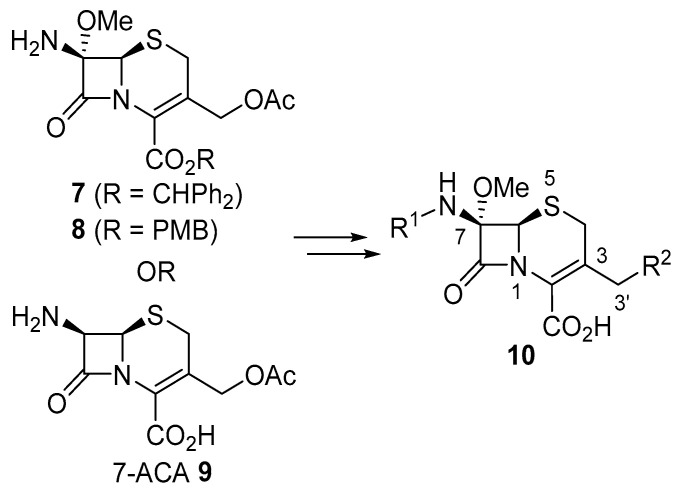
Proposed commercially available starting materials (compounds **7** or **9**) for the synthesis of cephamycin analogues **10** with variations at the C-7 amino group and the C-3′ position.

We became interested in the cephamycin compounds **4**–**6** when they were shown to induce an anti-sporulation effect against vegetative *Clostridioides difficile* cells [3]. Cefotetan **4** was the most potent inhibitor causing a 10,000-fold reduction in *C. difficile* sporulation activity at 15 nM. *C. difficile* is a gut-residing, spore-forming, anaerobic bacterium responsible for *C. difficile* disease (CDI). The spores are commonly associated with transmission, relapse, and recurrence of CDI and inhibiting this sporulation process could potentially prevent the recurrence of CDI [3]. In order to prepare analogues of compound **4** we required an advanced synthetic intermediate that would allow the synthesis of analogues (**10**) with variations at the C-7 amino group and the C-3′ position for structure–activity relationships studies and future antibacterial drug development (Scheme 2). While the commercially available 7α-methoxy cephalosporin intermediate **7** [diphenylmethyl (6*R*,7*S*)-3-[(acetyloxy)methyl]-7-amino-7-methoxy-8-oxo-5-thia-1-azabicyclo [4.2.0]oct-2-ene-2-carboxylic acid] would have been the ideal precursor it was prohibitively expensive (USD 14,739/10 g) [4]. We chose to start with 7-aminocephalosporic acid (7-ACA) **9**, which lacked the critical 7α-methoxy substituent; however, its cost was much more within our budget (US$ 50/100 g) (Scheme 2) [5].

In this paper we report our study on the synthesis of the diphenylmethyl ester **7** from 7-ACA **9** that avoids the use of toxic and potentially explosive diphenyldiazomethane [6] and the synthesis of the 4-methoxybenzyl (PMB) ester **8** from 7-ACA **9** and demonstrate here their application to the synthesis of derivatives of the type **10**. While compound **8** has been prepared previously, its synthesis is only described in the patent literature where only racemic **8** was prepared and very little characterization data for **8** or its precursors were described [7,8,9].

The main challenge in the synthesis of **7** and **8** from **9** was the introduction of the 7α-methoxy group. This has been achieved from the diphenylmethyl ester of 7-ACA **9** via treatment with HNO_2_, to give the 7-diazoderivative, followed by treatment with potentially explosive bromo azide to give a diastereomeric mixture of bromo azides. This mixture was then treated with methanol/AgBF_4_ to give the corresponding 7-azido-7-methoxy derivative and then hydrogenation gave **7** [10]. Lunn and Mason prepared **7**, via protection of the 7-amino group of 7-ACA **9** as a carbamate derivative and then esterification with diphenyldiazomethane [11]. The method of Koppel [12] was then employed via treatment of this diprotected compound using an excess amount of base (3.5 equiv. lithium methoxide) in tetrahydrofuran solvent and then *tert*-butyl hypochlorite at a low temperature (−80 °C) to generate a C-7 imine intermediate that was captured using the excess methoxide resulting in a C-7-aminocarbamate-C-7α-methoxy derivative. This then required carbamate deprotection via hydrogenolysis to give **7** as an unstable compound. An alternative procedure, that does not require potentially explosive reagents or intermediates or the use of strong base at low temperatures, was reported by Yanagisawa et al. [13,14]. This method involves oxidation of the Schiff base formed from the reaction of the diphenylmethyl ester of 7-ACA **9** with 3,5-di-*tert*-butyl-4-hydroxybenzaldehyde with lead dioxide and then treatment of the resulting C-7 imine with methanol. The imine of the resulting methanol adduct was then cleaved upon exposure to Girard-T reagent ((carboxymethyl)trimethylammonium chloride hydrazide) to give **7**. Yoshida later reported that lead dioxide could be replaced with 2,3-dichloro-5,6-dicyano-1,4-benzoquinone (DDQ) [15]. We chose to employ the method of Yoshida using DDQ as the oxidant.

## 2. Results and Discussion

**Scheme 3:**
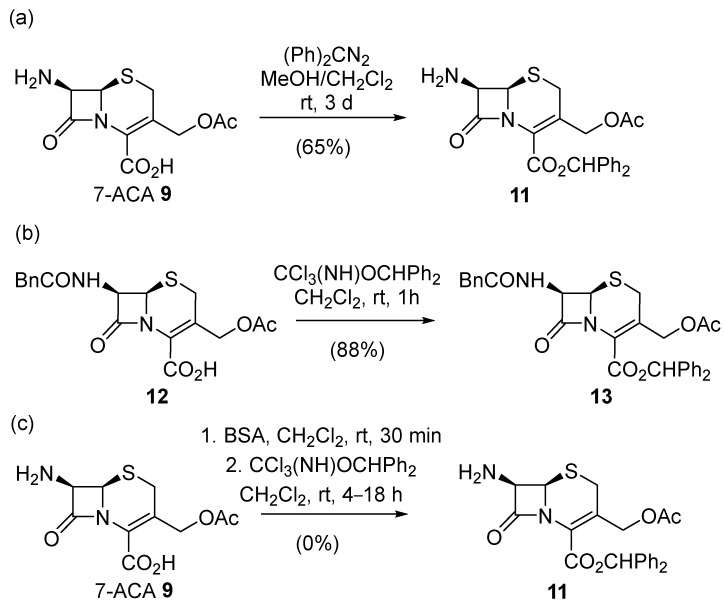
Synthesis of diphenylmethyl esters **11** and **13**. (**a**) Reported synthesis of diphenylmethyl ester **11** from 7-ACA **9** [16]; (**b**) Synthesis of a known diphenylmethyl ester **13** in a model study; (**c**) attempted synthesis of **11** in this study.

We initially investigated the synthesis of diphenylmethyl ester **11** from 7-ACA **9**. As indicated in (Scheme 3a) this has been prepared in 65% yield from the reaction of **9** with potentially hazardous diphenyldiazomethane (Scheme 3a) [16]. In a model study, we found that the known diphenylmethyl ester **13** [17] could be obtained in 88% yield from the reaction of acid **12** [18] and diphenyl trichloroacetimidate [19,20] in dichloromethane (CH_2_Cl_2_) solvent after 1 h at room temperature (rt) (Scheme 3b). However, our attempts to prepare **11** directly from 7-ACA **9** under similar reaction conditions were not successful due to the poor solubility of **9** in CH_2_Cl_2_. To prepare a more soluble substrate, a suspension of **9** in CH_2_Cl_2_ was treated first with *N*,*O*-bis(trimethylsilyl)acetamide (BSA) [21] to give a solution of the corresponding trimethylsilyl ester in situ followed by the addition of diphenyl trichloroacetimidate. However, only trace amounts of the desired product (**11**) could be detected using electron impact mass spectrometric (ESIMS) analysis (Scheme 3c).

**Scheme 4:**
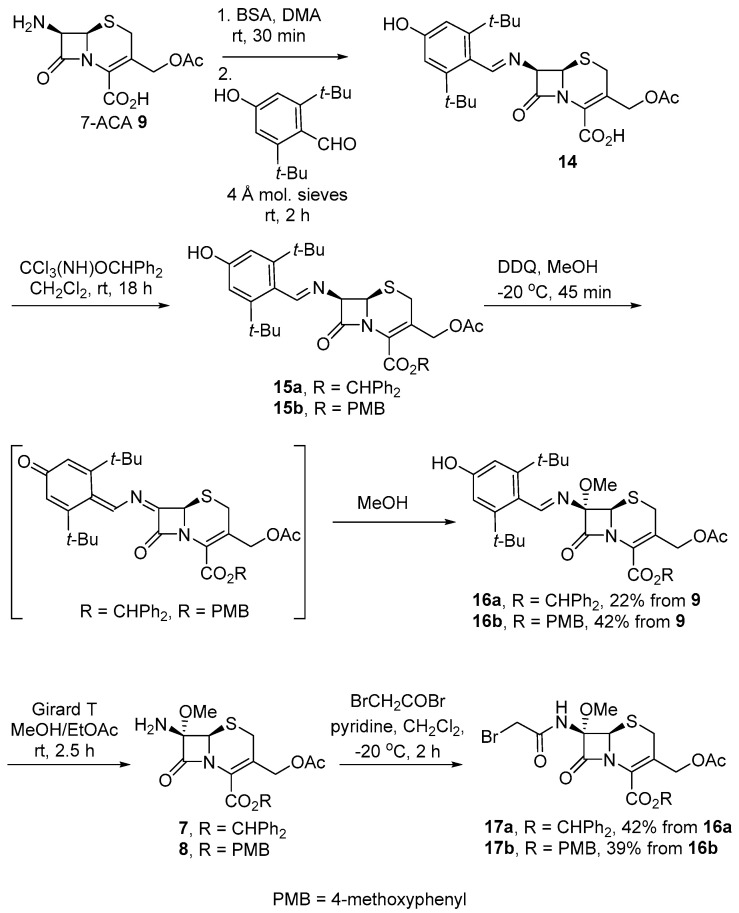
Synthesis of diphenylmethyl ester **7** and PMB ester **8** and their α–bromoacetamide derivatives **17a** and **17b**, respectively.

A more successful pathway was realized via the formation of the Schiff base **14** from the reaction of 7-ACA **9** first with BSA in dimethylacetamide (DMA) as a solvent and then treatment with 3,5-di-*tert*-butyl-4-hydroxybenzaldehyde (Scheme 4). This reaction mixture was then treated with diphenyl trichloroacetimidate to give the known diphenylmethyl ester **15a** [22]. Both compounds **14** and **15a** proved to be unstable to purification via column chromatography; however, the formation of imine **14** was evident in ^1^H NMR analysis of the crude reaction mixture (^1^H NMR (500 MHz,CD_3_SOCD_3_) in part, 8.44 (s, 1H, CH=NAr), 7.57 (s, 2H, ArCH), and 1.39 (s, 18H, 2 × C(CH_3_)_3_) ppm) and low resolution mass spectrometric (LRMS) analysis (ESI +ve) which showed an ion peak at *m/z* 489 ([M + H]^+^, 58%). While the formation of compound **15a** was evident from ^1^H NMR analysis of the crude reaction mixture (^1^H NMR (500 MHz, CD_3_OD) in part, 8.45 (s, 1H, CH=NAr) and 6.94 (s, 1H, CHPh_2_) ppm) it also contained unreacted 3,5-di-*tert*-butyl-4-hydroxybenzaldehyde and DMA. Oxidation of this mixture with DDQ in methanol solvent gave the known 7α-methoxylated imine derivative **16a** [13] in an optimized 22% overall yield for the three steps from **9**. Key to this optimized yield was performing the purification of **16a** using column chromatography below ambient temperature to prevent imine hydrolysis (see Experimental section for details). Treatment of **16a** with the Girard-T reagent gave a mixture of the known compound **7** [23] and the di-*tert-*butyl-4-hydroxybenzaldehyde imine of Girard’s reagent. Attempts to purify **7** were unsuccessful due to product instability; thus, the mixture was treated with bromoacetyl bromide and pyridine to give the more stable and novel α-bromo acetamide **17a** in 42% yield from **16a** (Scheme 4) after purification via column chromatography. In our studies of related acylation reactions, we found that low temperatures and short reaction times were essential to prevent isomerization of the C-2 double bond to the Δ-3 isomer [24]. This procedure was then applied to the synthesis of the PMB ester **8**, using commercially available 4-methoxyphenyl trichloroacetimidate rather than diphenyl trichloroacetimidate, and then its corresponding and novel α–bromoacetamide **17b** (Scheme 4). The ester **8** was able to be purified via column chromatography at ambient temperature; however, decomposition of this compound was observed during its characterization process, as evidenced by the color change of the sample (green to orange) and ^1^H NMR analysis. It was, therefore, used directly for the subsequent reaction without further purification. This latter synthetic protocol proved more convenient due to the commercial availability of 4-methoxyphenyl trichloroacetimidate and overall yields and gave a more stable intermediate—PMB ester **8**.

**Scheme 5:**
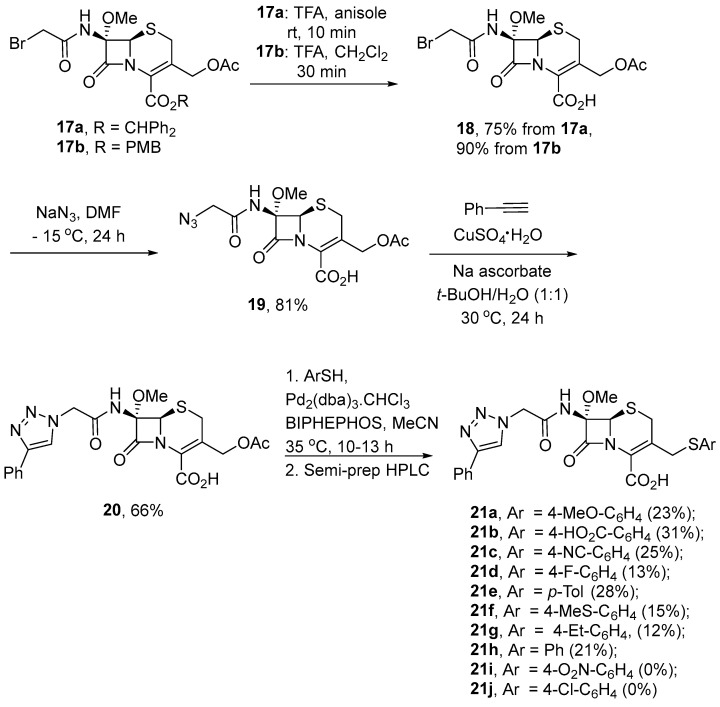
Functionalization of the α-acetamido and the C-3′ position of α–bromoacetamides **17a** and **17b**.

Compounds **17a** and **17b** are attractive intermediates for the synthesis of analogues related to the general structure **10** via the introduction of other substituents at the α-acetamide and the C-3′ positions. To explore this potential, we have converted **17a** and **17b** to the carboxylic acid **18** via treatment with trifluoroacetic acid (TFA) (Scheme 5). Treatment of this acid with sodium azide in dimethyl formamide (DMF) at −5 °C gave the azide **19** in 81% yield. We found that higher reaction temperatures led to mixtures of **19** and its double-bonded shifted isomer (Δ-3 isomer of **19**). The azide **19** was subjected to a Cu(I)-catalysed azide–alkyne cycloaddition (CuAAC) reaction [25] with phenylacetylene at 30 °C for 24 h, which provided the triazole **20** in 66% yield (Scheme 5). While the triazole **20** is a new compound, the corresponding 1*H*-tetrazole derivative has been reported in the patent literature [26].

We next focused on functionalization of the C-3′ position via displacement of the *O*-acetyl group with an arylthiol moiety using the palladium-catalyzed thioallylation method reported by Breinbauer et al. using 2 mol% bis(dibenzylideneacetone)palladium(0), 2 mol% 6,6′-[(3,3′-di-*tert*-butyl-5,5′-dimethoxy-1,1′-biphenyl-2,2′-diyl)bis(oxy)]bis(dibenzo[*d*,*f*][1,3,2]dioxaphosphepin) (BIPHEPHOS) as a ligand and acetonitrile as a solvent [27]. This method had been successfully applied by Breinbauer et al. to the cephalosporin antibiotic cefalotin, which, unlike **20**, bears a C-7 2-thienylacetamido substituent and lacks the 7α-methoxy group. Pd-catalyzed reactions of cefalotin with 4-methylthiophenol and 4-fluorothiophenol gave the corresponding C-3′ arylthiol derivatives in yields of 41% and 58%, respectively [27].

We initially studied the thioallylation reaction of **20** under similar reaction conditions, except using 1 mol% (2 mol% Pd) of tris(dibenzylideneacetone)dipalladium(0)-chloroform (Pd_2_(dba)_3_.CHCl_3_) as the palladium source (Table 1). However, after stirring the reaction under an argon atmosphere for 5 d at 35 °C, only unreacted **20** was evident from ^1^H NMR and MS analysis of the crude reaction mixture (Table 1, Entry 1). Similar results were obtained using 2 mol% triphenyl phosphite (P(OPh)_3_) or 1,1′-ferrocenediyl-bis(diphenylphosphine) (dppf) as the ligand (Table 1, Entries 2 and 3, respectively). We discovered, however, that performing the reaction using conditions of Entry 1 under sonication resulted in 70% conversion to the desired thiol derivative **21a** after 12 h (Table 1, Entry 4). Increasing the Pd and ligand loadings to 20 mol% and the equivalents of the thiol to 2.0 equiv., under sonication conditions, resulted in full conversion of **20** to **21a** (Table 1, Entry 5). After a standard work-up procedure, the crude product was purified using semi-preparative RP-HPLC to give **21a** in 23% yield and in 99% purity via analytical HPLC analysis (Scheme 4). Thiol derivatives **21b**–**21h** were then prepared under similar reaction conditions and purified using RP-HPLC with the yields shown in Scheme 4. In each case the analytical purities of these thiolated products were >99%, except for **21b** which had a purity of 98.7%. The 4-nitrophenylthio and the 4-chlorophenylthio derivatives **21i** and **21j**, respectively, could not be obtained using this synthetic protocol (Scheme 5).

Compounds **21a**–**21h** were screened for their antimicrobial activities against *Staphylococcus aureus* (ATCC 19603), *Escherichia coli* (ATCC 25922), *Klebsiella pneumoniae* (ATCC 25922), *Pseudomonas aeruginosa* (ATCC 27853), *Acinetobacter baumannii* (ATCC 19603), *Candida albicans* (ATCC 90028), and *Cryptococci neoformans* (ATCC 208821), at a concentration of 32 μg/mL. Colistin and vancomycin were used as positive controls for Gram-negative bacteria (colistin sulfate showed MIC_50_ values against *E. coli* (0.125 μg/mL), *K. pneumophila* (0.25 μg/mL), *A. baumannii* (0.25 μg/mL), and *P. aeruginosa* (0.25 μg/mL) and vancomycin.HCl showed a MIC_50_ value against *S. aureus* of 1 μg/mL). Fluconazole was used as the positive control in the anti-fungal assays (fluconazole showed MIC_50_ values against *C. albicans* (0.125 μg /mL) and *C. neoformans* (8 μg /mL)). However, none of these compounds showed any inhibitory activities.

## 3. Experimental

### 3.1. General Statement

Unless stated otherwise, all solvents and chemicals were laboratory- or reagent-grade and were purchased from commercial sources. Anhydrous solvents were obtained from a solvent dispenser under nitrogen and stored over 4 Å molecular sieves. All chemicals were used as received. All reactions were conducted under normal atmosphere with air or nitrogen. Cold reaction temperatures were obtained by using an ice bath (0 °C) or ice/salt bath (–20 °C). Heating of reactions was performed using a paraffin oil bath. Small quantities of liquid reagents were measured and added to reactions via syringe or autopipette. All filtrations were gravity filtrations through cotton wool or filter paper in a glass funnel. Solvent removal via concentration was performed on a rotary evaporator under reduced pressure. All synthesized compounds were dried under high vacuum (~1 mbar) before determination of chemical yields and spectroscopic characterization. All solvent mixtures are expressed in terms of volume ratio (i.e., *v*/*v*). Flash chromatography was performed using Chem Supply silica gel 60 230–400 mesh. Thin layer chromatography (TLC) was performed on Merck aluminum-backed SiO_2_ gel plates (F254 grade—0.20 mm thickness). A reversed-phase (RP) C18 (Synergi™ 4 µm Fusion-RP 80 Å (Elkridge, MD, USA), LC Column 150 × 4.6 mm) column was used with a MeCN/H_2_O (0:100–100:0) gradient mobile phase containing 0.01% TFA at a flow rate of 1.0 mL/min for the analysis. Compounds were detected using UV-vis at 279 or 254 nm, depending on their highest absorption. Visualization was achieved using UV light and cerium ammonium molybdate stain. All known compounds are marked with a reference after the compound title and all other compounds without a reference are novel. 

### 3.2. Characterization and Analysis

All novel compounds were subjected to full spectroscopic characterization and assignment based on 2-D NMR experiments. ^1^H NMR spectra were recorded on a Bruker Avance400 (400 MHz) and Bruker Avance500 (500 MHz) (Billerica, MA, USA). Chemical shifts are reported in ppm and were measured relative to the internal standard. Samples were dissolved in CDCl_3_ (with TMS as the internal standard—0.00 ppm) and CD_3_OD (solvent resonance as internal standard—3.31 ppm). The ^1^H NMR data are reported as follows: chemical shift, multiplicity (s = singlet, d = doublet, t = triplet, q = quartet, ABq = AB quartet, m = multiplet, br = broad), coupling constants (Hz), integration, and assignment. ^13^C NMR spectra were recorded on a Bruker Avance 400 (100 Hz) and Bruker Avance 500 (125 MHz) NMR spectrometer with complete ^1^H decoupling. Chemical shifts are reported in ppm and were measured relative to the internal standard. Samples were dissolved in CDCl_3_ (solvent resonance as internal standard—77.16 ppm) and CD_3_OD (solvent resonance as internal standard—49.0 ppm). ^1^H and ^13^C NMR signal assignments were confirmed via analysis of 2-D NMR experiments: gCOSY, gHSQC, and gHMBC. The abbreviations section defines all NMR experiment acronyms. All NMR spectra were processed, analyzed, and prepared with MestReNova (version 12.0) NMR software. Low resolution mass spectra (LRMS) were obtained via electrospray ionization (ESI) on a Shimadzu LC-2010 mass spectrometer (Kyoto, Japan). LRMS data were recorded as the ion mass/charge ratio (*m*/*z*) with the corresponding relative abundance as a percentage. High-resolution mass spectrometry (HRMS) was performed on a Waters Quadrupole Time of Flight (QTOF) Xevo spectrometer via ESI and with Leucine-Enkephalin as an internal standard. All mass spectrometry samples were dissolved in high-performance liquid chromatography (HPLC)-grade MeOH. Rotation values (⍺) are expressed in units of “deg cm^3^ g^−1^ dm^−1^” with concentration (*c*) expressed in units of “g/100 mL”. Solid-state infrared spectroscopy was performed on a Bruker Vertex 70 FTIR spectrometer. IR peaks are reported as the wavenumber (ν_max_ in cm^–1^) of the maximum absorption, and the intensities were expressed as s = strong, m = medium, or w = weak. The purity of all tested compounds was determined using analytical HPLC—Waters 1525 binary HPLC pump with a Waters 2487 dual-absorbance detector (column, Synergi Fusion-RP 80Å, 4.6 × 150 mm, 4 µm; flow rate, 1.0/min; method, 0–100 MeCN. UV wavelength, 254 or 279 nm; temperature, 30 °C; injection volume, 10 μL). Compounds **21a**–**h** were purified via reversed-phase (RP) HPLC (column, SynergiTM Fusion-RP 80 Å, LC Column 250 × 10 mm, 4 μm; flow rate, 3.8/min; method, 3:5–7:10 MeCN, 15 min. UV wavelength, 254 or 279 nm; temperature, RT; injection volume, 200 μL).

(7*S*)-3-(Acetoxymethyl)-7-benzamido-7-methoxy-8-oxo-5-thia-1-azabicyclo[4.2.0]-oct-2-ene-2-carboxylic acid **12**

To a suspension of 7-ACA **9** (307.4, 1.13 mmol) in anhydrous DMA (5 mL) under N_2_ at rt, BSA (469.3 μL, 1.92 mmol, 1.7 equiv.) was added and stirred for 30 min until the mixture turned clear. The reaction mixture was then cooled to −20 °C (NaCl/ice), and phenylacetyl chloride (179.2 μL, 1.36 mmol, 1.2 equiv.) was added dropwise. The resulting solution was stirred at −20 °C for 2 h, at which point the reaction was shown to be complete via TLC analysis (TLC (MeOH/CH_2_Cl_2_—2:3): R*_f_* = 0.63). The reaction mixture was poured into iced water (20 mL) and extracted using EtOAc (3 × 20 mL). The combined EtOAc layer was washed with water (3 × 20 mL), brine (20 mL), dried over anhydrous MgSO_4_, filtered, and concentrated in vacuo to give a pale-yellow residue. The obtained crude product was then dissolved in a minimum amount of EtOAc, and hexanes were added dropwise to this vigorously stirred solution until precipitation started to occur. The mixture was stirred at rt overnight. The solvent was removed using a syringe and the precipitate was washed with hexanes (5 × 10 mL), then dried in vacuo to afford the titled compound as an off-white powder (258.1 mg, 0.661 mmol, 58%). ^1^H NMR (500 MHz, CD_3_COCD_3_) δ 7.95 (d, *J* = 8.7 Hz, 1H, NH), 7.26–7.07 (m, 5H, ArCH), 5.71 (dd, *J* = 8.7, 4.8 Hz, 1H, H7), 5.00 (d, *J* = 4.9 Hz, 1H, H6), 4.83 (AB*q*, *J*_A,B_ = 13.1 Hz, 2H, O-CH_2_, acetate), 3.58–3.47 (m, 3H, CH_2_Ph and H4_A_), 3.39 (d, *J* = 18.3 Hz, 1H, H4_B_), 1.90 (s, 3H, CH_3_); the ^1^H NMR spectroscopic data were in agreement with those previously reported [18]. MS (ESI +ve) *m/z* 413 ([M + Na]^+^, 100%), 429 ([M + K]^+^, 74%), 803 ([2M + Na]^+^, 21%); (ESI–ve) *m/z* 389 ([M − H]^−^, 65%), 779 ([2M − H]^−^, 100%).

Benzhydryl 2,2,2-trichloroacetimidate [19,20]

To a solution of diphenylmethanol (317.3 mg, 1.72 mmol) in anhydrous CH_2_Cl_2_ (2 mL) DBU (25.8 μL, 0.172 mmol, 0.1 equiv.) and CCl_3_CN (1.73 mL, 17.2 mmol, 10 equiv.) were added at rt under an Ar atmosphere. The reaction mixture was stirred at 40 °C overnight, at which point the reaction was shown to be complete via TLC analysis (TLC (3% Et_3_N in toluene): R*_f_* = 0.71). Reaction mixture was concentrated in vacuo and purified via flash chromatography over SiO_2_ (3% Et_3_N in hexane/EtOAc—80:1) to give the titled compound as a white solid (375.8 mg, 1.14 mmol, 66%). ^1^H NMR (500 MHz, CDCl_3_) δ 8.41 (s, 1H, NH), 7.50–7.23 (m, 10H, ArCH), 6.94 (s, 1H, CHPh_2_); the ^1^H NMR spectroscopic data agreed with those previously reported [19,20].

Benzhydryl (6*R*,7*R*)-3-(acetoxymethyl)-8-oxo-7-(2-phenylacetamido)-5-thia-1-azabicyclo[4.2.0]oct-2-ene-2-carboxylate **13** [17]

To a suspension of **12** (99.1 mg, 0.254 mmol) in anhydrous CH_2_Cl_2_ (2 mL) benzhydryl 2,2,2-trichloroacetimidate (108.4 mg, 0.330 mmol, 1.3 equiv.) was added at rt under an Ar atmosphere. The reaction mixture was stirred for 1 h until it turned into a clear pale-yellow solution. The completion of the reaction was indicated via TLC analysis (TLC (MeOH/CH_2_Cl_2_—1:9): R*_f_* = 0.86). The obtained crude product was then dissolved in a minimum amount of EtOAc, and hexane was added dropwise to this vigorously stirred solution until precipitation started to occur. The mixture was stirred at rt overnight. The solvent was removed using a syringe and the precipitate was washed with hexanes (5 × 10 mL), then dried in vacuo to afford the titled compound as an off-white powder (123.9 mg, 0.222 mmol, 88%). ^1^H NMR (500 MHz, CDCl_3_) δ 7.44–7.25 (m, 15H, Ar-CH), 6.93 (s, 1H, CHPh_2_), 6.02 (d, *J* = 9.1 Hz, 1H, NH), 5.86 (dd, *J* = 9.0, 4.9 Hz, H7), 4.88 (ABq, *J*_A,B_ = 13.6 Hz, 2H, O-CH_2_, acetate), 4.95 (d, *J* = 4.9 Hz, H6), 3.65 (ABq, *J*_A,B_ = 16.2 Hz, 2H, CH_2_Ph), 3.43 (ABq, *J*_A,B_ = 18.6 Hz, 2H, H4), 2.01 (s, 3H, CH_3_); the ^1^H NMR spectroscopic data agreed with those previously reported [17]; MS (ESI + ve) *m/z* 630 ([M + Na]^+^, 100%); (ESI –ve) *m/z* 686 ([M − H]^−^, 62%).

For NMR assignments of compounds **7**, **8,** and **14**–**19**, the following numbering system has been used.


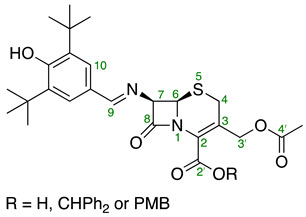

(6*R*,*7R*)-3-(Acetoxymethyl)-7-(((*E*)-3,5-di-*tert*-butyl-4-hydroxybenzylidene)-amino)-8-oxo-5-thia-1-azabicyclo[4.2.0]oct-2-ene-2-carboxylic acid **14**

To a suspension of 7-ACA **9** (134.0 mg, 0.492 mmol) in DMA (1 mL) BSA (204.6 µL, 1.29 mmol, 1.7 equiv.) was added, and the reaction mixture was stirred at rt for 30 min until the white suspension turned into an orange solution. Powdered molecular sieves (30 mg, 4 Å) and 3,5-di-*tert*-butyl-4-hydroxybenzaldehyde (121.1 mg, 0.517 mmol, 1.05 equiv.) were then added to the reaction mixture and stirred at rt for 2 h until the pale-orange color of the reaction turned into bright yellow. The completion of the reaction was indicated via TLC analysis (TLC (MeOH/CH_2_Cl_2_—2:3): R*_f_* = 0.76). A portion of the reaction mixture was diluted with MeOH (10 mL) and filtered. The obtained filtrate was concentrated for NMR analysis, which indicated a mixture of the titled compound and 3,5-di-*tert*-butyl-4-hydroxybenzaldehyde. The reaction mixture was concentrated under a stream of N_2_ overnight to give crude product **14** as a yellow gum. ^1^H NMR (500 MHz, CD_3_SOCD_3_) δ 8.44 (s, 1H, H9), 7.57 (s, 2H, H10), 5.53 (d, *J* = 4.9, 1H, H7), 5.28 (d, *J* = 5.0 Hz, 1H, H6), 4.84 (ABq, *J*_A,B_ = 12.7 Hz, 2H, H3′), 3.53 (ABq, *J*_A,B_ = 17.3 Hz, 2H, H4), 2.04 (s, 3H, C4′-CH_3_), 1.39 (s, 18H, 2 × C-(CH_3_)_3_); (ESI +ve) *m/z* 489 ([M + H]^+^, 58%).

Benzhydryl (6*R*,7*R*)-3-(acetoxymethyl)-7-(((*E*)-3,5-di-*tert*-butyl-4-hydroxy-benzylidene)amino)-8-oxo-5-thia-1-azabicyclo[4.2.0]oct-2-ene-2-carboxylate **15a**

To a suspension of crude product **14** (0.735 mmol, prepared from 200.1 mg of 7-ACA) in CH_2_Cl_2_ (2 mL) under an Ar atmosphere at rt benzhydryl 2,2,2-trichloroacetimidate (314.0 mg, 0.956 mmol, 1.3 equiv.) was added. The color of the reaction turned from bright yellow to brown, and the completion of the reaction was indicated using TLC analysis (TLC (MeOH/CH_2_Cl_2_—1:9): R*_f_* = 0.74). The reaction mixture was concentrated in vacuo and the obtained brown residue was suspended in MeOH (30 mL) and filtered. The filtrate was concentrated for NMR analysis, which indicated a mixture of the titled compound, DMA and 3,5-di-*tert*-butyl-4-hydroxybenzaldehyde. Further purification using flash chromatography resulted in decomposition. ^1^H NMR (500 MHz, CD_3_OD) δ 8.45 (s, 1H, H9), 7.65 (s, 2H, H10), 7.46–7.22 (m, 10H, Ar-CH), 6.94 (s, 1H, CHPh_2_), 5.46 (d, *J* = 5.1 Hz, 1H, H7), 5.24 (d, *J* = 5.1 Hz, 1H, H6), 4.98 (d, *J* = 13.1 Hz, 1H, H3′_A_), 4.71 (d, J = 13.1 Hz, 1H, H3ʹ_B_), 3.56 (ABq, *J*_A,B_ = 18.5 Hz, 2H, H4), 1.98 (s, 3H, C4′-CH_3_), 1.46 (s, 18H, 2 × C-(CH_3_)_3_); the ^1^H NMR spectroscopic data agreed with those previously reported [22]; (ESI +ve) *m/z* 655 ([M + H]^+^, 51%), 677 ([M + Na]^+^, 100%); (ESI −ve) *m/z* 653 ([M−H]^−^, 100%).

Benzhydryl (6*R*,7*S*)-3-(acetoxymethyl)-7-(((*E*)-3,5-di-*tert*-butyl-4-hydroxy-benzylidene)amino)-7-methoxy-8-oxo-5-thia-1-azabicyclo[4.2.0]oct-2-ene-2-carboxylate **16a**

To a suspension of crude product **15a** from the previous steps (0.163 mmol, prepared from 44.5 mg of 7-ACA **9**) in MeOH (1.5 mL) at −20 °C DDQ (37.0 mg, 0.163 mmol, 1.0 equiv.) was added, and the reaction mixture was stirred at −20 °C for 45 min. The completion of the reaction was indicated using MS analysis. EtOAc (1.5 mL) was then added to the reaction and the resulting mixture was stirred for another 30 min. The dark red reaction mixture was concentrated in vacuo and purified via flash chromatography over SiO_2_ (EtOAc/hexane—1:10–3:10) to give the title compound as a hydroscopic, yellow foam (24.6 mg, 0.0359 mmol, 22% from 7-ACA **9**).

Alternative purification method for scale-up reactions (≥200 mg of 7-ACA **9**).

A series of solvents in a EtOAc/hexane system were prepared (using 3.6 mmol crude product as an example: 100 mL—1:19, 200 mL—1:9, 100 mL—3:17, 100 mL—1:4, 100 mL—11:39, 100 mL—6:19, 100 mL—13:37, 100 mL—7:18, and 200 mL—3:7) and chilled at −20 °C overnight. On the following day, an appropriately sized column was packed with a SiO_2_ slurry in hexane and chilled at −20 °C until ready to use. Once the crude product was loaded onto the column, the pre-chilled solvents was kept cool on an ice bath and the flow rate was accelerated by using a stream of compressed air to ensure the entire purification process was kept within 30 min. ^1^H NMR (500 MHz, CDCl_3_) δ 8.54 (s, 1H, H9), 7.69 (s, 2H, H10), 7.51–7.29 (m, 10H, Ar-CH), 6.97 (s, 1H, CHPh_2_), 5.64 (s, 1H, OH), 5.08 (s, 1H, H6), 4.85 (ABq, *J*_A,B_ = 13.4 Hz, 2H, H3′), 3.57 (s, 3H, O-CH_3_), 3.39 (ABq, *J*_A,B_ = 18.2 Hz, 2H, H4), 2.00 (s, 3H, C4′-CH_3_), 1.46 (s, 18H, 2 × C-(CH_3_)_3_); the ^1^H NMR spectroscopic data agreed with those previously reported [13]; (ESI +ve) *m/z* 685 ([M + H]^+^, 100%), 707 ([M + Na]^+^, 79%); (ESI−ve) *m/z* 683 ([M − H]^−^, 81%).

Benzhydryl (6*R*,7*S*)-3-(acetoxymethyl)-7-amino-7-methoxy-8-oxo-5-thia-azabicyclo[4.2.0]oct-2-ene- 2-carboxylate **7**

To a solution of **16a** (56.4 mg, 0.0824 mmol) in EtOAc (0.5 mL) a solution of Girard-T reagent (27.6 mg, 0.165 mmol, 2.0 equiv.) was added in MeOH (0.6 mL) at rt, and the resulting solution was stirred for 2.5 h until the completion of the reaction was indicated by MS analysis. Upon completion, the reaction mixture was diluted with EtOAc (15 mL) and poured into water (20 mL), which was extracted with additional EtOAc (15 mL × 2). The combined EtOAc layer was washed with brine (20 mL), dried over anhydrous Na_2_SO_4_, filtered, and concentrated in vacuo to give the crude product as a dark green gum. Further purification of the crude product resulted in decomposition and it was, therefore, used directly for the subsequent reaction. ^1^H NMR (500 MHz, CDCl^3^) δ 7.41–7.27 (m, 10H, ArCH), 6.94 (s, 1H, CHPh_2_), 5.03 (d, *J* = 13.7 Hz, 1H, H3′_A_), 4.85 (s, 1H, H6), 4.83 (d, *J* = 13.7 Hz, 1H, H3′_B_), 3.52 (s, 3H, O-CH_3_), 3.44 (d, *J* = 17.2 Hz, 1H, H4_A_), 3.31 (d, *J* = 17.2 Hz, 1H, H4_B_), 2.02 (s, 3H, C4′-CH_3_); (ESI +ve) *m/z* 469 ([M + H]^+^, 11%), 491 ([M + Na]^+^, 100%), and 501 ([M + MeOH + H]^+^, 9%). The sample of **7** also contained (*E*)-2-(2-(3,5-di-*tert*-butyl-4-hydroxybenzylidene)hydrazineyl)-*N*,*N*,*N*-trimethyl-2-oxoethan-1-aminium chloride in a 1:1 ratio from ^1^H NMR analysis: ^1^H NMR (500 MHz, CDCl_3_) δ 8.38 (s, 1H, H3), 7.57 (s, 2H, H4), 4.65 (s, 2H, H2), 3.42 (s, 9H, H1), 1.43 (s, 18H).

Benzhydryl (6*R*,7*S*)-3-(acetoxymethyl)-7-(2-bromoacetamido)-7-methoxy-8-oxo-5-thia-1-azabicyclo[4.2.0]oct-2-ene-2-carboxylate **17a**

To a solution of crude product **7** (0.0813 mmol, prepared from 55.6 mg of **16a** from the previous reaction in anhydrous CH_2_Cl_2_ (1 mL) under N_2_ at −20 °C (NaCl/ice) pyridine (20 μL, 0.248 mmol, 3.1 equiv.) was added and the resulting solution was stirred for 2 min. Bromoacetyl bromide (19 μL, 0.216 mmol, 2.7 equiv.) was then added dropwise to the solution. The reaction mixture was stirred at −20 °C for 2 h, at which point the reaction was shown to be complete via TLC analysis (TLC (EtOAc/hexane—2:3): R*_f_* = 0.39). The reaction mixture was poured into EtOAc (5 mL); washed sequentially with HCl (1.0 M—5 × 5 mL), saturated NaHCO_3_ (5 × 5 mL), and brine (10 mL); dried over anhydrous Na_2_SO_4_; filtered; and concentrated in vacuo to give a brown residue. The crude product was purified via flash chromatography over SiO_2_ (EtOAc/hexane—1:19–9:11) to give the titled compound as a pale-yellow foam (20.3 mg, 0.0344 mmol, 42% from **16a**). ${\left[\alpha \right]}_{D}^{22}$ +109.45 (*c* 0.22, CH_2_Cl_2_); ^1^H NMR (400 MHz, CDCl_3_) δ 7.51–7.23 (m, 10H, ArCH), 6.95 (s, 1H, CHPh_2_), 5.09 (s, 1H, H6), 4.95 (ABq, *J*_A,B_ = 13.8 Hz, 2H, H3ʹ), 3.92 (ABq, *J*_A,B_ = 13.6 Hz, 2H, Br-CH_2_), 3.58 (s, 3H, O-CH_3_), 3.39 (ABq, *J*_A,B_ = 18.2 Hz, 2H, H4), 2.02 (s, 3H, C4′-CH_3_); NH signal was not observed; ^13^C NMR (101 MHz, CDCl3) δ 170.4 (C4′), 166.6 (C=O, amide), 160.2 (C2ʹ), 160.1 (C8), 139.3 (Cq, benzhydryl), 139.1 (Cq, benzhydryl), 132.0 (C3), 128.57 (ArCH), 128.55 (ArCH), 128.5 (ArCH), 128.2 (ArCH), 128.2 (ArCH), 127.5 (ArCH), 127.04 (ArCH), 127.01(ArCH), 125.9 (C2), 95.8 (C7), 79.7(CH-Ph_2_), 64.3 (C6), 62.4 (C3′), 54.0 (O-CH3), 27.9 (Br-CH_2_), 27.0 (C4), 20.64 (CH_3_); IR (cm^−1^) $\bar{\nu }$_max_ 3278 (w), 2960 (w), 1775 (s, β-lactam C=O), 1735 (s, C=O, acetate), 1608 (m), 1517 (s), 1380 (m), 1237 (s, C-O stretching, acetate), 1129 (m), 1090 (m), 1032 (m), 749 (w), 700 (m); MS (ESI + ve) *m/z* 611 ([^79^BrM + Na]^+^, 90%), 613 ([^81^BrM + Na]^+^, 100%); HRMS (ESI +ve TOF) calcd for ^79^BrC_26_H_25_N_2_O_7_SNa 611.0464, found 611.0435 ([M + Na]^+^).

4-Methoxybenzyl (6*R*,7*R*)-3-(acetoxymethyl)-7-(((*E*)-3,5-di-*tert*-butyl-4-hydroxy-benzylidene)amino)-8-oxo-5-thia-1-azabicyclo[4.2.0]oct-2-ene-2-carboxylate **15b**

To a suspension of crude product **14** (0.757 mmol, prepared from 206.1 mg of 7-ACA **9**) in CH_2_Cl_2_ (2 mL) under an Ar atmosphere at rt 4-methoxybenzyl 2,2,2-trichloroacetimidate (204.3 µL, 0.984 mmol, 1.3 equiv.) was added, and the resulting mixture was stirred at rt for 48 h. The color of the reaction turned from bright yellow to brown, and the completion of the reaction was indicated using MS analysis. The reaction mixture was filtered and concentrated in vacuo to give the crude product **15** as a sticky brown gum. A portion of this filtrate was used for NMR analysis, which indicated a mixture of the titled compound, DMA, and 3,5-di-*tert*-butyl-4-hydroxybenzaldehyde. Further purification using flash chromatography resulted in decomposition. ^1^H NMR (500 MHz, CD_3_OD) δ 8.42 (s, 1H, H9), 7.63 (s, 2H, H10), 7.35 (d, *J* = 8.7 Hz, 2H, ArCH, PMB), 6.90 (d, *J* = 8.8 Hz, 2H, ArCH, PMB), 5.40 (d, *J* = 5.2 Hz, 1H, H7), 5.25–5.15 (m, 3H, H6 and O-CH_2_), 4.99 (d, *J* = 13.0 Hz, 1H, H3′_A_), 4.76 (d, *J* = 13.0 Hz, 1H, H3′_B_), 3.78 (s, 3H, OCH_3_), 3.64 (d, *J* = 18.4 Hz, 1H, H4_A_), 3.46 (d, *J* = 18.4 Hz, 1H, H4_B_), 2.02 (s, 3H, C4′-CH_3_), 1.44 (s, 18H, 2 × C-(CH_3_)_3_); (ESI + ve) *m/z* 609 ([M + H]^+^, 81%) and 631([M + H]^+^, 76%); (ESI − ve) *m/z* 607 ([M – H]^–^, 100%).].

Benzhydryl (6*R*,7*S*)-3-(acetoxymethyl)-7-(2-bromoacetamido)-7-methoxy-8-oxo-5-thia-1-azabicyclo[4.2.0]oct-2-ene-2-carboxylate **16b**

The crude compound **15b** was suspended in MeOH (5 mL) at −20 °C under an Ar atmosphere. To the suspension DDQ (171.8 mg, 0.757 mmol, 1.0 equiv.) was added, and the reaction mixture was stirred at −20 °C for 45 min. The completion of the reaction was indicated via TLC analysis (TLC (EtOAc/hexane—2:3): R*_f_* = 0.39). The reaction mixture was concentrated in vacuo and purified via column chromatography over SiO_2_. Prior to the purification, a series of solvents in a EtOAc/hexane system were prepared (using 3.6 mmol crude product as an example: 100 mL—1:19, 200 mL—1:9, 100 mL—3:17, 100 mL—1:4, 100 mL—11:39, 100 mL—6:19, 100 mL—13:37, 100 mL—7:18, 200 mL—3:7, and 200 mL—7:20) and chilled at −20 °C overnight. On the following day, an appropriately sized column was packed with a SiO_2_ slurry in hexane and chilled at −20 °C until ready to use. Once the crude product was loaded onto the column, the pre-chilled solvents was kept cool on an ice bath and the flow rate was accelerated by using a stream of compressed air to ensure the entire purification process was kept within 30 min. Fractions with R*_f_* value of 0.39 (TLC (EtOAc/hexane—2:3)) were combined and concentrated in vacuo to give the title compound as a hydroscopic, pale-yellow foam (204.1 mg, 0.320 mmol, 42% from 7-ACA). ${\left[\alpha \right]}_{D}^{22}$ +89.2 (*c* 0.27, CH_2_Cl_2_); ^1^H NMR (400 MHz, CDCl_3_) δ 8.51 (s, 1H, H9), 7.69 (s, 2H, H10), 7.39 (d, *J* = 8.8 Hz, 2H, ArCH, PMB), 6.90 (d, *J* = 8.8 Hz, 2H, ArCH, PMB), 5.63 (s, 1H, OH), 5.27 (ABq, *J*_A,B_ = 11.6 Hz, 2H, O-CH_2_), 5.05 (s, 1H, H6), 4.87 (ABq, *J*_A,B_ = 13.2 Hz, 2H, H), 3.81 (s, 3H, OCH_3_, PMB), 3.55 (s, 3H,OCH_3_, β-lactam), 3.38 (ABq, *J*_A,B_ = 18.3 Hz, 2H, H4), 2.03 (s, 3H, C4′-CH_3_), 1.46 (s, 18H, C-(CH_3_)_3_); ^13^C NMR (101 MHz, CDCl3) δ 170.6 (C4′), 165.8 (C9), 163.4 (C8), 161.5 (C2′), 160.0 (Cq, PMB), 157.9 (C-OH), 136.3 (*C*-C(CH_3_)_3_), 130.7 (ArCH, PMB), 126.9 (C10), 126.5 (C-C9), 126.2 (Cq, PMB), 124.2 (C2), 114.0 (C12), 104.6 (C7), 68.1 (CH_2_, PMB ester), 64.5 (C6), 63.1 (C3′), 55.3 (OCH_3_, PMB), 53.5 (OCH_3_, β-lactam), 34.4 (*C*(CH_3_)_3_), 30.2 (C(*C*H_3_)_3_), 26.6 (C4), 20.7 (CH_3_, acetate); C3 resonance was not observed in the ^13^C NMR or HMBC spectra; IR (cm^−1^) $\bar{\nu }$_max_ 3613 (w), 2958 (m), 1772 (s, β-lactam C=O), 1737 (s, C=O, acetate), 1631 (m), 1516 (m), 1429 (m), 1389 (m), 1302 (w), 1235 (s, C-O stretching, acetate), 1176 (m), 1129 (m), 1093 (m), 1034 (m), 888 (w), 827 (m), 775 (w); MS (ESI +ve) *m/z* 639 ([M + H]^+^, 23%), 661 ([M + Na]^+^, 100%), 1300 ([2M + Na]^+^, 77%); (ESI − ve) *m/z* 637 ([M − H]^−^, 100%); HRMS (ESI + ve TOF) calcd for C_34_H_43_N_2_O_8_S 639.2740, found 639.2752 ([M + H]^+^).

4-Methoxybenzyl (6*R*,7*S*)-3-(acetoxymethyl)-7-amino-7-methoxy-8-oxo-5-thia-1 azabicyclo[4.2.0]oct-2-ene-2-carboxylate **8**

To a solution of **16b** (333.5 mg, 0.487 mmol) in EtOAc (2.4 mL) a solution of Girard-T reagent (163.3 mg, 0.974 mmol, 2.0 equiv.) in MeOH (2.9 mL) at rt was added, and the resulting solution was stirred for 3.5 h until the completion of the reaction was indicated via TLC analysis (TLC (EtOAc/hexane—1:1): R*_f_* = 0.24). Upon completion, the reaction mixture was diluted with EtOAc (50 mL) and poured into water (50 mL), which was extracted using additional EtOAc (50 mL × 2). The combined EtOAc layer was washed with brine (70 mL), dried over anhydrous Na_2_SO_4_, filtered, and concentrated in vacuo to give the crude product as a dark green gum. A portion of this crude product (0.216 mmol) was purified via flash chromatography over SiO_2_ (EtOAc/hexane—1:10–2:3) to give the titled compound as a green gum (15.4 mg, 0.0365 mmol, 17%). ${\left[\alpha \right]}_{D}^{23}$ + 48.74 (*c* 0.77, CH_2_Cl_2_); ^1^H NMR (500 MHz, CDCl_3_) δ 7.35 (d, *J* = 8.4 Hz, 3H, ArCH, PMB), 6.88 (d, *J* = 8.5 Hz, 2H, ArCH, PMB), 5.23 (ABq, *J*_A,B_ = 12.3 Hz, 2H, O-CH_2_), 5.02 (d, *J* = 13.4 Hz, 1H, H3′_A_), 4.85–4.76 (m, 2H, H6 and H3′_B_), 3.80 (s, 3H, OCH_3_, PMB), 3.53–3.25 (m, 5H, H4 and OCH_3_, β-lactam), 2.04 (s, 3H, C4′-CH_3_); ^13^C NMR (126 MHz, CDCl_3_) δ 170.8 (C4′), 164.1 (C8), 161.5 (C2′), 160.1 (Cq, PMB), 130.8 (ArCH, PMB), 127.1 (Cq, PMB), 126.7 (C2), 114.1 (ArCH, PMB), 98.6 (C7), 68.2 (CH_2_, PMB ester), 63.9 (C6), 63.0 (C3′), 55.5 (OCH_3_, PMB), 52.6 (OCH_3_, β-lactam), 26.8 (C4), 20.9 (CH_3_, acetate); C3 resonance was not observed in the ^13^C NMR or HMBC spectra; IR (cm^−1^) $\bar{\nu }$_max_ 3307 (w), 2956 (w), 2930 (w), 1777 (s, β-lactam C=O), 1725 (s, C=O, acetate), 1612 (m), 1514 (s), 1455 (w), 1379 (m), 1354 (m), 1219 (s, C-O stretching, acetate), 1174 (s), 1112 (m), 1026 (s), 821 (s), 728 (m); MS (ESI + ve) *m/z*; 445 ([M + Na]^+^, 100%), 468 ([M + 2Na − H]^+^, 83%), 867 (2M + Na]^+^, 35%); HRMS (ESI + ve TOF) calcd for C_19_H_22_N_2_O_7_SNa 455.1045, found 455.1063 ([M + Na]^+^).

4-Methoxybenzyl (6*R*,7*S*)-3-(acetoxymethyl)-7-(2-bromoacetamido)-7-methoxy-8-oxo-5-thia-1-azabicyclo[4.2.0]oct-2-ene-2-carboxylate **17b**

To a solution of the crude compound **7b** (0.320 mmol, prepared from 204.1 mg of **16b**) in anhydrous CH_2_Cl_2_ (3 mL) under N_2_ at –20 °C (NaCl/ice) pyridine (80.2 μL, 0.992 mmol, 3.1 equiv.) was added and the resulting solution was stirred for 2 min. Bromoacetyl bromide (75.3 μL, 0.864 mmol, 2.7 equiv.) was then added dropwise to the solution. The reaction mixture was stirred at –20 °C for 2 h, at which point the reaction was shown to be complete via TLC analysis (TLC (EtOAc/hexane—2:3): R*_f_* = 0.32). The reaction mixture was poured into EtOAc (5 mL); washed sequentially with HCl (1.0 M—5 × 50 mL), saturated NaHCO_3_ (5 × 50 mL), and brine (100 mL); dried over anhydrous Na_2_SO_4_, filtered, and concentrated in vacuo to give a brown residue. The crude product was purified via flash chromatography over SiO_2_ (EtOAc/hexane—1:19–9:11) to give the titled compound as an orange gum (67.5 mg, 0.126 mmol, 39% over from **16b**). ${\left[\alpha \right]}_{D}^{24}$ +122.96 (*c* 1.08, CH_2_Cl_2_); ^1^H NMR (400 MHz, CDCl_3_) δ 7.36 (d, *J* = 8.7 Hz, 2H, ArCH, PMB), 6.89 (d, *J* = 8.7 Hz, 2H, ArCH, PMB), 5.25 (ABq, *J*_A,B_ = 11.9 Hz, 2H, H9), 5.07 (s, 1H, H6), 4.94 (ABq, *J*_A,B_ = 13.7 Hz, 2H, H3′), 3.93 (ABq, *J*_A,B_ = 13.7 Hz, 2H, Br-CH_2_), 3.81 (s, 3H, OCH_3_, PMB), 3.56 (s, 3H, OCH_3_, β-lactam), 3.40 (ABq, *J*_A,B_ = 18.0 Hz, 2H, H4), 2.05 (s, 3H, C4′-CH_3_), NH signal was not observed; ^13^C NMR (101 MHz, CDCl_3_) δ 170.5 (C4′), 166.7 (C=O, amide), 160.9 (C2′), 160.1 (C8), 159.9 (Cq, PMB), 130.6 (ArCH, PMB), 126.8 (ArCH, PMB), 126.0 (C2), 114.0 (C12), 95.6 (C7), 68.1 (CH_2_, PMB ester), 62.6 (C3′), 55.3 (OCH_3_, PMB), 53.9 (OCH_3_, β-lactam), 27.9 (Br-CH_2_), 26.9 (C4), 20.7 (CH_3_, acetate); C3 resonance was not observed in the ^13^C NMR or HMBC spectra; IR (cm^−1^) $\bar{\nu }$_max_ 3279 (w), 2962 (w), 2838 (w), 1777 (s, β-lactam C=O), 1735 (s, C=O, acetate), 1613 (m), 1516 (s), 1392 (m), 1240 (s, C-O stretching, acetate), 1178 (m), 1130 (m), 1088 (m), 10320 (m), 853 (w); MS (ESI + ve) *m/z* 565 ([^79^BrM + Na]^+^, 86%), 567 ([^81^BrM + Na]^+^, 100%), 588 ([^79^BrM + 2Na − H]^+^, 21%), 590 ([^81^BrM + 2Na − H]^+^, 24%); HRMS (ESI + ve TOF) calcd for ^79^BrC_21_H_23_N_2_O_8_SNa 565.0256, found 565.0266 ([M + Na]^+^).

(6*R*,7*S*)-3-(Acetoxymethyl)-7-(2-bromoacetamido)-7-methoxy-8-oxo-5-thia-1-azabicyclo[4.2.0]oct-2-ene-2-carboxylic acid **18**

Method 1—prepared from compound **17a**. To a solution of compound **17a** (76.5 mg, 0.130 mmol) in anisole (451 μL, 4.15 mmol, 32 equiv.) TFA was added (745 μL, 9.73 mmol, 75 equiv.), and the solution was stirred at rt for 10 min. The reaction mixture was poured into EtOAc (50 mL) and extracted with saturated NaHCO_3_ (3 × 50 mL). The combined aqueous layer was washed with EtOAc (2 × 50 mL). Additional EtOAc (200 mL) was added to a stirred solution of the aqueous layer, and the aqueous layer was acidified with conc. HCl to pH < 1. The two layers were then separated. The aqueous layer was extracted with EtOAc (2 × 50 mL), and the combined organic layer was washed sequentially with HCl (1.0 M—3 × 50 mL) and brine (50 mL), dried over anhydrous Na_2_SO_4_, filtered, and concentrated in vacuo to give a yellow gum. This crude product was redissolved in a minimum amount of EtOAc, and hexane was added dropwise to the vigorously stirred solution of EtOAc until precipitation started to occur. The mixture was stirred at rt overnight. The solvent was removed using a syringe and the precipitate was washed with hexanes (5 × 10 mL) then dried in vacuo to afford the titled compound as a yellow gum (41.0 mg, 0.0969 mmol, 75%).

Method 2—prepared from compound **17b**. To a solution of compound **17b** (67.5 mg, 0.124 mmol) in anhydrous CH_2_Cl_2_ (1 mL) at 0 °C TFA was slowly added (247 μL, 3.23 mmol, 26 equiv.). The resulting dark-pink solution was stirred at 0 °C for 30 min. Work-up and precipitation as in Method 1 gave the titled compound as a yellow gum (47.1 mg, 0.111 mmol, 90%). ${\left[\alpha \right]}_{D}^{25}$ +143.50 (*c* 1.41, MeOH); 1H NMR (400 MHz, CD_3_CN) δ 7.79 (s, 1H, NH), 5.06 (s, 1H, H6), 4.87 (ABq, *J*_A,B_ = 13.4 Hz, 2H, H3′), 3.89 (s, 2H, Br-CH_2_), 3.56 (d, *J* = 18.0 Hz, 1H, H4_A_), 3.50 (s, 3H, OCH_3_), 3.33 (d, *J* = 18.0 Hz, 1H, H4_B_), 2.02 (s, 3H, CH_3_, acetate); ^13^C NMR (101 MHz, CD_3_CN) δ 171.1 (C4′), 167.9 (C=O, amide), 162.4 (C2′), 160.9 (C8), 128.7 (C3), 126.1 (C2), 95.6 (C7), 64.2 (C6), 63.0 (C3′), 53.6 (O-CH_3_), 28.5 (Br-CH_2_), 26.6 (C4), 20.5 (CH_3_, acetate); IR (cm^−1^) $\bar{\nu }$_max_ 3538 (w), 3271 (w), 3022 (w), 1774 (s, β-lactam C=O),1728 (s, C=O, acetate), 1693 (s), 1539 (m), 1386 (m), 1235 (s, C-O stretching, acetate), 1134 (m), 1088 (m), 1024 (m); MS (ESI + ve) *m/z* 445 ([^79^BrM + Na]^+^, 93%), 447 ([^81^BrM + Na]^+^, 100%); HRMS (ESI + ve TOF) calcd for ^79^BrC_13_H_15_N_2_O_7_S_2_Na 444.9681, found 444.9699 ([M + Na]^+^).

(6*R*,7*S*)-3-(Acetoxymethyl)-7-(2-azidoacetamido)-7-methoxy-8-oxo-5-thia-1-azabicyclo [4.2.0]oct-2-ene-2-carboxylic acid **19**

To a solution of compound **18** (36.7 mg, 0.0867 mmol) in DMF (0.3 mL) at −15 °C sodium azide (28.2 mg, 0.434 mmol, 5.0 equiv.) was added, and the solution was stirred for 24 h, at which point the reaction was shown to be complete via MS analysis. The reaction mixture was diluted with distilled H_2_O (20 mL), to which EtOAc (20 mL) was added. The resulting mixture was stirred vigorously, and the aqueous layer was acidified with conc. HCl to pH < 1. The two layers were separated, and the aqueous layer was extracted with EtOAc (3 × 20 mL). The combined organic layer was washed sequentially with distilled H_2_O (3 × 20 mL) and brine (40 mL), dried over anhydrous Na_2_SO_4_, filtered, and concentrated in vacuo to give a sticky gum. This crude product was dissolved in a minimum amount of EtOAc, and hexane was added dropwise to the vigorously stirred solution until the precipitation started to occur. The mixture was stirred at rt overnight. The solvent was removed using a syringe and the precipitate was washed with hexanes (5 × 10 mL), then dried in vacuo to afford the titled compound as a pale-yellow gum (27.1 mg, 0.0703 mmol, 81%). ${\left[\alpha \right]}_{D}^{25}$ +158.3 (*c* 0.77, MeOH); ^1^H NMR (400 MHz, CD_3_CN) δ 7.58 (s, 1H, NH), 5.06 (s, 1H, H6), 4.88 (ABq, *J*_A,B_ = 13.3 Hz, 2H, H3′), 3.95 (s, 2H, Br-CH_2_), 3.57 (d, *J* = 18.0 Hz, 1H, H4_A_), 3.50 (s, 3H, OCH_3_), 3.34 (d, *J* = 18.0 Hz, 1H, H4_B_), 2.02 (s, 3H, CH_3_, acetate); ^13^C NMR (101 MHz, CD_3_CN) δ 171.1 (C4′), 169.2 (C=O, amide), 162.5 (C2′), 161.0 (C8), 128.6 (C3), 126.3 (C2),96.3 (C7), 64.1 (C6), 63.0 (C3′), 53.6 (O-CH_3_), 51.6 (N3-CH_2_), 26.7 (C4), 20.5 (CH_3_, acetate); IR (cm^−1^) $\bar{\nu }$_max_ 3385 (m), 3223 (m), 3026 (w), 2111 (N=N=N stretching), 1772 (s, β-lactam C=O), 1705 (s) 1514 (m), 1424 (m), 1385 (m), 1230 (s, C-O stretching, acetate), 1134 (m), 1087 (m), 1026 (m), 551 (w); MS (ESI +ve) *m/z* 403 ([M+NH_4_]^+^, 100%), 408 ([M + Na]^+^, 79%), 431 ([M + 2Na − H]^+^, 74%), 449 ([M + MeCN + Na]^+^, 20%); (ESI − ve) *m/z* 384 ([M − H]^−^, 37%), 420 ([M + Cl]^−^, 65%), 422 ([M + K − 2H]^−^, 28%), 498 ([M + TFA − H]^−^, 64%); HRMS (ESI + ve TOF) calcd for C_13_H_15_N_5_O_7_S_2_Na 408.0590, found 408.0581 ([M + Na]^+^).

For NMR assignments of compounds **20** and **21a**–**h**, the following numbering system has been used.


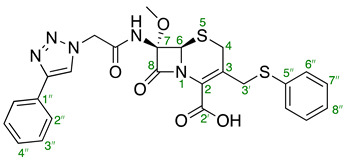

(6*R*,7*S*)-3-(Acetoxymethyl)-7-methoxy-8-oxo-7-(2-(4-phenyl-1*H*-1,2,3-triazol-1-yl)acetamido)-5-thia-1-azabicyclo[4.2.0]oct-2-ene-2-carboxylic acid **20**

To a reaction vessel charged consecutively with azide **19** (27.1 mg, 0.0703 mmol), CuSO_4_·5H_2_O (3.50 mg, 0.0141 mmol, 0.2 equiv.) and sodium ascorbate (5.55 mg, 0.0281 mmol, 0.4 equiv.) a mixture of *t*-BuOH and H_2_O (*t*-BuOH/H_2_O—1:1, 0.8 mL) was added. To this stirred mixture, phenylacetylene (23.3 μL, 0.211 mmol, 3.0 eq.) was then added, and the reaction mixture was stirred at 30 ℃ for 24 h. The reaction mixture was diluted with EtOAc (20 mL), washed with saturated aqueous NH_4_Cl solution (20 mL), dried over anhydrous MgSO_4_, filtered, and concentrated in vacuo. The obtained residue was then dissolved in a minimum amount of EtOAc, and to this vigorously stirred solution hexanes were added dropwise until precipitation started to occur. The mixture was stirred at rt overnight. The solvent was removed using a syringe and the precipitate was washed with hexanes (5 × 10 mL), then dried in vacuo to afford the titled compound as a thin, yellow film (22.5 mg, 0.0461 mmol, 66%). ${\left[\alpha \right]}_{D}^{25}$ +103.1 (*c* 0.54, MeOH); ^1^H NMR (400 MHz, CD_3_CN) δ 8.16 (s, 1H, CH, triazole), 7.93 (s, 1H, NH), 7.89–7.84 (m, 2H, H2″), 7.46 (t, *J* = 7.5 Hz, 2H, H3″), 7.39–7.33 (m, 1H, H4″), 5.28 (s, 2H, CH_2_-triazole), 5.06 (s, 1H, H6), 4.88 (ABq, *J*_A,B_ = 13.3 Hz, 2H, H3′), 3.59–3.50 (m, 4H, H4_A_ and O-CH_3_), 3.32 (d, *J* = 17.9 Hz, 1H, H4_B_), 2.02 (s, 3H, CH_3_, acetate); ^13^C NMR (101 MHz, CD_3_CN) δ 171.1 (C4′), 167.2 (C=O, amide), 162.6 (C2′), 160.9 (C8), 147.7 (Cq, triazole), 131.3 (C1″), 129.5 (C3″), 128.71 (C3) 128.68 (C4″), 126.3 (C2), 126.1 (C2″), 122.9 (CH, triazole), 96.4 (C7), 64.1 (C6), 63.0 (C3′), 53.8 (O-CH_3_), 52.4 (CH_2_-triazole), 26.7 (C4), 20.5 (CH_3_, acetate); IR (cm^−1^) $\bar{\nu }$_max_ 3288 (w), 2946 (w), 1779 (s, β-lactam C=O), 1708 (s), 1533 (m), 1442 (w), 1382 (m), 1231 (s, C-O stretching, acetate), 1136 (m), 1087 (m) 1028 (m), 768 (s), 696 (m); MS (ESI + ve) *m/z* 488 ([M + H]^+^, 100%), 510 ([M + Na]^+^, 12%), 975 ([2M + H]^+^, 7%); (ESI − ve) *m/z* 486 ([2M − H]^−^, 65%); HRMS (ESI + ve TOF) calcd for C_17_H_17_N_5_O_7_SNa 510.1059, found 510.1050 ([M + Na]^+^).

The General Procedure for the Pd/BIPHEPHOS reaction for the preparation of the cephamycin C-3 thiol derivatives **21a**–**h**. Synthesis of the thiomethyl compound **21a** is given as an example.

(6*R*,7*S*)-7-Methoxy-3-(((4-methoxyphenyl)thio)methyl)-8-oxo-7-(2-(4-phenyl-1*H*-1,2,3-triazol-1-yl)acetamido)-5-thia-1-azabicyclo[4.2.0]oct-2-ene-2-carboxylic acid **21a**

This compound was prepared following a procedure reported in the literature with some modifications [27]. To a flame-dried flask Pd_2_(dba)_3_·CHCl_3_ (10.7 mg, 0.0103 mmol, 10 mol%), BIPHEPHOS (17.0 mg, 0.0216 mmol, 21 mol%), and anhydrous MeCN (2 mL) were added under Ar at 50 °C. The resulting suspension was stirred at 50 °C for 30 min until it turned into a bright-yellow solution. The reaction flask was allowed to cool to rt and was then placed in a sonicator with the water temperature at 30–35 °C. Compound **20** (50.4 mg, 0.103 mmol) and 4-methoxybenzenethiol (25.4 μL, 0.207 mmol, 2.0 equiv.) were added to the reaction flask. The resulting mixture was sonicated at 30–35 °C until the completion of the reaction (10 h) was indicated using MS analysis. The reaction mixture was poured into EtOAc (10 mL) and extracted with NaHCO_3_ (3 × 10 mL). The combined aqueous layer was acidified with conc. HCl to pH = 3 and extracted with EtOAc (3 × 10 mL). The combined EtOAc fraction was then washed with HCl (1.0 M—3 × 10 mL) and brine (20 mL), dried over anhydrous Na_2_SO_4_, filtered, and concentrated in vacuo to give a yellow gum. This crude product was purified via semi-preparative RP-HPLC using a MeCN/H_2_O gradient mobile phase containing 0.01% TFA (MeCN/H_2_O—3:5–7:10, 15 min, injection volume = 200 μL) at a flow rate of 3.8 mL/min to give the titled compound as a pale-yellow oil (13.6 mg, 0.0240 mmol, 23%). Compound purity by HPLC (see Supplementary Materials): 99.8%, 279 nm; ${\left[\alpha \right]}_{D}^{24}$ +22.6 (*c* 0.46, MeCN); ^1^H NMR (400 MHz, CD_3_CN) δ 8.16 (s, 1H, CH, triazole), 7.87 (dd, *J* = 8.4, 1.3 Hz, 2H, H2″), 7.45 (t, *J* = 7.5 Hz, 2H, H3″), 7.42–7.33 (m, 3H, H4″ and H6″), 6.85 (d, *J* = 8.8 Hz, 2H, H7″), 5.28 (s, 2H, CH_2_-triazole), 4.97 (s, 1H, H6), 4.21 (d, *J* = 13.3 Hz, 1H, H3′_A_), 3.77 (s, 3H, OCH_3_, β-lactam), 3.71 (d, *J* = 13.3 Hz, 1H, H3′_B_), 3.55 (d, *J* = 13.3 Hz, 1H, H4_A_), 3.53 (s, 3H, Ar-OCH_3_), 3.35 (d, *J* = 16.5 Hz, 1H, H4_B_); NH resonance was not observed; ^13^C NMR (101 MHz, CD_3_CN) δ 167.2 (C=O, amide), 162.5 (C2′), 161.2 (C8), 160.9 (C8″), 148.0 (Cq, tetrazole), 137.3 (C3), 136.7 (C6″), 131.6 (C1″), 129.7 (C3″), 128.9 (C4″), 126.3 (C2″), 125.3 (C2), 124.4 (C5″), 123.1 (CH, triazole), 115.4 (C7″), 97.0 (C7), 65.9 (C6), 55.9 (OCH_3_, β-lactam), 54.0 (Ar-OMe), 52.8 (CH_2_-triazole), 38.8 (C3′), 29.48 (C4); IR (cm^−1^) $\bar{\nu }$_max_ 3267 (w), 2993 (w), 2839 (w), 1771 (s, β-lactam C=O), 1703 (s), 1591 (m), 1494 (s), 1465 (w), 1442 (w), 1286 (w), 1247 (s), 1181 (w), 1148 (w), 1106 (m), 1088 (m), 1026 (m), 830 (m), 766 (s), 696 (m); MS (ESI + ve) *m/z* 568 ([M + H]^+^, 40%), 590 ([M + Na]^+^, 53%); (ESI − ve) *m/z* 566 ([M − H]^−^, 63%), 680 ([M + TFA − H]^−^, 63%); HRMS (ESI − ve TOF) calcd for C_26_H_24_N_5_O_6_S_2_ 566.1168, found 566.1160 ([M − H]^−^).

(6*R*,7*S*)-3-(((4-Carboxyphenyl)thio)methyl)-7-methoxy-8-oxo-7-(2-(4-phenyl-1*H*-1,2,3-triazol-1-yl)acetamido)-5-thia-1-azabicyclo[4.2.0]oct-2-ene-2-carboxylic acid **21b**

This compound was prepared according to the General Procedure using **20** (50.0 mg, 0.103 mmol), Pd_2_(dba)_3_·CHCl_3_ (10.6 mg, 0.0103 mmol, 10 mol%), BIPHEPHOS (17.0 mg, 0.0216 mmol, 21 mol%), and 4-mercaptobenzoic acid (31.6 mg, 0.205 mmol, 2.0 equiv.) in MeCN (2 mL) with 19 h of reaction time. Work-up and purification via RP-HPLC (MeCN/H_2_O—1:1–9:11, 15 min, injection volume = 160 μL) as described above gave the titled compound as a pale-yellow oil (18.5 mg, 0.0318 mmol, 31%). Compound purity by HPLC (see Supplementary Materials): 98.7%, 254 nm; ${\left[\alpha \right]}_{D}^{24}$ +39.3 (*c* 0.67, MeCN); ^1^H NMR (400 MHz, CD_3_CN) δ 8.14 (s, 1H, CH, triazole), 7.99–7.82 (m, 4H, H2″ and H7″), 7.80 (s, 1H, NH), 7.54–7.40 (m, 4H, H3″ and H6″), 7.40–7.31 (m, 1H, H4″), 5.26 (s, 2H, CH_2_-triazole), 4.98 (s, 1H, H6), 4.31 (d, *J* = 13.4 Hz, 1H, H3′_A_), 4.01 (d, *J* = 13.4 Hz, 1H, H3′_B_), 3.54 (d, 1H, *J* = 16.9 Hz, H4_A_), 3.53 (s, 3H, O-CH_3_), 3.36 (d, *J* = 16.9 Hz, 1H, H4_B_); ^13^C NMR (101 MHz, CD_3_CN) δ 167.1 (C=O, amide or C9″), 167.0 (C9″ or C=O, amide), 162.5 (C2′), 161.0 (C8), 147.8 (Cq, tetrazole), 142.0 (C5″), 135.1 (C3), 131.3 (C1″), 130.7 (C3″), 130.5 (C7″), 129.5 (C6″), 128.9 (C4″), 128.7 (C8″), 126.1 (C2″), 125.5 (C2), 122.9 (CH, triazole), 96.7 (C7), 65.4 (C6), 53.8 (O-CH_3_), 52.5 (CH_2_-triazole), 35.9 (C3′), 29.0 (C4); IR (cm^−1^) $\bar{\nu }$_max_ 3527 (w), 3032 (w), 2940 (w), 1772 (s, β-lactam C=O), 1701 (s), 1592 (m), 1560 (w), 1402 (w), 1364 (w), 1235 (s), 1182 (w), 1110 (m), 1187 (m), 1015 (w), 851 (w), 796 (w), 765 (s), 695 (m); MS (ESI + ve) *m/z* 582 ([M + H]^+^, 19%), 604 ([M + Na]^+^, 42%); (ESI − ve) *m/z* 580 ([M − H]^−^, 72%), 680 ([M + TFA − H]^−^, 19%); HRMS (ESI − ve TOF) calcd for C_26_H_22_N_5_O_6_S_2_ 580.0961, found 580.0952 ([M − H]^−^).

(6*R*,7*S*)-3-(((4-Cyanophenyl)thio)methyl)-7-methoxy-8-oxo-7-(2-(4-phenyl-1*H*-1,2,3-triazol-1-yl)acetamido)-5-thia-1-azabicyclo[4.2.0]oct-2-ene-2-carboxylic acid **21c**

This compound was prepared according to the General Procedure using **20** (50.6 mg, 0.104 mmol), Pd_2_(dba)_3_·CHCl_3_ (10.8 mg, 0.0104 mmol, 10 mol%), BIPHEPHOS (17.2 mg, 0.0218 mmol, 21 mol%) and 4-mercaptobenzonitrile (28.1 mg, 0.208 mmol, 2.0 equiv.) in MeCN (2 mL) with 13 h of reaction time. Work-up and purification via RP-HPLC (MeCN/H2O —2:3–7:10, 15 min, injection volume = 200 μL) as described above gave the titled compound as a thin, transparent film (14.8 mg, 0.0263 mmol, 25%). Compound purity by HPLC (see Supplementary Materials): 99.9%, 254 nm; ${\left[\alpha \right]}_{D}^{24}$ +35.5 (*c* 0.42, MeCN); ^1^H NMR (400 MHz, CD_3_CN) δ 8.14 (s, 1H, CH, triazole), 7.86 (d, *J* = 7.0 Hz, 2H, H2″), 7.81 (s, 1H, NH), 7.61 (d, *J* = 8.1 Hz, 2H, H7″), 7.46 (dd, *J* = 7.9, 8.4 Hz, 4H, H3″ and H6″), 7.40–7.31 (m, 1H, H4″), 5.26 (s, 2H, CH_2_-triazole), 4.98 (s, 1H, H6), 4.29 (d, *J* = 13.4 Hz, 1H, H3′_A_), 4.05 (d, *J* = 13.4 Hz, 1H, H3′_B_), 3.53 (d, 1H, *J* = 16.9 Hz, H4_A_), 3.52 (s, 3H, O-CH_3_), 3.35 (d, *J* = 16.9 Hz, 1H, H4_B_); ^13^C NMR (101 MHz, CD_3_CN) δ 167.0 (C=O, amide), 161.0 (C8), 147.8 (Cq, tetrazole), 142.8 (C5″), 134.0 (C3), 133.1 (C7″), 131.4 (C1″), 130.6 (C3″), 129.5 (C6″), 128.7 (C4″), 126.1 (C2″), 125.4 (C2), 122.9 (CH, triazole), 119.1 (C8″), 110.2 (C≡N), 96.6 (C7), 65.4 (C6), 53.8 (O-CH_3_), 52.5 (CH_2_-triazole), 35.6 (C3′), 28.9 (C4); C2′ resonance was not observed in the ^13^C NMR or HMBC spectra; IR (cm^−1^) $\bar{\nu }$_max_ 3500 (w), 3279 (w), 2228 (s, C≡N stretching), 1771 (s, β-lactam C=O), 1705, 1592 (m), 1537 (w), 1468 (w), 1372 (m), 1154 (w), 1017 (s), 827 (m), 767 (s), 696 (m), 549 (m); MS (ESI + ve) *m/z* 585 ([M + Na]^+^, 53%), 607 ([M + 2Na − H]^+^, 19%), 639 ([M + 2K − H]^+^, 37%); (ESI − ve) *m/z* 561 ([M − H]^−^, 53%), 675 ([M + TFA − H]^−^, 21%); HRMS (ESI +ve TOF) calcd for C_26_H_23_N_6_O_5_S_2_ 563.1171, found 563.1166 ([M + H]^+^).

(6*R*,7*S*)-3-(((4-Fluorophenyl)thio)methyl)-7-methoxy-8-oxo-7-(2-(4-phenyl-1*H*-1,2,3-triazol-1-yl)acetamido)-5-thia-1-azabicyclo[4.2.0]oct-2-ene-2-carboxylic acid **21d**

This compound was prepared according to the General Procedure using **20** (51.0 mg, 0.105 mmol), Pd_2_(dba)_3_·CHCl_3_ (10.9 mg, 0.0105 mmol, 10 mol%), BIPHEPHOS (17.3 mg, 0.0220 mmol, 21 mol%), and 4-fluorobenzenethiol (22.4 μL, 0.209 mmol, 2.0 equiv.) in MeCN (2 mL) with 23 h of reaction time. Work-up and purification via RP-HPLC (MeCN/H_2_O—13:7–7:10, 15 min, injection volume = 120 μL) as described above gave the titled compound as a pale-yellow oil (7.3 mg, 0.0131 mmol, 13%). Compound purity by HPLC (see Supplementary Materials): 99.6%, 279 nm; ${\left[\alpha \right]}_{D}^{24}$ +41.3 (*c* 0.20, MeCN); ^1^H NMR (400 MHz, CD_3_CN) δ 8.15 (s, 1H, CH, triazole), 7.86 (d, *J* = 7.0 Hz, 2H, H2″), 7.49–7.42 (m, 4H, H3″ and H6″), 7.36 (t, *J* = 7.3 Hz, 1H, H4″), 7.05 (t, *J* = 8.8 Hz, 2H, H7″), 5.27 (s, 2H, CH_2_-triazole), 4.97 (s, 1H, H6), 4.24 (d, *J* = 13.4 Hz, 1H, H3′_A_), 3.79 (d, *J* = 13.4 Hz, 1H, H3′_B_), 3.53 (d, 1H, *J* = 16.6 Hz, H4_A_), 3.52 (s, 3H, O-CH_3_), 3.35 (d, *J* = 16.6 Hz, 1H, H4_B_), NH resonance was not observed; ^13^C NMR (101 MHz, CD_3_CN) δ 167.0 (C=O, amide), 163.3 (d, *J*_C,F_ = 246.0 Hz, C8″), 162.5 (C2′), 161.0 (C8), 147.8 (Cq, tetrazole), 136.5 (d, *J*_C,F_ = 8.5 Hz, C6″), 136.1 (C3), 131.4 (C1″), 129.5 (C3″), 128.7 (C4″), 126.1 (C2″), 125.5 (C2), 122.9 (CH, triazole), 116.55 (d, *J*_C,F_ = 22.1 Hz, C7″), 96.7 (C7), 65.7 (C6), 53.8 (O-CH_3_), 52.5 (CH_2_-triazole), 38.1 (C3′), 29.1 (C4), C5″ resonance was not observed in the ^13^C NMR or HMBC spectra; IR (cm^−1^) $\bar{\nu }$_max_ 3283 (w), 3144 (w), 2942 (w), 1768 (s, β-lactam C=O), 1699 (s), 1589 (m), 1534 (m), 1490 (s), 1370 (m), 1220 (s, C-F stretching), 1156 (m), 1109 (m), 1088 (m), 1016 (w), 832 (m, C-F), 766 (s), 695 (m), 629 (w), 517 (w); MS (ESI +ve) *m/z* 556 ([M + H]^+^, 28%), 578 ([M + Na]^+^, 58%); (ESI − ve) *m/z* 554 ([M − H]^−^, 67%), 668 ([M + TFA − H]^−^, 100%); HRMS (ESI − ve TOF) calcd for C_25_H_21_N_5_O_5_S_2_F 554.0968, found 554.0976 ([M − H]^−^).

(6*R*,7*S*)-7-Methoxy-8-oxo-7-(2-(4-phenyl-1*H*-1,2,3-triazol-1-yl)acetamido)-3-((p-tolylthio)methyl)-5-thia-1-azabicyclo[4.2.0]oct-2-ene-2-carboxylic acid **21e**

This compound was prepared according to the General Procedure using **20** (51.3 mg, 0.105 mmol), Pd_2_(dba)_3_·CHCl_3_ (10.9 mg, 0.0105 mmol, 10 mol%), BIPHEPHOS (17.4 mg, 0.0221 mmol, 21 mol%), and 4-methylbenzenethiol (26.1 mg, 0.210 mmol, 2.0 equiv.) in MeCN (2 mL) with 13 h of reaction time. Work-up and purification via RP-HPLC (MeCN/H_2_O—13:7–7:10, 15 min, injection volume = 120 μL) as described above gave the titled compound as a pale-yellow oil (16.0 mg, 0.0290 mmol, 28%). Compound purity by HPLC (see Supplementary Materials): 99.9%, 254 nm; ${\left[\alpha \right]}_{D}^{24}$ +33.7 (*c* 0.33, MeCN); ^1^H NMR (400 MHz, CD_3_CN) δ 8.15 (s, 1H, CH, triazole), 7.89–7.84 (m, 2H, H2″), 7.81 (s, 1H, NH), 7.45 (t, *J* = 7.5 Hz, 2H, H3″), 7.36 (t, *J* = 7.5 Hz, 1H, H4″), 7.31 (d, *J* = 8.0 Hz, 2H, H6″), 7.12 (d, *J* = 7.9 Hz, 2H, H7″), 5.27 (s, 2H, CH_2_-triazole), 4.96 (s, 1H, H6), 4.22 (d, *J* = 13.3 Hz, 1H, H3′_A_), 3.81 (d, *J* = 13.3 Hz, 1H, H3′_B_), 3.52 (d, 1H, *J* = 16.7 Hz, H4_A_), 3.51 (s, 3H, O-CH_3_), 3.33 (d, *J* = 16.7 Hz, 1H, H4_B_), 2.30 (s, 3H, CH_3_); ^13^C NMR (101 MHz, CD_3_CN) δ 167.0 (C=O, amide), 162.4 (C2′), 161.0 (C8), 147.8 (Cq, tetrazole), 138.7 (C8″), 136.5 (C3), 133.5 (C6″), 131.4 (C1″), 130.8 (C5″), 130.3 (C7″), 129.5 (C3″), 128.7 (C4″), 126.1 (C2″), 125.1 (C2), 122.9 (CH, triazole), 96.7 (C7), 65.6 (C6), 53.8 (O-CH_3_), 52.5 (CH_2_-triazole), 37.7 (C3′), 29.1 (C4), 20.8 (CH_3_); IR (cm^−1^) $\bar{\nu }$_max_ 3519 (w), 3282 (w), 3024 (w), 1770 (s, β-lactam C=O), 1703 (s), 1536 (m), 1492 (w), 1442 (w), 1373 (m), 1233 (m), 1153 (w), 1088 (m), 1018 (m), 810 (m), 766 (s), 695 (m), 503 (w); MS (ESI + ve) *m/z* 552 ([M + H]^+^, 42%), 574 ([M + Na]^+^, 56%); (ESI − ve) *m/z* 550 ([M − H]^−^, 93%), 664 ([M + TFA − H]^−^, 40%); HRMS (ESI − ve TOF) calcd for C_26_H_24_N_5_O_5_S_2_ 550.1219, found 550.1227 ([M − H]^−^).

(6*R*,7*S*)-7-Methoxy-3-(((4-(methylthio)phenyl)thio)methyl)-8-oxo-7-(2-(4-phenyl-1*H*-1,2,3-triazol-1-yl)acetamido)-5-thia-1-azabicyclo[4.2.0]oct-2-ene-2-carboxylic acid **21f**

This compound was prepared according to the General Procedure using **20** (51.1 mg, 0.105 mmol), Pd_2_(dba)_3_·CHCl_3_ (10.9 mg, 0.0105 mmol, 10 mol%), BIPHEPHOS (17.3 mg, 0.0220 mmol, 21 mol%), and 4-(methylsulfanyl)thiophenol (20.2 μL, 0.210 mmol, 2.0 equiv.) in MeCN (2 mL) with 13 h of reaction time. Work-up and purification via RP-HPLC (MeCN/H_2_O—17:8–7:10, 15 min, injection volume = 120 μL) as described above gave the titled compound as a pale-yellow oil (9.3 mg, 0.0159 mmol, 15%). Compound purity by HPLC (see Supplementary Materials): 99.8%, 279 nm; ${\left[\alpha \right]}_{D}^{24}$ +22.2 (*c* 0.28, MeCN); ^1^H NMR (400 MHz, CD_3_CN) δ 8.15 (s, 1H, CH, triazole), 7.89–7.83 (m, 2H, H2″), 7.78 (s, 1H, NH), 7.45 (t, *J* = 7.6 Hz, 2H, H3″), 7.39–7.31 (m, 3H, H4″ and H6″), 7.18 (d, *J* = 8.5 Hz, 2H, H7″), 5.27 (s, 2H, CH_2_-triazole), 4.97 (s, 1H, H6), 4.23 (d, *J* = 13.4 Hz, 1H, H3′_A_), 3.81 (d, *J* = 13.4 Hz, 1H, H3′_B_), 3.53 (d, 1H, *J* = 16.7 Hz, H4_A_), 3.52 (s, 3H, O-CH_3_) 3.35 (d, *J* = 16.7 Hz, 1H, H4_B_), 2.45 (s, 3H, S-CH_3_); ^13^C NMR (101 MHz, CD_3_CN) δ 167.0 (C=O, amide), 162.4 (C2′), 161.0 (C8), 147.8 (Cq, tetrazole), 139.7 (C8″), 136.4 (C3), 134.2 (H6″), 131.4 (C1″), 130.2 (C5″), 129.5 (C3″), 128.7 (C4″), 127.0 (C7″), 126.1 (C2″), 125.2 (C2), 122.9 (CH, triazole), 96.7 (C7), 65.6 (C6), 53.8 (O-CH_3_), 52.5 (CH_2_-triazole), 37.8 (C3′), 29.2 (C4), 15.1 (CH_3_); IR (cm^−1^) $\bar{\nu }$_max_ 3507 (w), 3278 (w), 3139 (w), 1770 (s, β-lactam C=O), 1704 (s), 1625 (w), 1532 (m), 1478 (w), 1440 (w), 1370 (m), 1230 (s), 1152 (s), 1106 (w), 1087 (s), 1013 (w), 1000 (w), 812 (m), 766 (s), 695 (m), 504 (w); MS (ESI + ve) *m/z* 584 ([M + H]^+^, 30%), 606 ([M + Na]^+^, 51%), 622 ([M + K]^+^, 53%); (ESI −ve) *m/z* 582 ([M − H]^−^, 47%), 696 ([M + TFA − H]^−^, 93%); HRMS (ESI − ve TOF) calcd for C_26_H_24_N_5_O_5_S_3_ 582.0940, found 582.0931 ([M − H]^−^).

(6*R*,7*S*)-3-(((4-Ethylphenyl)thio)methyl)-7-methoxy-8-oxo-7-(2-(4-phenyl-1*H*-1,2,3-triazol-1-yl)acetamido)-5-thia-1-azabicyclo[4.2.0]oct-2-ene-2-carboxylic acid **21g**

This compound was prepared according to the General Procedure using **20** (58.7 mg, 0.120 mmol), Pd_2_(dba)_3_·CHCl_3_ (12.5 mg, 0.0120 mmol, 10 mol%), BIPHEPHOS (19.9 mg, 0.0253 mmol, 21 mol%), and 4-ethylbenzenethiol (33.3 μL, 0.241 mmol, 2.0 equiv.) in MeCN (2 mL) with 10 h of reaction time. Work-up and purification via RP-HPLC (MeCN/H2O—17:8–7:10, 15 min, injection volume = 120 μL) as described above gave the titled compound as a pale-yellow oil (8.4 mg, 0.0149 mmol, 12%). Compound purity by HPLC (see Supplementary Materials): 99.7%, 279 nm; ${\left[\alpha \right]}_{D}^{24}$ +65.5 (*c* 0.15, MeCN); ^1^H NMR (400 MHz, CD_3_CN) δ 8.15 (s, 1H, CH, triazole), 7.86 (d, *J* = 7.0 Hz, 1H, H2″), 7.78 (s, 1H, NH), 7.45 (t, *J* = 7.5 Hz, 2H, H3″), 7.40–7.30 (m, 3H, H4″ and H6″), 7.28–7.18 (m, *H6″ and *H7″), 7.15 (d, J = 8.2 Hz, 2H, H7″), 5.26 (s, 2H, CH_2_-triazole), 4.96 (s, 1H, H6), 4.93 (s, *H6), 4.22 (d, *J* = 13.2 Hz, 1H, H3′_A_), 4.16 (d, *J* = 13.1 Hz, *H3′_A_), 3.88 (d, *J* = 13.1 Hz, *H3′_B_), 3.82 (d, *J* = 13.3 Hz, 1H, H3′_B_), 3.52 (d, 1H, *J* = 16.8 Hz, H4_A_), 3.51 (s, 3H, O-CH_3_), 3.35 (d, *J* = 16.8 Hz, *H4_B_), 3.34 (d, *J* = 16.7 Hz, 1H, H4_B_), 2.79 (qd, *J* = 7.4, 2.1 Hz, *H9″), 2.61 (q, *J* = 7.6 Hz, 2H, H9″), 1.18 (t, *J* = 7.6 Hz, 3H, CH_3_), 1.18 (td, *J* = 7.5, 5.4 Hz, *CH_3_); ^13^C NMR (101 MHz, CD_3_CN) δ 167.0 (C=O, amide), 162.4 (C2′), 161.0 (C8), 147.8 (Cq, tetrazole), 145.0 (C8″), 136.5 (C3), 133.7 (*C6″), 133.5 (C6″), 131.4 (C5″), 131.1 (*C5″), 129.6 (*C7″), 129.5 (C3″), 129.2 (C7″), 128.7 (C4″), 126.1(C2″), 125.1 (C2), 122.9 (CH, triazole), 96.7 (C7), 65.6 (C6), 65.4 (*C6), 53.8 (O-CH3), 52.5 (CH2-triazole), 37.6 (C3′), 37.0 (*C3′), 29.2 (C4), 28.8 (*C4), 28.7 (C9″), 27.4 (*C9″), 15.5 (CH_3_), 15.2 (*CH_3_); C1″ resonance was not observed in the ^13^C NMR or HMBC spectra (*resonance of minor rotamer observed in the ^1^H NMR and/or ^13^C NMR spectra); IR (cm^−1^) $\bar{\nu }$_max_ 3280 (w), 2965 (w), 1772 (s, β-lactam C=O), 1704 (s), 1632 (w), 1532 (m), 1466 (w), 1441 (w), 1371 (m), 1232 (m), 1153 (w), 1109 (m), 1088 (m) 1000 (w), 829 (m), 765 (s), 627 (m), 520 (w); MS (ESI + ve) *m/z* 566 ([M + H]^+^, 100%), 588 ([M + Na]^+^, 70%); (ESI − ve) *m/z* 564 ([M − H]^−^, 26%), 600 ([M + Cl]^−^, 18%), 628 ([M + TFA − H]^−^, 12%); HRMS (ESI + ve TOF) calcd for C_27_H_27_N_5_O_5_S_2_ 588.1351, found 582.1357 ([M + Na]^+^.

(6*R*,7*S*)-7-Methoxy-8-oxo-7-(2-(4-phenyl-1*H*-1,2,3-triazol-1-yl)acetamido)-3-((phenylthio)methyl)-5-thia-1-azabicyclo[4.2.0]oct-2-ene-2-carboxylic acid **21h**

This compound was prepared according to the General Procedure using **20** (55.4 mg, 0.114 mmol), Pd_2_(dba)_3_·CHCl_3_ (11.8 mg, 0.0114 mmol, 10 mol%), BIPHEPHOS (18.8 mg, 0.0239 mmol, 21 mol%), and benzenethiol (23.3 μL, 0.227 mmol, 2.0 equiv.) in MeCN (2 mL) with 10 h of reaction time. Work-up and purification via RP-HPLC (MeCN/H_2_O—17:8–7:10, 15 min, injection volume = 160 μL) as described above gave the titled compound as a pale-yellow oil (12.7 mg, 0.0236 mmol, 21%). Compound purity by HPLC (see Supplementary Materials): 99.7%, 254 nm; ${\left[\alpha \right]}_{D}^{24}$ +42.4 (*c* 0.40, MeCN); ^1^H NMR (400 MHz, CD_3_CN) δ 8.15 (s, 1H, CH, triazole), 7.86 (d, *J* = 7.1 Hz, 1H, H2″), 7.78 (s, 1H, NH), 7.48–7.27 (m, 7H, H3″, H4″, H6″ and H7″), 5.26 (s, 2H, CH_2_-triazole), 4.95 (s, 1H, H6), 4.26 (d, *J* = 13.4 Hz, 1H, H3′_A_), 3.89 (d, *J* = 13.3 Hz, 1H, H3′_B_), 3.53 (d, 1H, *J* = 16.8 Hz, H4_A_), 3.51 (s, 3H, O-CH_3_), 3.36 (d, *J* = 16.8 Hz, 1H, H4_B_); ^13^C NMR (101 MHz, CD_3_CN) δ 167.0 (C=O, amide), 162.5 (C2′), 161.0 (C8), 147.8 (Cq, tetrazole), 136.4 (C3), 134.6 (C5″), 132.8 (C6″), 131.4 (C1″), 129.7 (C7″), 129.5 (C3″), 128.7 (C4″), 128.2 (C8″), 126.1 (C2″), 125.2 (C2), 122.9 (CH, triazole), 96.7 (C7), 65.6 (C6), 53.8 (O-CH_3_), 52.5 (CH_2_-triazole), 37.2 (C3′), 29.1 (C4); IR (cm^−1^) $\bar{\nu }$_max_ 3531 (w), 3280 (w), 3031 (w), 2943 (w), 2840 (w), 1772 (s, β-lactam C=O), 1704 (s), 1623 (w), 1533 (m), 1482 (w), 1468 (m), 1415 (m), 1371 (m), 1152 (w), 1109 (m), 1087 (m), 1024 (w), 1000 (2), 832 (s), 746 (s), 629 (s); MS (ESI + ve) *m/z* 538 ([M+H]^+^, 91%), 560 ([M + Na]^+^, 42%), 576 ([M + K]^+^ 37%); (ESI − ve) *m/z* 536 ([M − H]^−^, 19%), 650 ([M + TFA − H]^−^, 30%); HRMS (ESI + ve TOF) calcd for C_25_H_23_N_5_O_5_S_2_Na 560.1117, found 560.1122 ([M + Na]^+^.

## 4. Conclusions

The synthesis of eight 7α-methoxy-7-1*H*-1,2,3-triazol-1-ylacetamino-3′-arylthio-cephalosporic acid derivatives from 7-aminocephalosporic acid has been achieved. The synthesis avoids the use of toxic and potentially explosive diphenyldiazomethane and involves, for the first time, the synthesis of the 4-methoxybenzyl ester of (6*R*,7*S*)-3-[(acetyloxy)methyl]-7-amino-7-methoxy-8-oxo-5-thia-1-azabicyclo[4.2.0]oct-2-ene-2-carboxylic acid. The 7-(4-phenyl-1*H*-1,2,3-triazol-1-yl)acetamino moiety was introduced through azidation of the novel α-bromo acetamide **18** followed by a Cu(I)-catalysed azide–alkyne cycloaddition reaction with phenylacetylene, while the 3′-arylthiol substituent was introduced via a palladium-catalyzed arylthioallylation reaction. The chemistry described, and several of the synthetic intermediates reported here, are potentially valuable methods and scaffolds, respectively, for further development of β-lactam antibiotics.

## Figures and Tables

**Table 1:** Attempts toward the synthesis of thiol **21a** from triazole **20**.

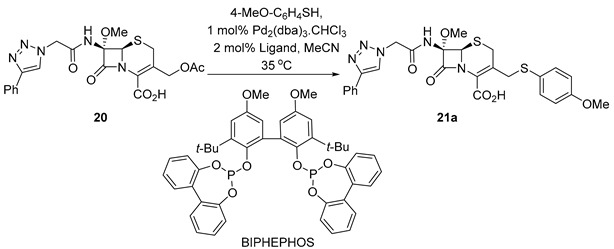					
**Entry**	**Ligand**	**Ligand Equiv.**	**Pd Equiv.**	**Reaction Time**	**Ratio of 20:21a ^a^**
1	BIPHEPHOS	2 mol%	2 mol%	5 d	1:0
2	P(OPh)_3_	2 mol%	2 mol%	5 d	1:0
3	dppf	2 mol%	2 mol%	5 d	1:0
4 ^b^	BIPHEPHOS	2 mol%	2 mol%	12 h	1:2.3
5 ^c^	BIPHEPHOS	20 mol%	21 mol%	10 h	0:1

^a^ Determined using ^1^H NMR analysis on the crude reaction mixture; ^b^ Reaction was performed under sonication; ^c^ 2.0 equiv. of 4-methoxybenzenethiol was used.

## Data Availability

The data presented in this study are available in Supplementary Materials.

## Supplementary Materials

The following supporting information can be downloaded at: https://www.mdpi.com/article/10.3390/molecules28217338/s1, copies of ^1^H and ^13^C NMR spectra and HPLC traces.
