# Supplementary material for: Synthesis of 7α-Methoxy-7-(4-phenyl-1H-1,2,3-triazol-1-yl)acetamino-3′-arylthio-cephalosporic Acid Derivatives from 7-Aminocephalosporic Acid
Source: Molecules. 2023 Oct 30;28(21):7338. doi: 10.3390/molecules28217338 (PMC10650751; doi:10.3390/molecules28217338)
Supplement: Supplementary file 1 [file molecules-28-07338-s001.zip › molecules-2685614-supplementary.pdf]

Supporting Information

Synthesis of 7α-methoxy-7-(4-phenyl-1*H*-1,2,3-triazol-1-yl)acetamino-3'-arylthio-cephalosporic acid derivatives from 7-aminocephalosporic acid

Wendy Y. Cun, Paul A. Keller and Stephen G. Pyne *

School of Chemistry and Molecular Bioscience, Molecular Horizons Research Institute, University of Wollongong, Wollongong, NSW 2522, Australia;

ywc538@uowmail.edu.au (W.Y.C.); keller@uow.edu.au (P.A.K.)

* Correspondence: spyne@uow.edu.au

Copies of ^{1}H and ^{13}C NMR spectra Pages 2-21

Copies of HPLC traces for **21a**–**21h** Pages 22-29

^{1}H and ^{13}C NMR Spectra

Compound **12** (^{1}H NMR [500 MHz] spectrum, CD_3COCD_3)

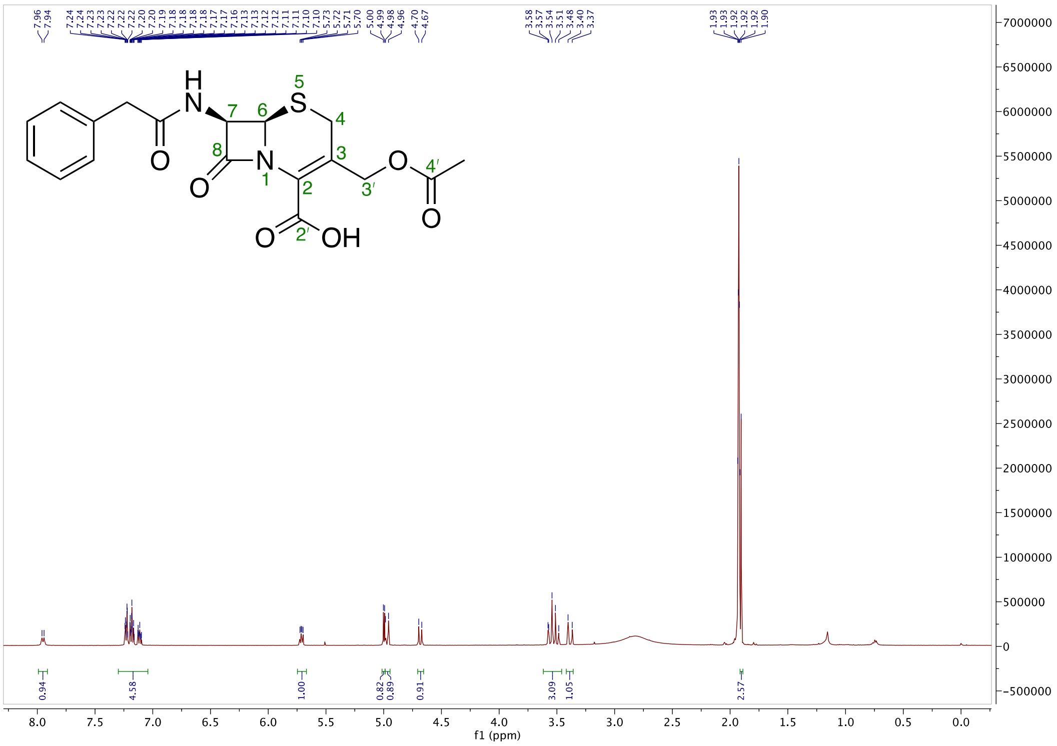

Benzhydryl 2,2,2-trichloroacetimidate (^{1}H NMR [500 MHz] spectrum, $CDCl_3$)

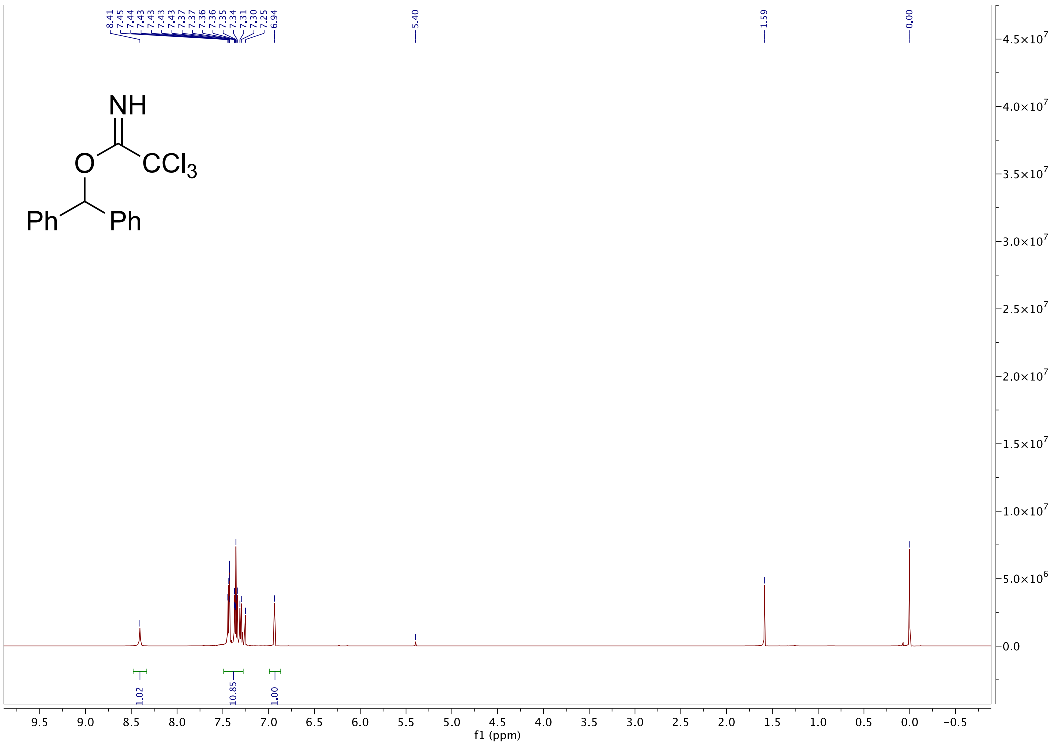

Compound **13** (^1H NMR [500 MHz] spectrum, $CDCl_3$)

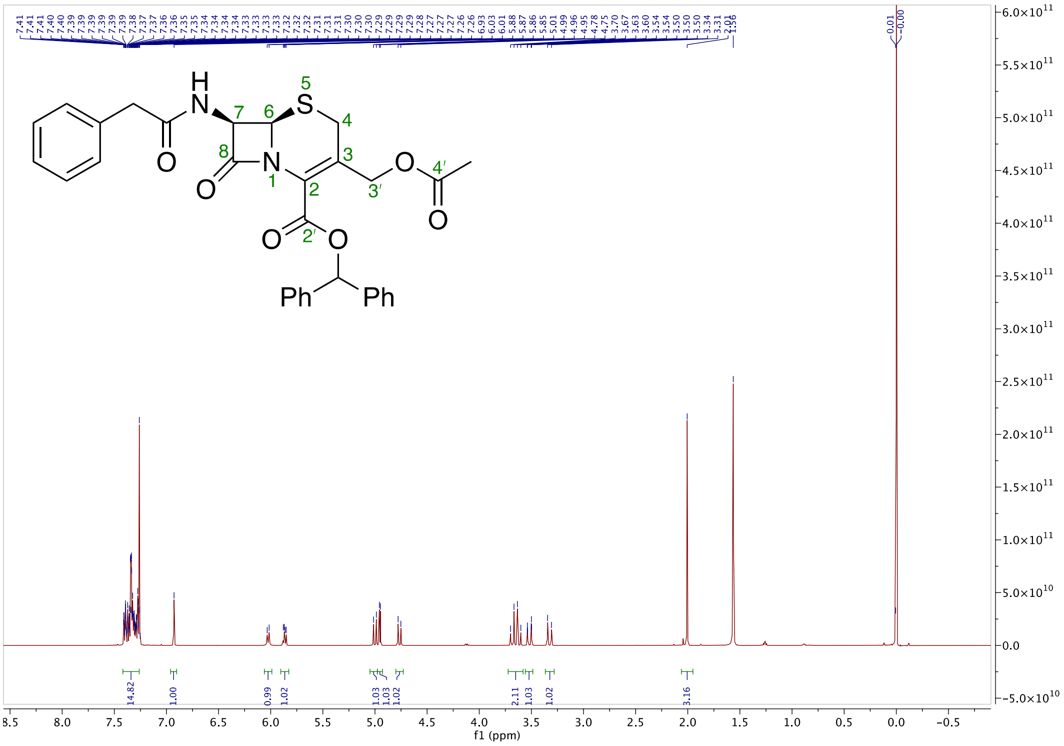

Compound **14** (yellow) (^1H NMR [500 MHz] spectrum, CD_3SOCD_3)

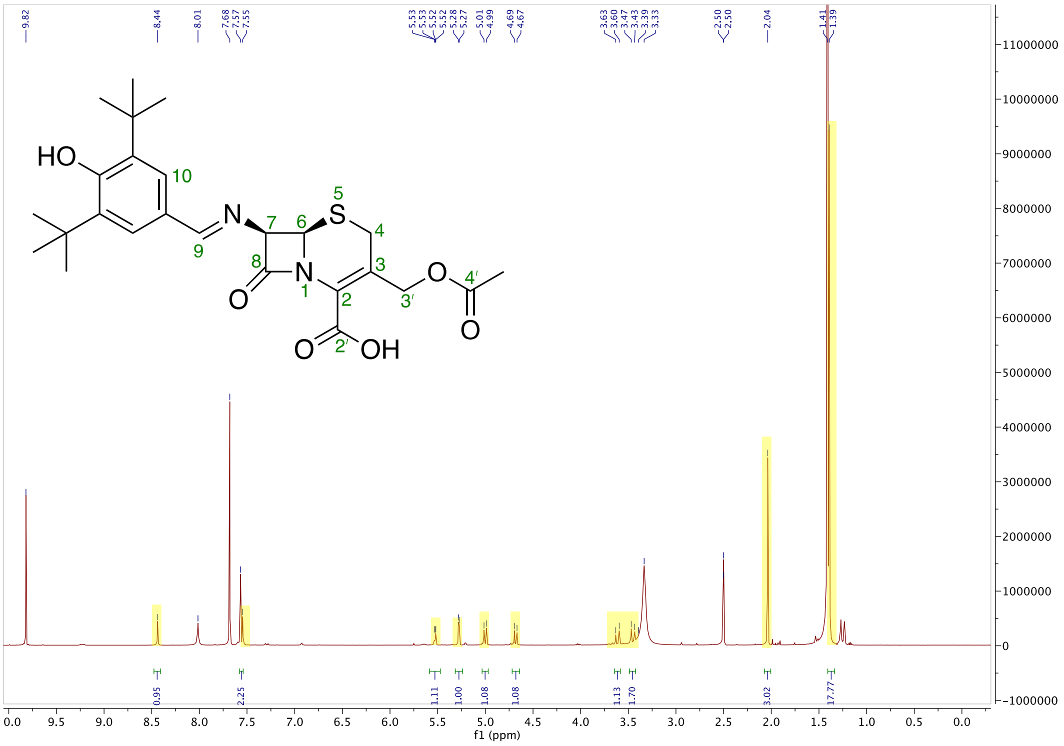

Compound **15a** (yellow) (^{1}H NMR [500 MHz] spectrum, CD_3OD)

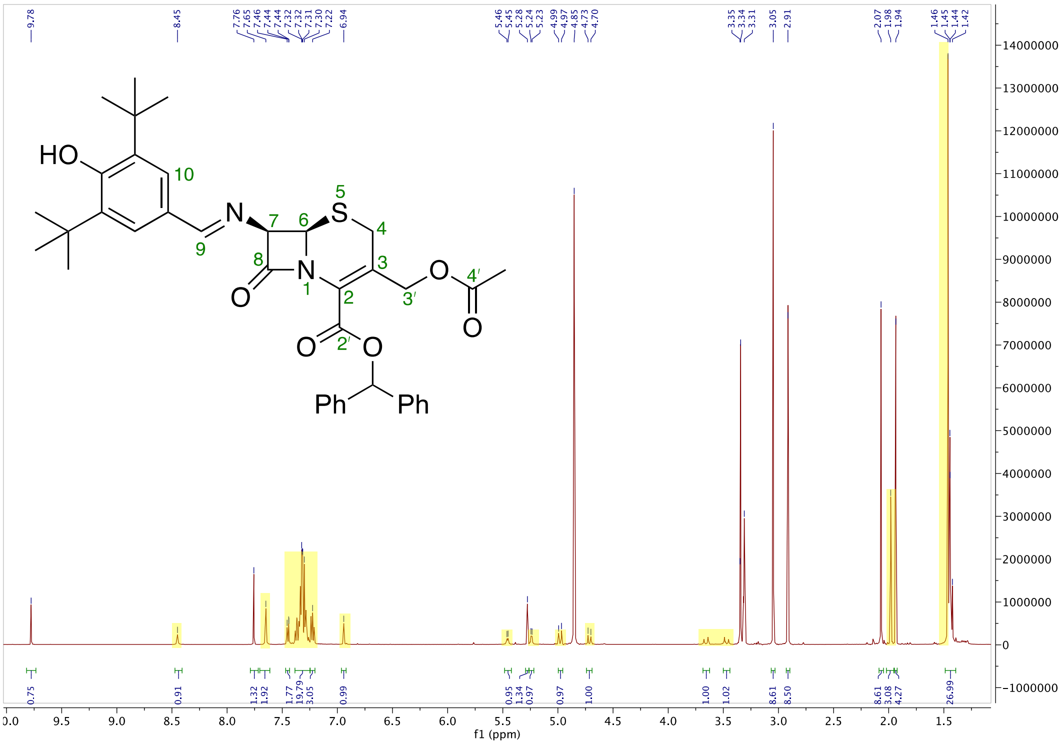

Compound **16a** (^{1}H NMR [500 MHz] spectrum, $CDCl_3$)

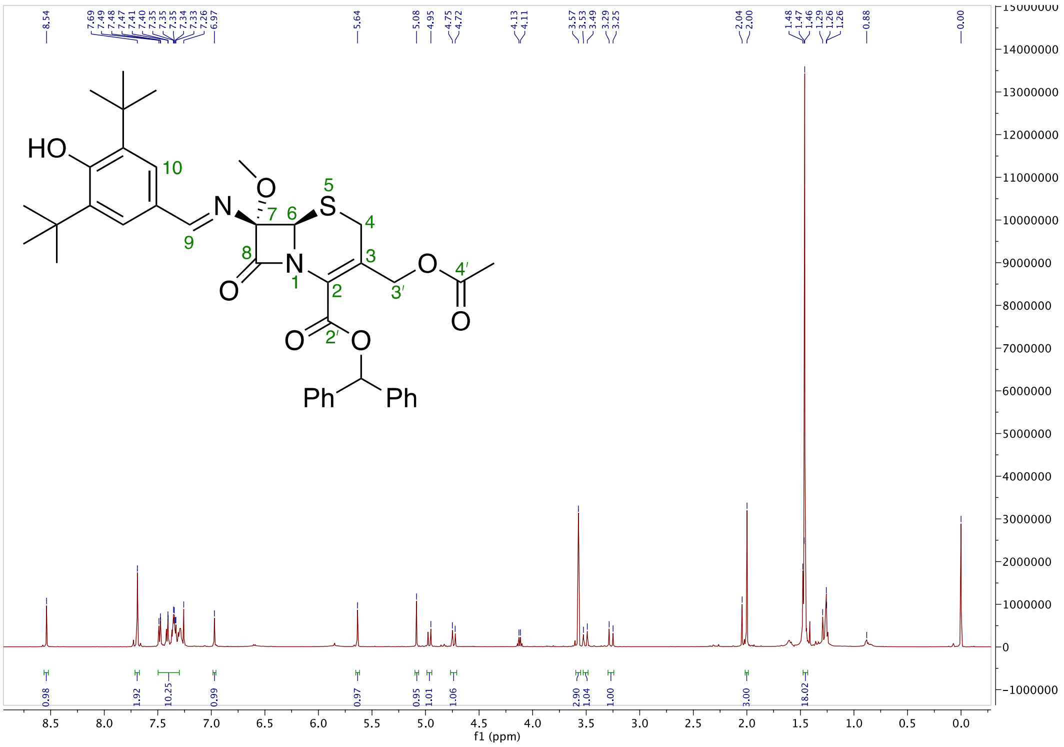

Compound **7** (yellow) in mixture with by-product (pink) (^{1}H NMR [500 MHz] spectrum, $CDCl_3$)

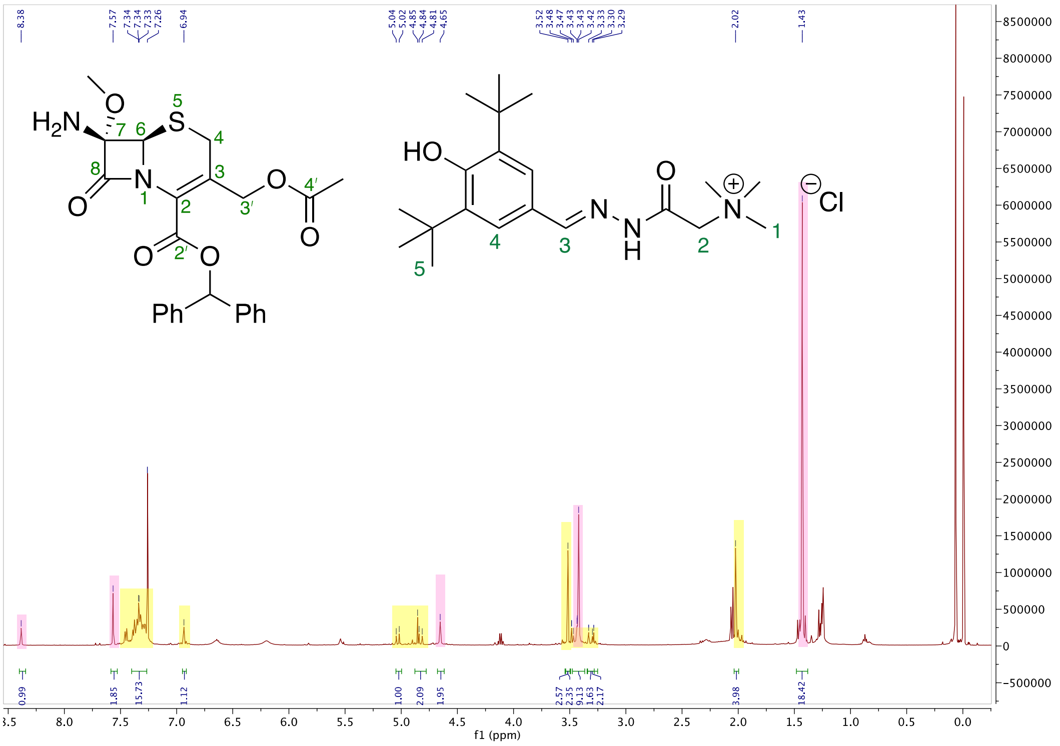

Compound **8** (^{1}H NMR [500 MHz] and DEPT [126 Hz] spectra $CDCl_3$)

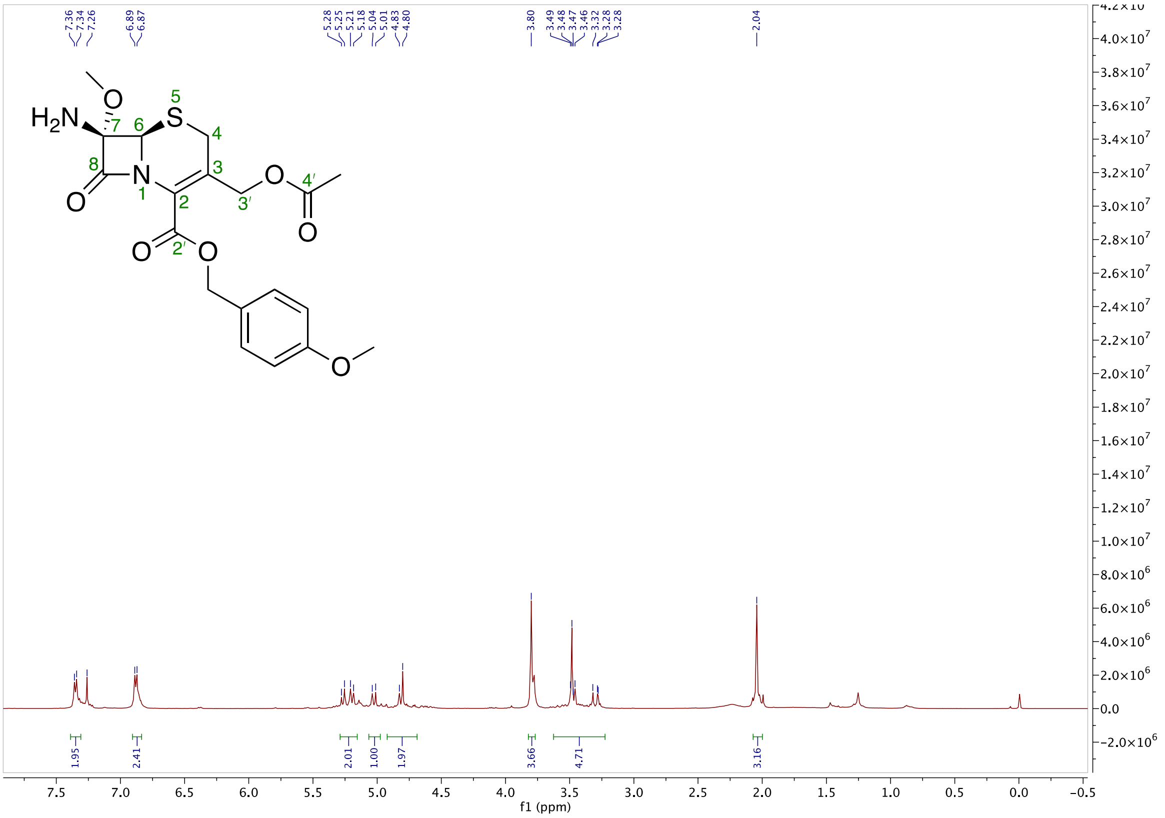

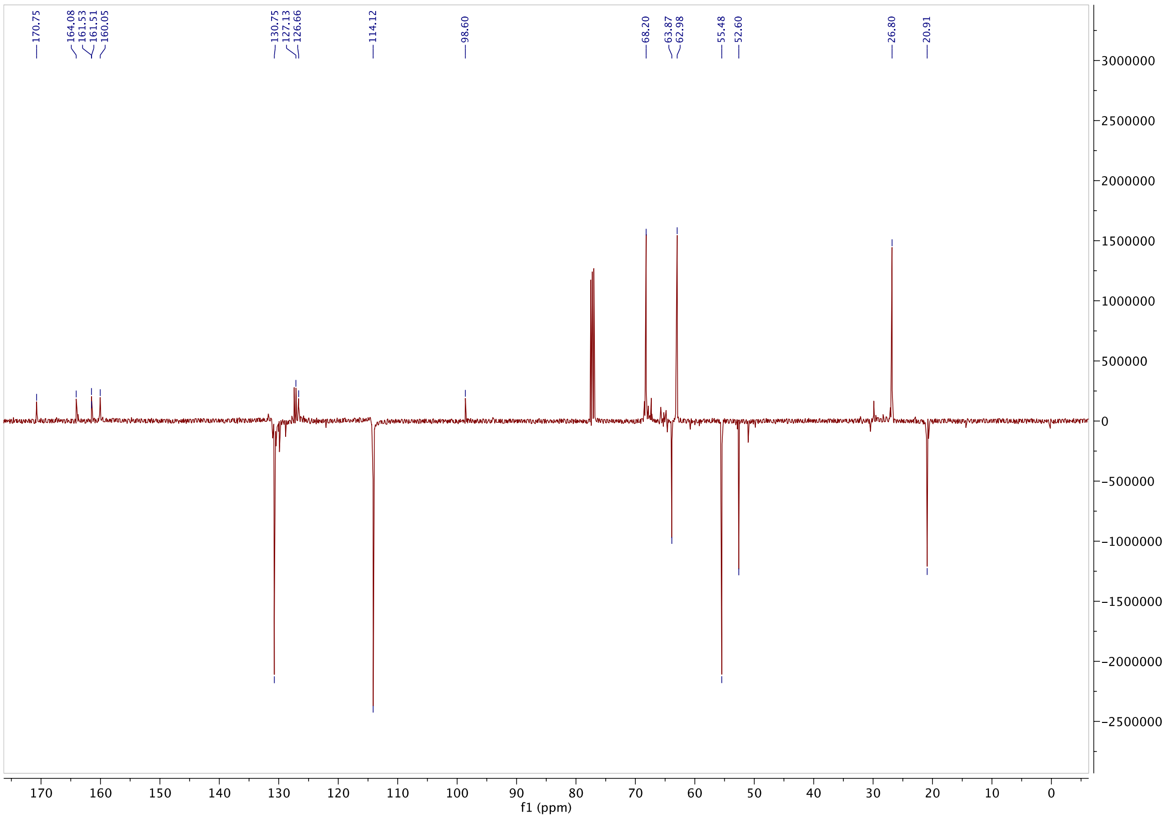

Compound **17a** (^{1}H NMR [400 MHz] and DEPT [101 Hz] spectra $CDCl_3$)

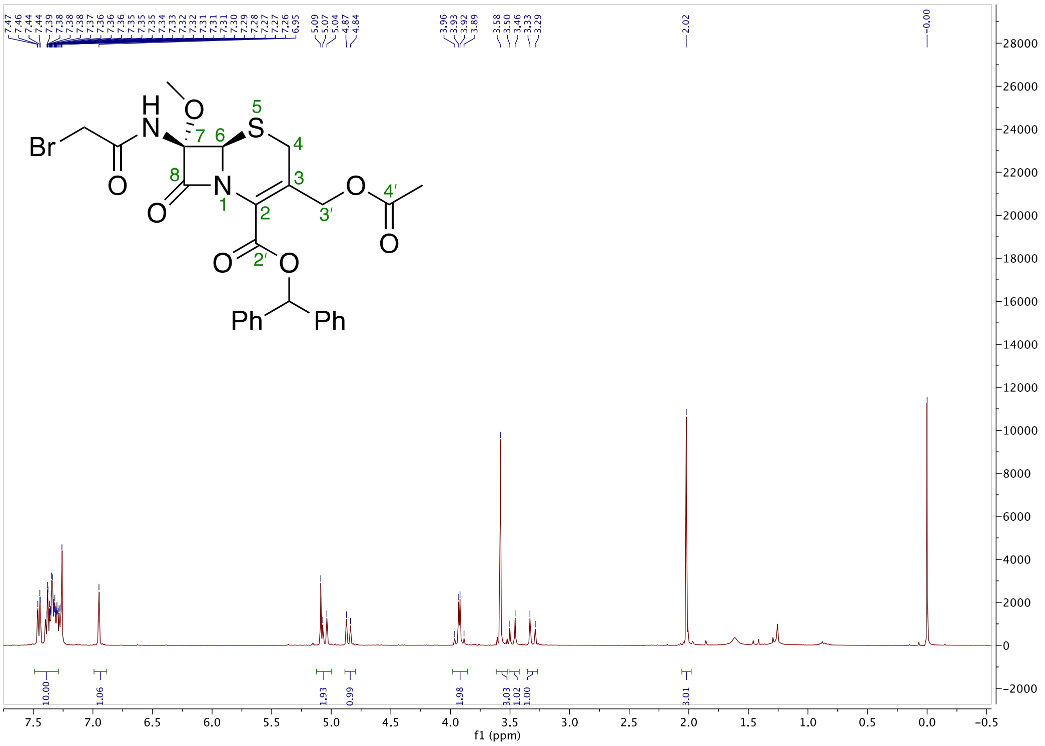

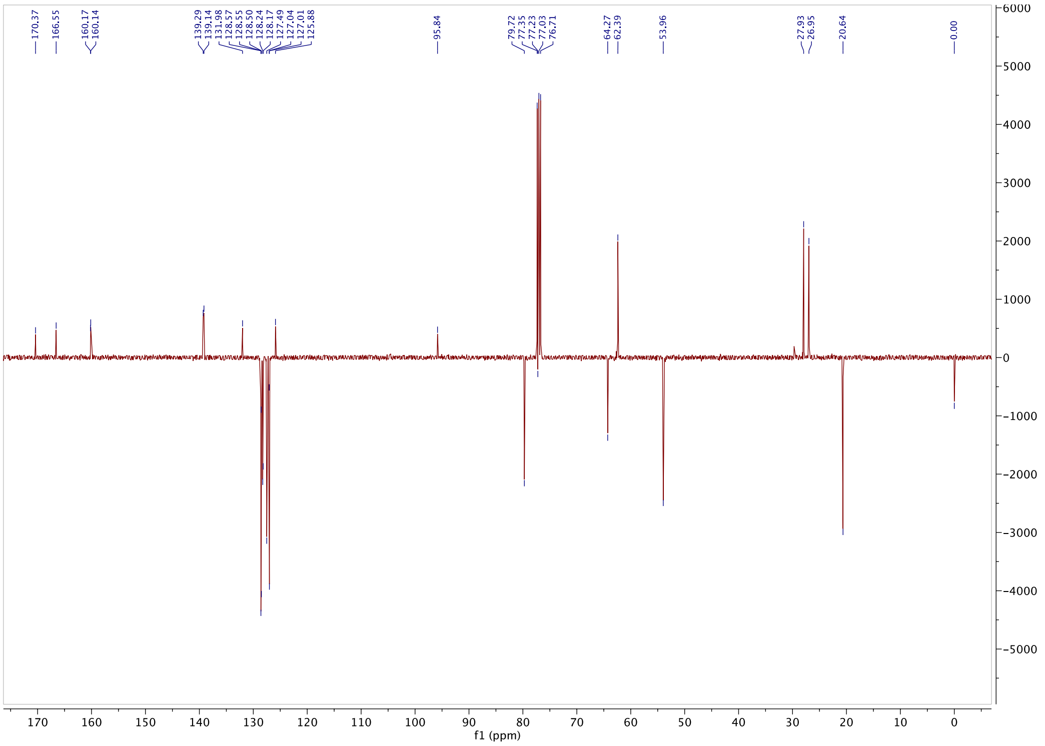

Compound **15b** (yellow) (^{1}H NMR [500 MHz] spectrum, CD_3OD)

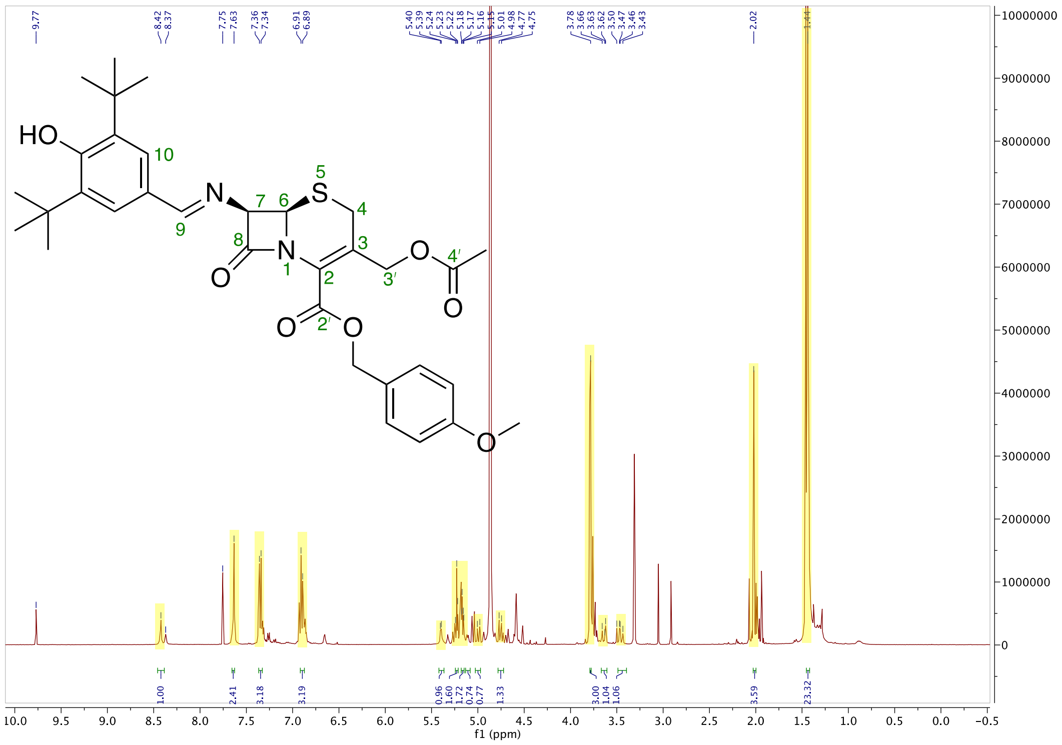

Compound **16b** (^{1}H NMR [400 MHz] and DEPT [101 Hz] spectra $CDCl_3$)

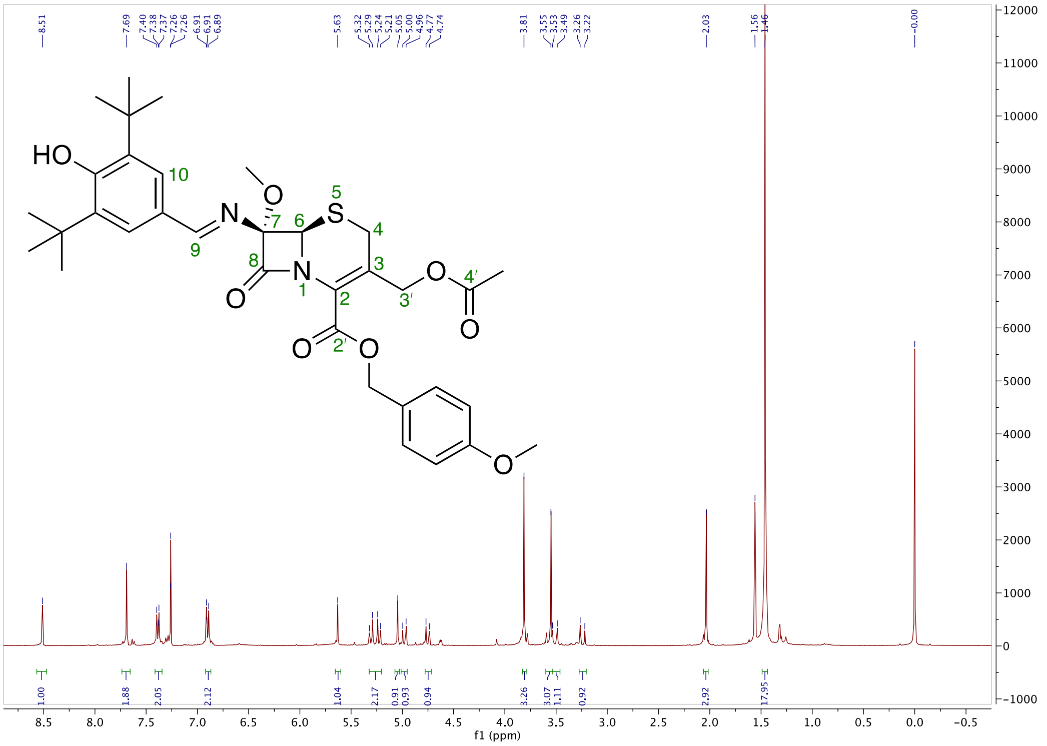

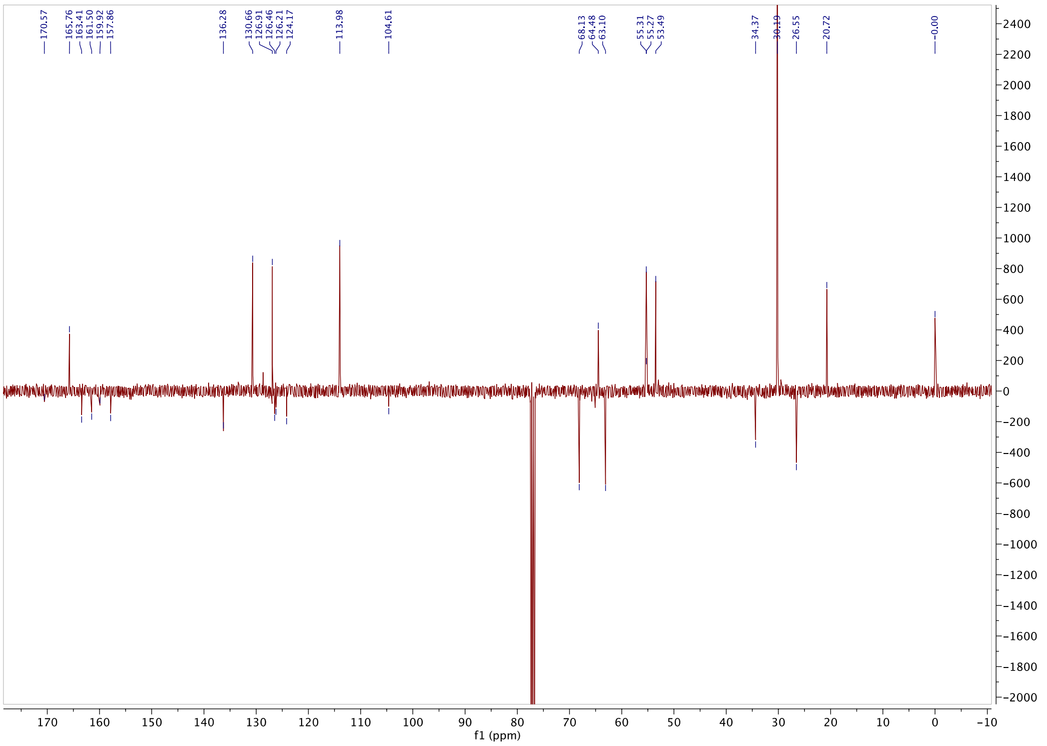

Compound **17b** (^{1}H NMR [400 MHz] and DEPT [101 Hz] spectra $CDCl_3$)

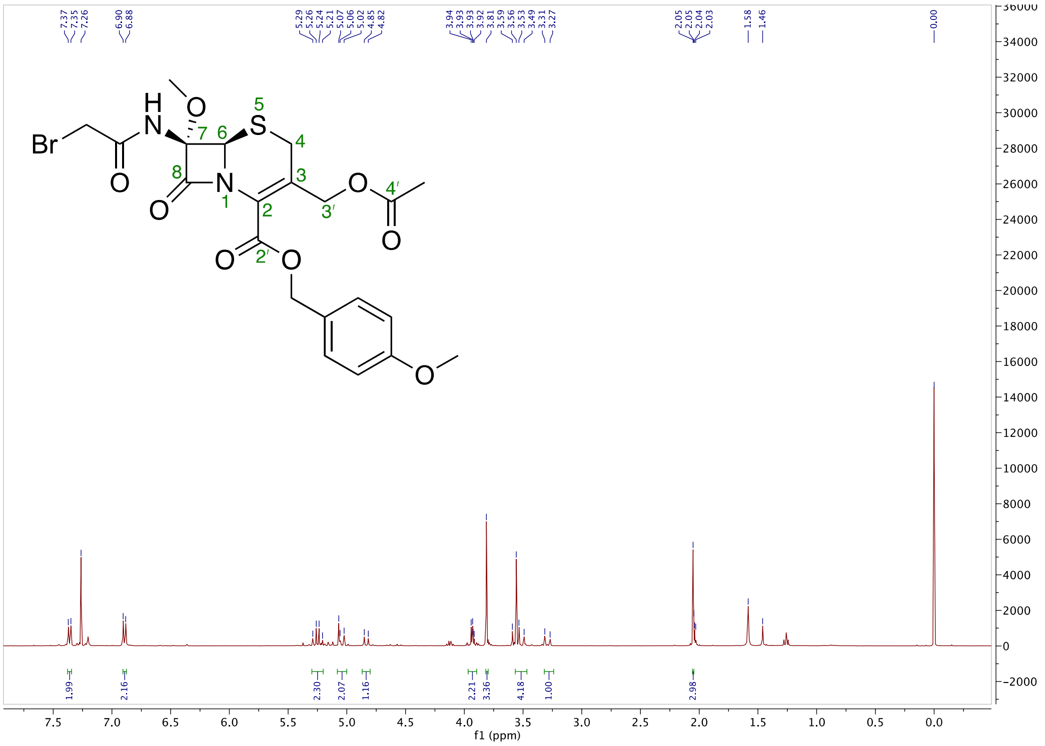

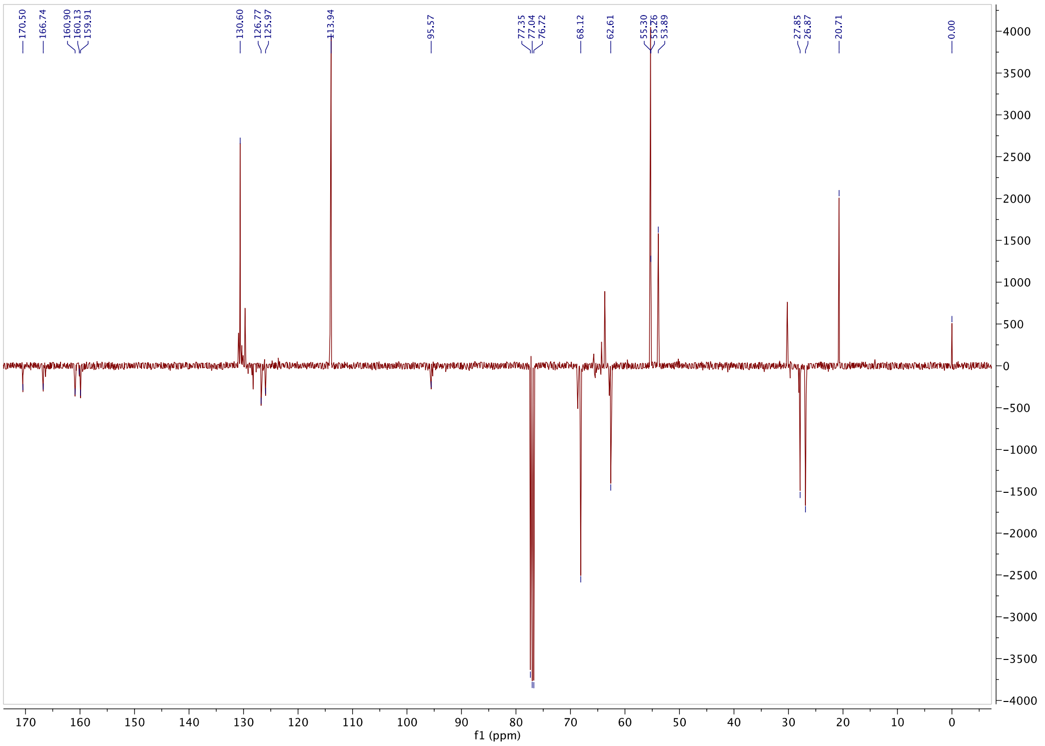

Compound **18** (^{1}H NMR [400 MHz] and DEPT [101 Hz] spectra CD_3CN)

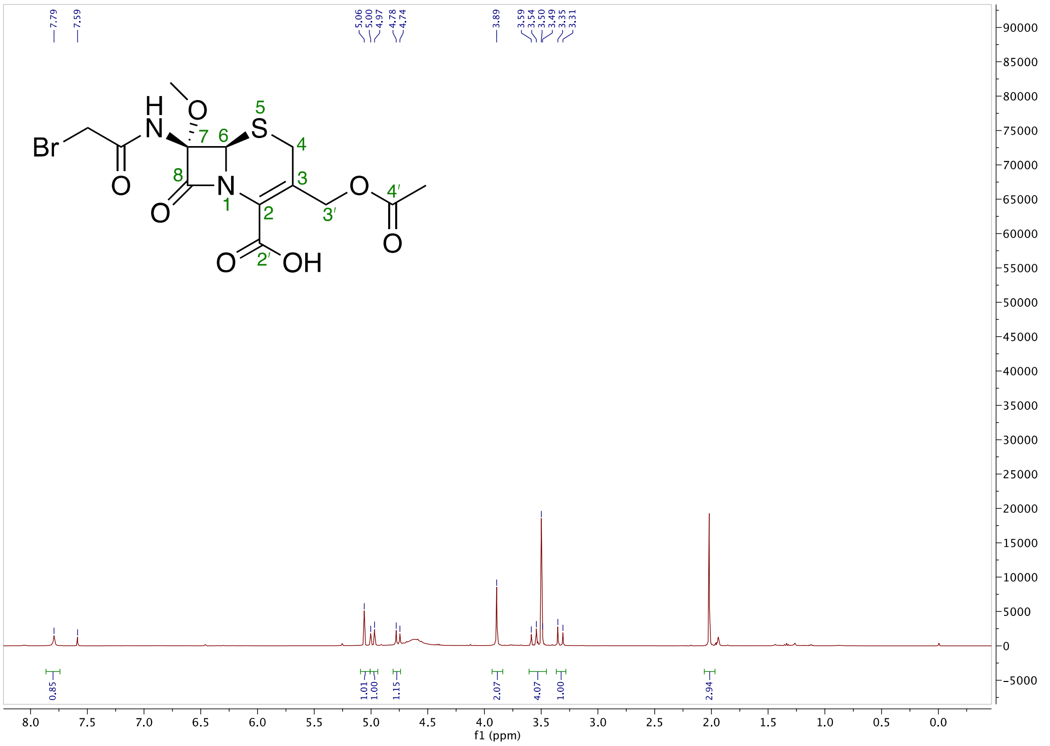

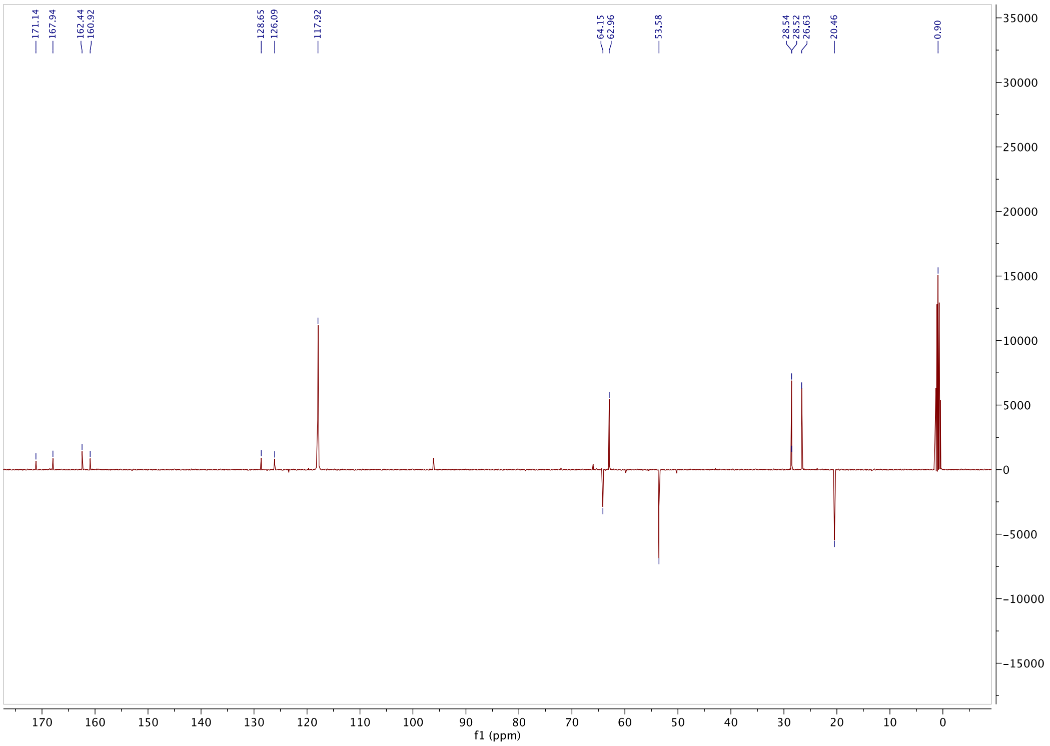

Compound **19** (^{1}H NMR [400 MHz] and DEPT [101 Hz] spectra CD_3CN)

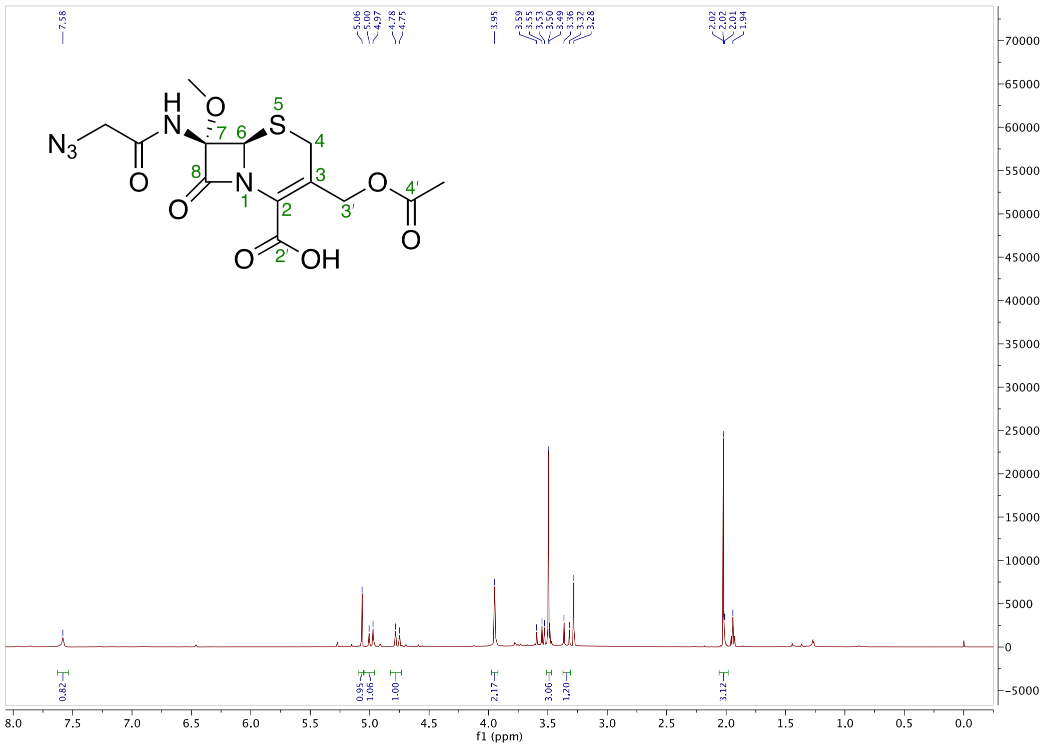

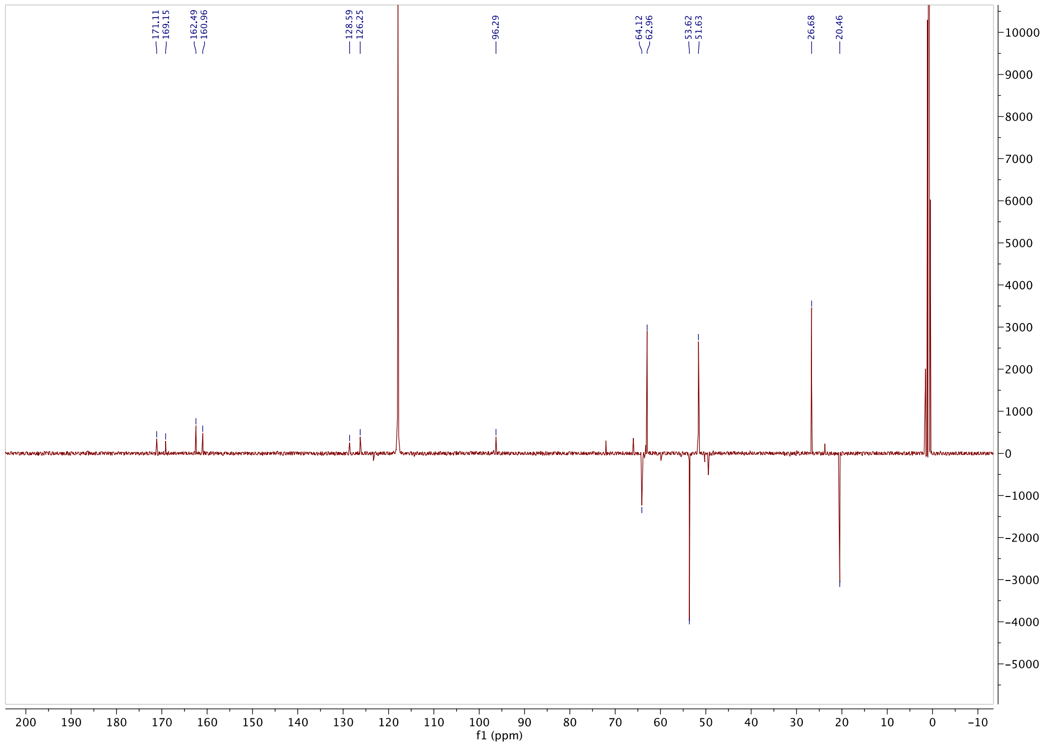

Compound **20** (^{1}H NMR [400 MHz] and DEPT [101 Hz] spectra CD_3CN)

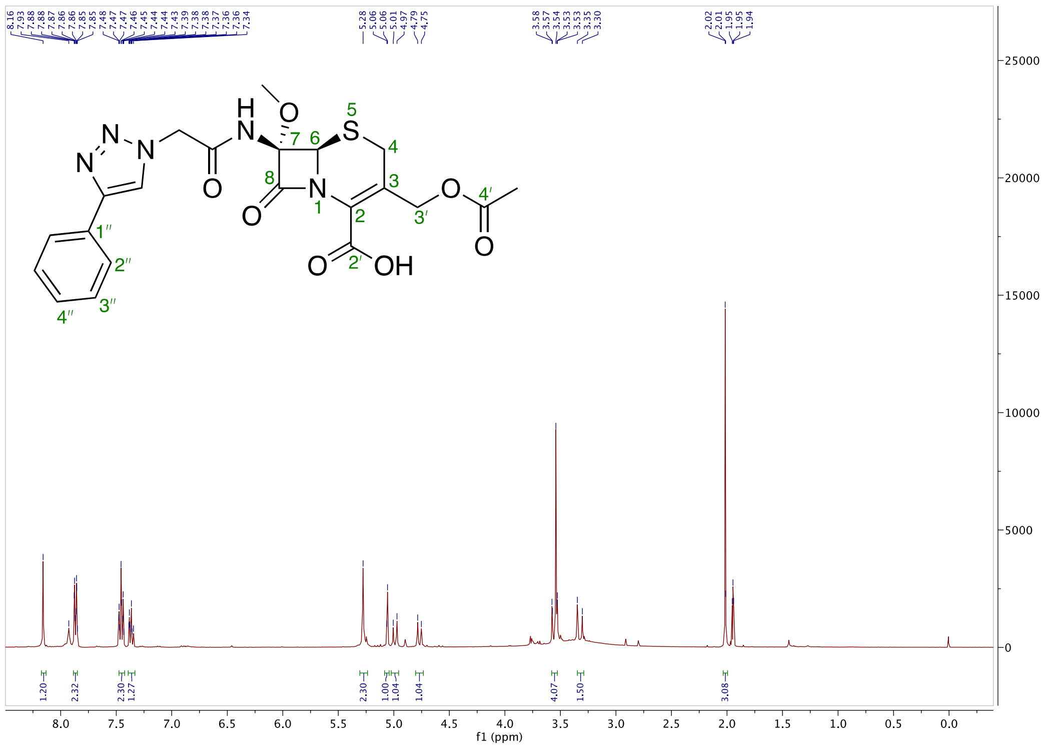

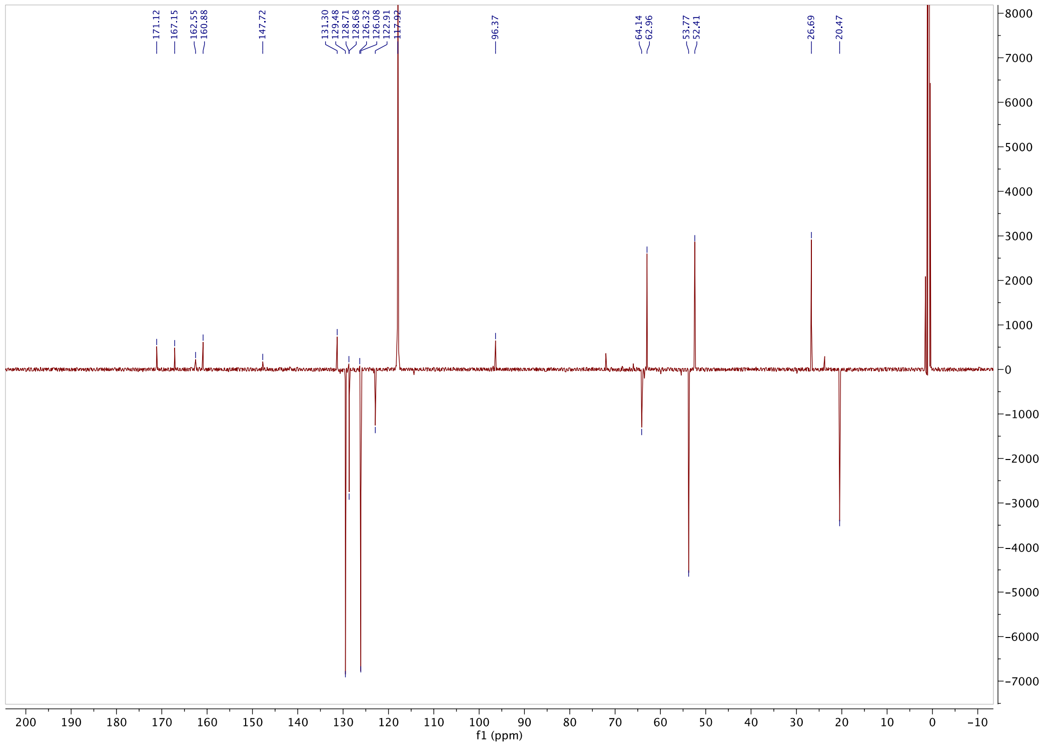

Compound **21a** (^{1}H NMR [400 MHz] and DEPT [101 Hz] spectra, CD_3CN)

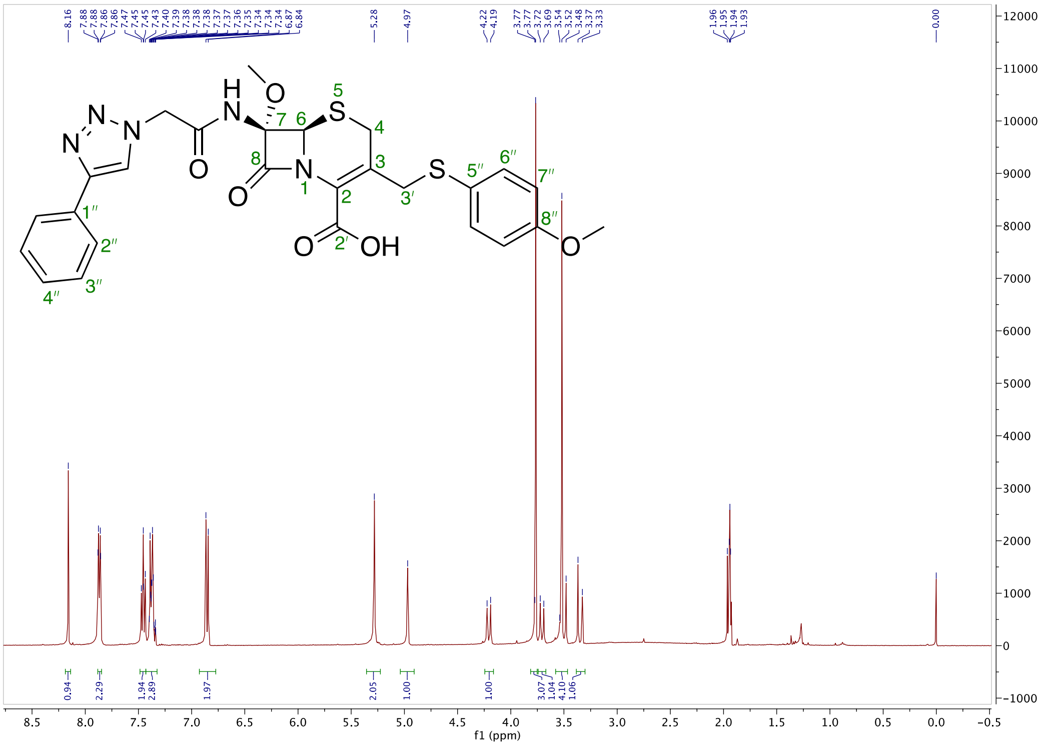

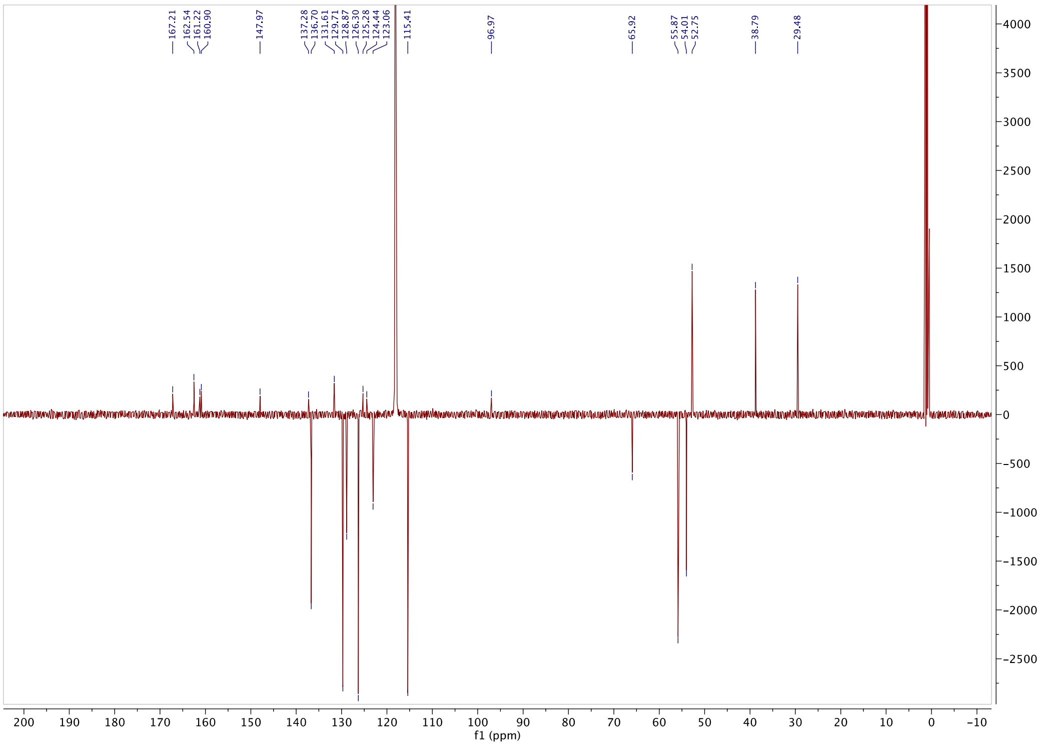

Compound **21b** (^{1}H NMR [400 MHz] and DEPT [101 Hz] spectra, CD_3CN)

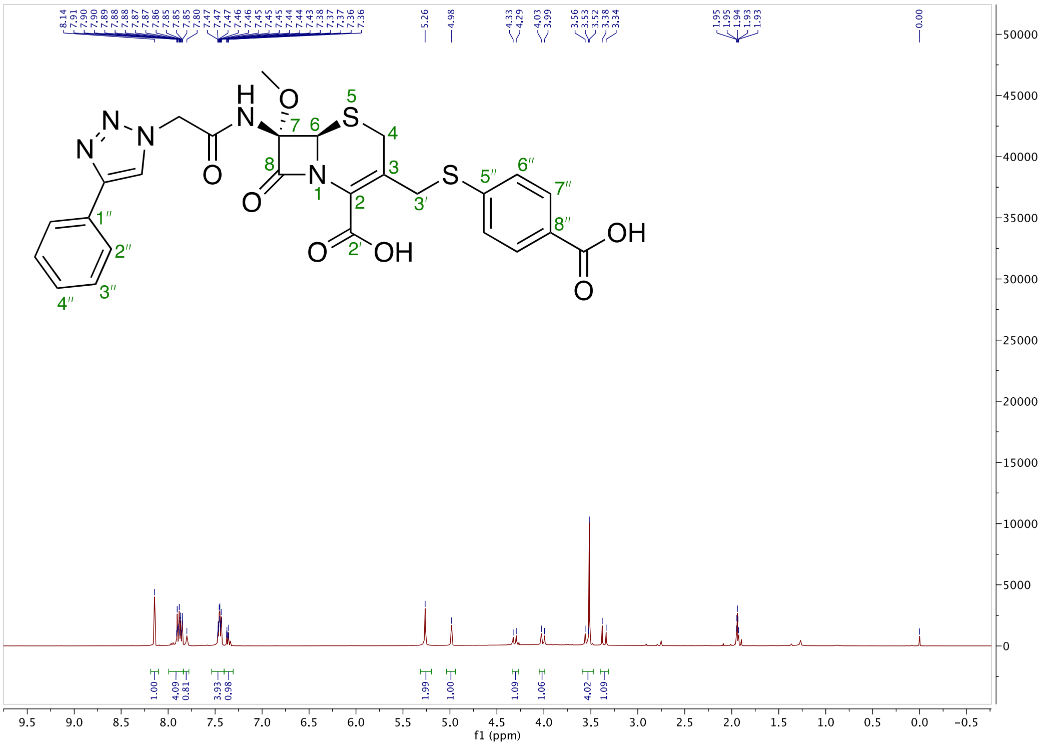

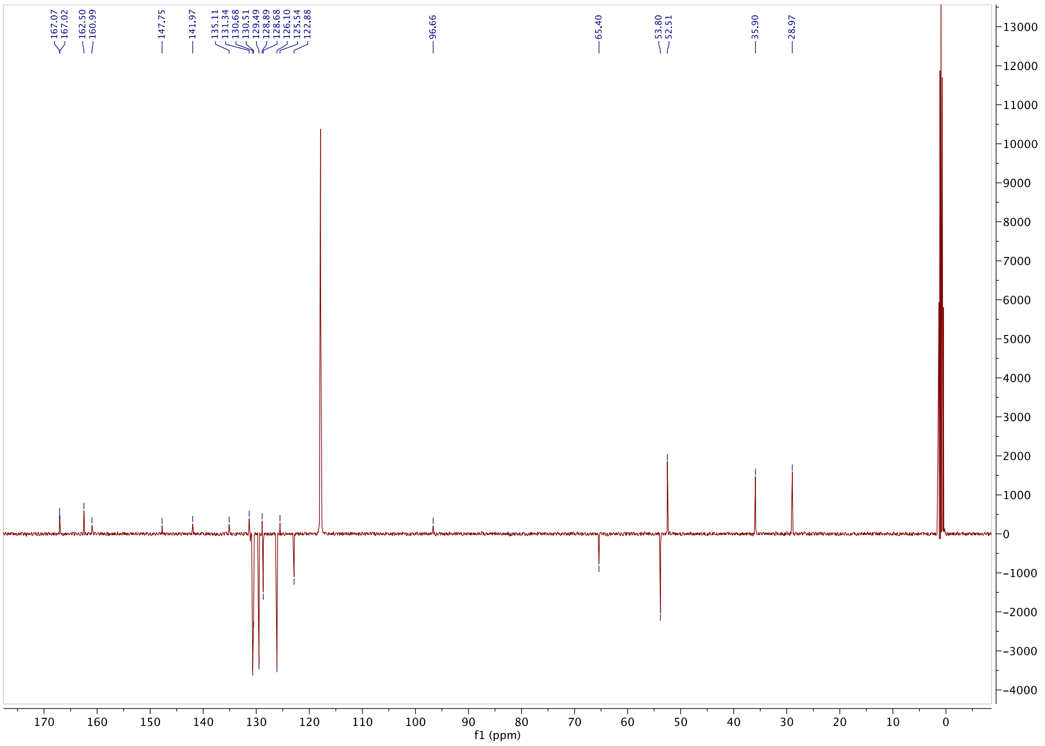

Compound **21c** (^{1}H NMR [400 MHz] and DEPT [101 Hz] spectra, CD_3CN)

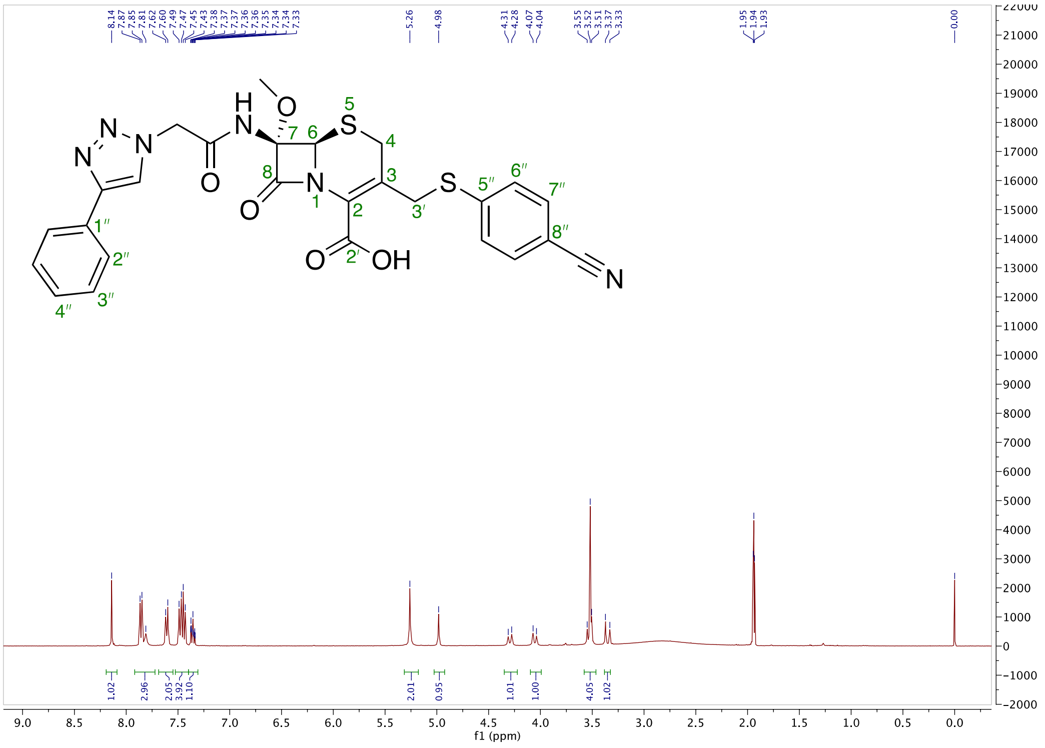

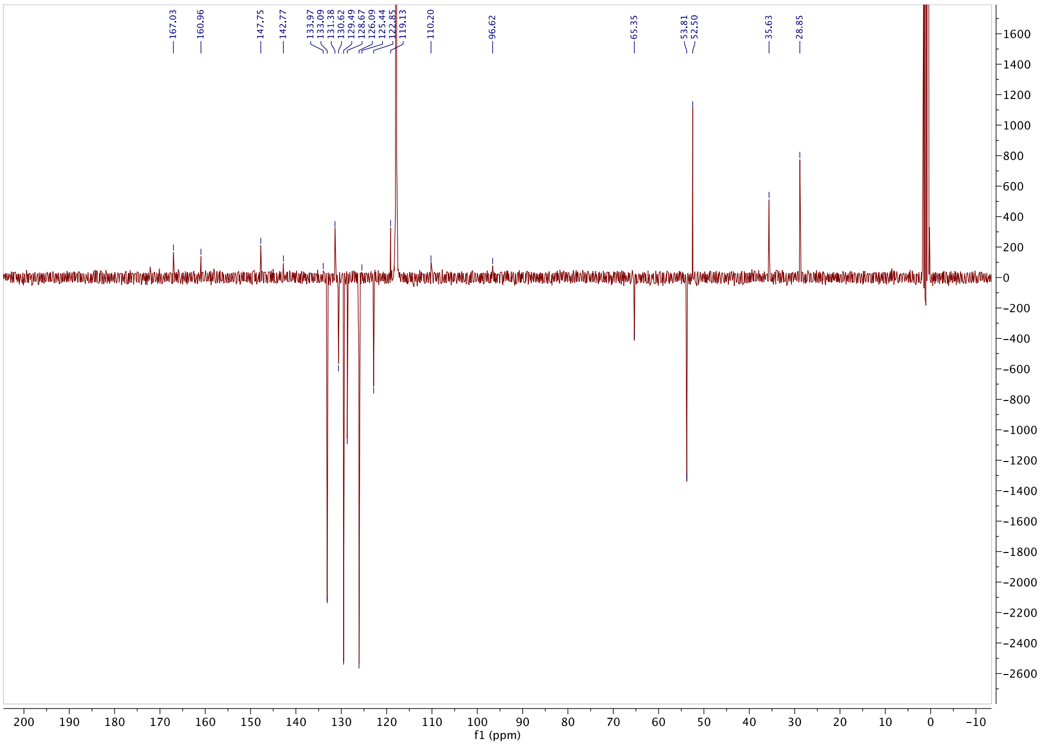

Compound **21d** (^{1}H NMR [400 MHz] and DEPT [101 Hz] spectra, CD_3CN)

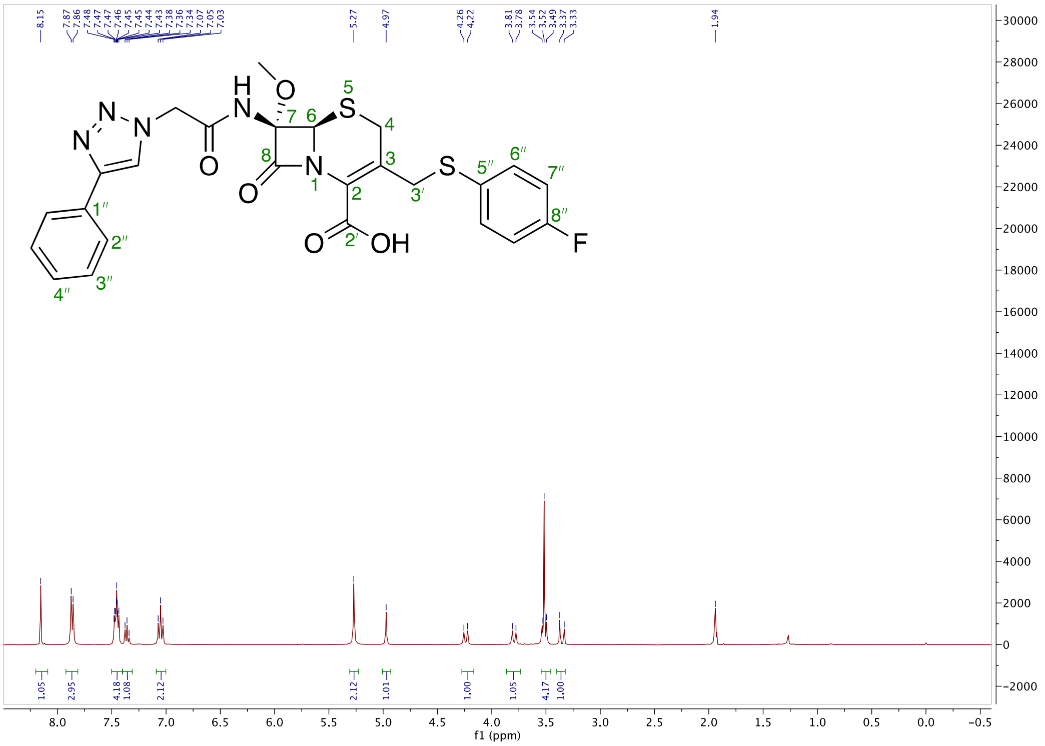

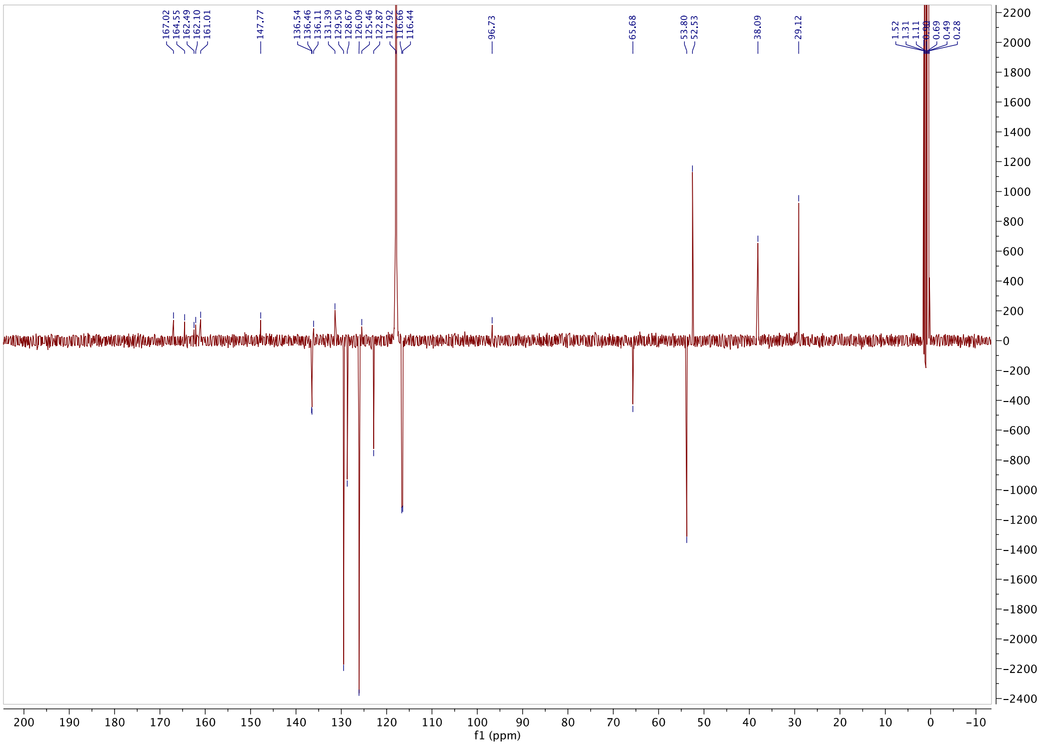

Compound **21e** (^{1}H NMR [400 MHz] and DEPT [101 Hz] spectra, CD_3CN)

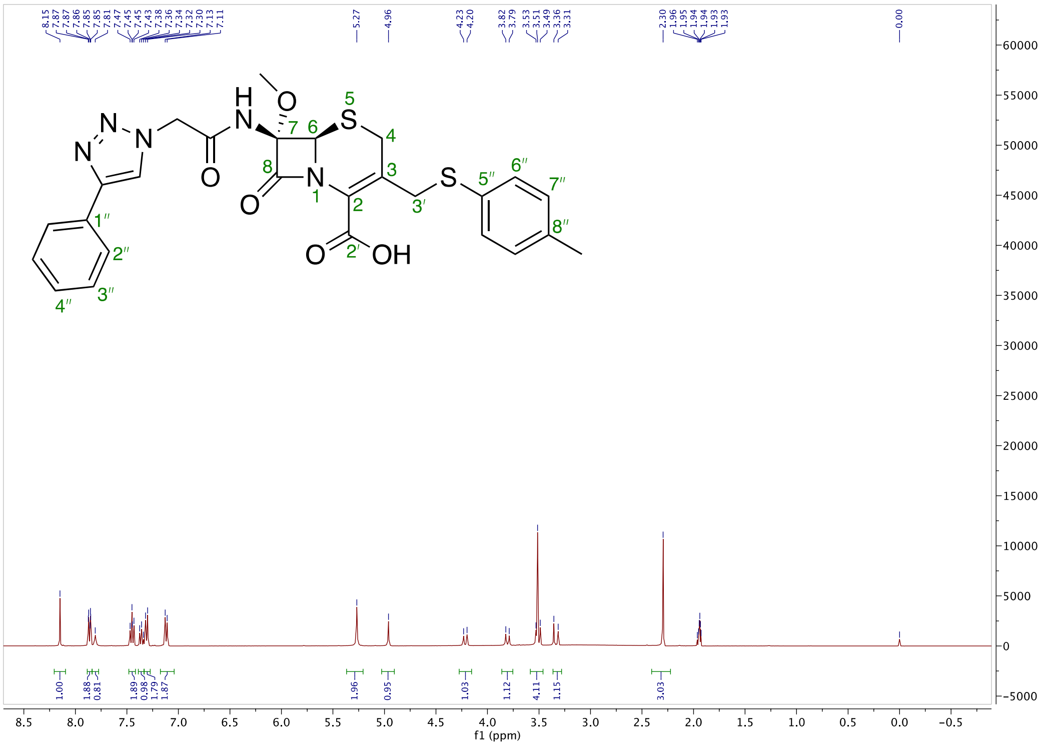

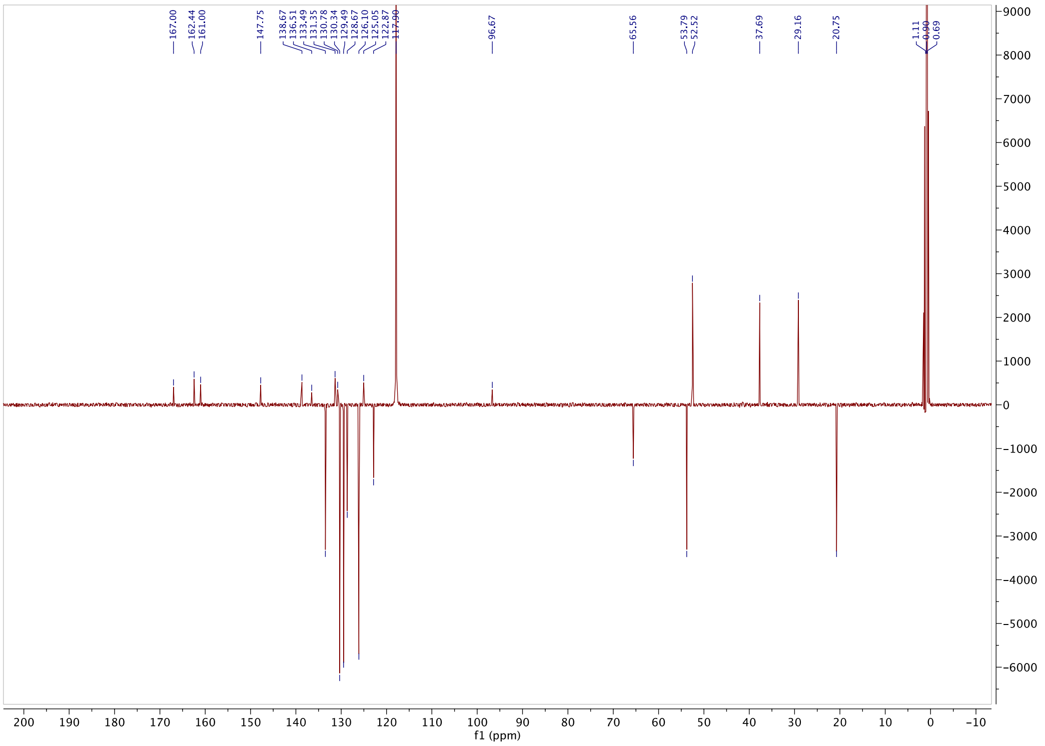

Compound **21f** (^{1}H NMR [400 MHz] and DEPT [101 Hz] spectra, CD_3CN)

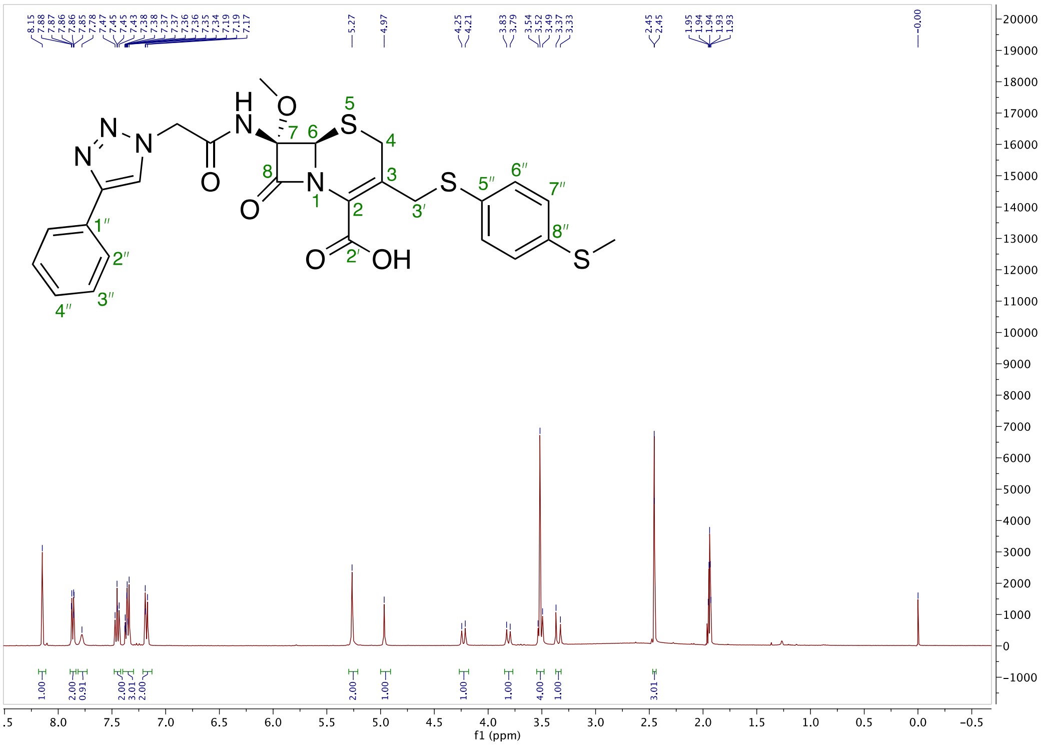

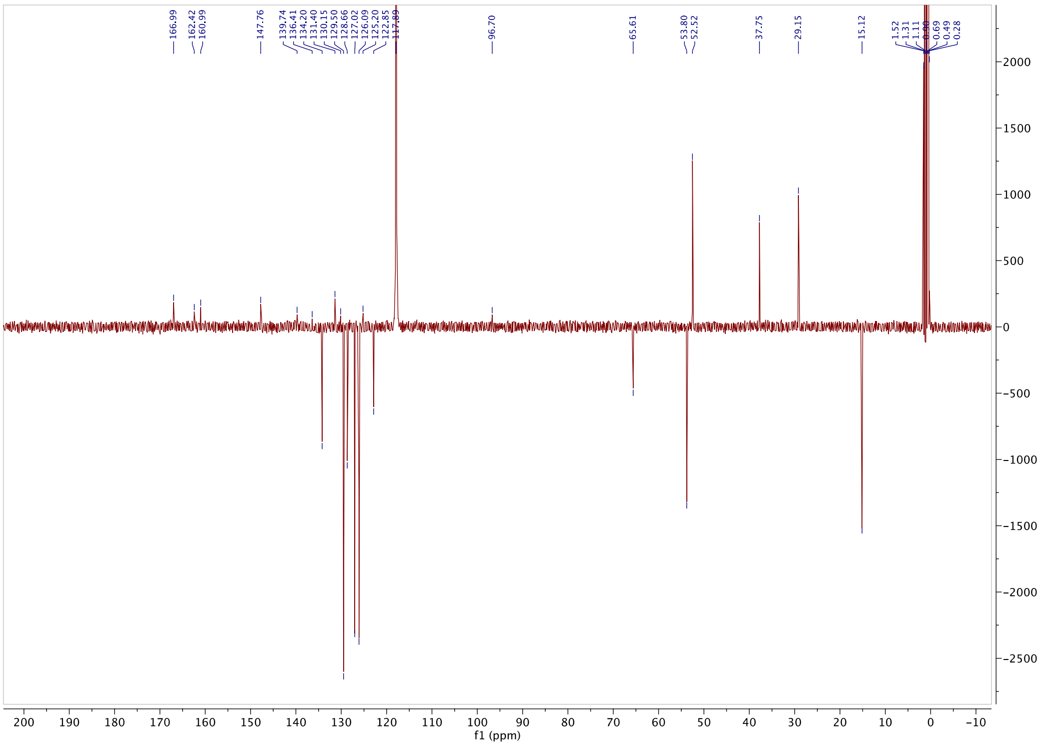

Compound **21g** (^{1}H NMR [400 MHz] and DEPT [101 Hz] spectra, CD_3CN)

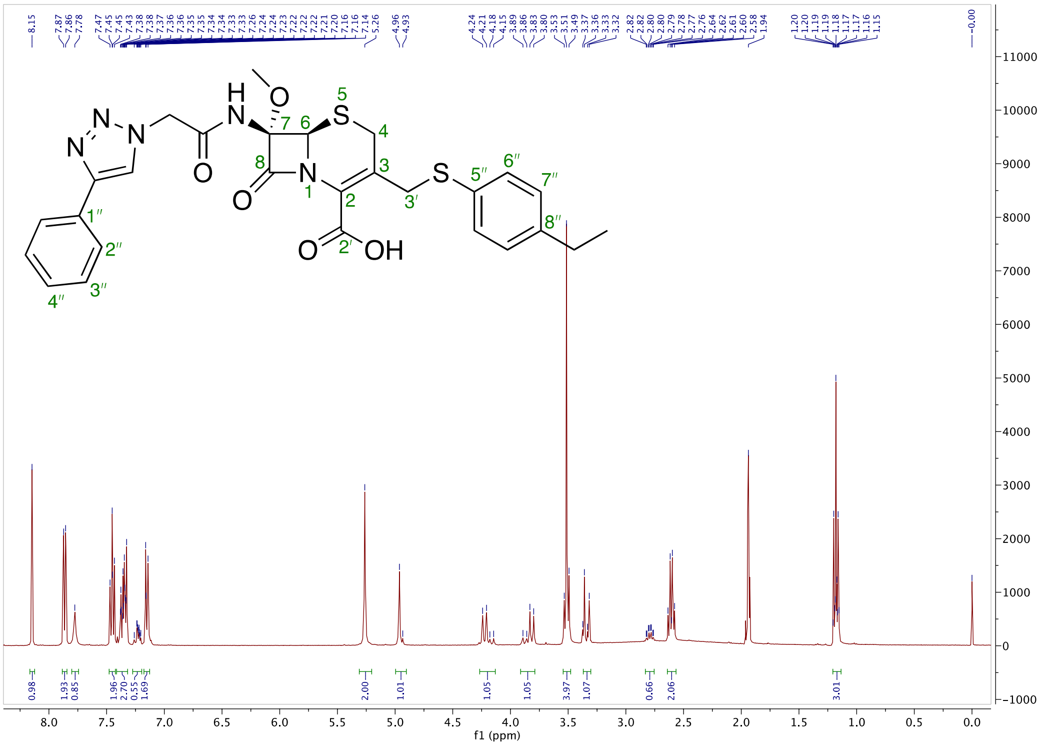

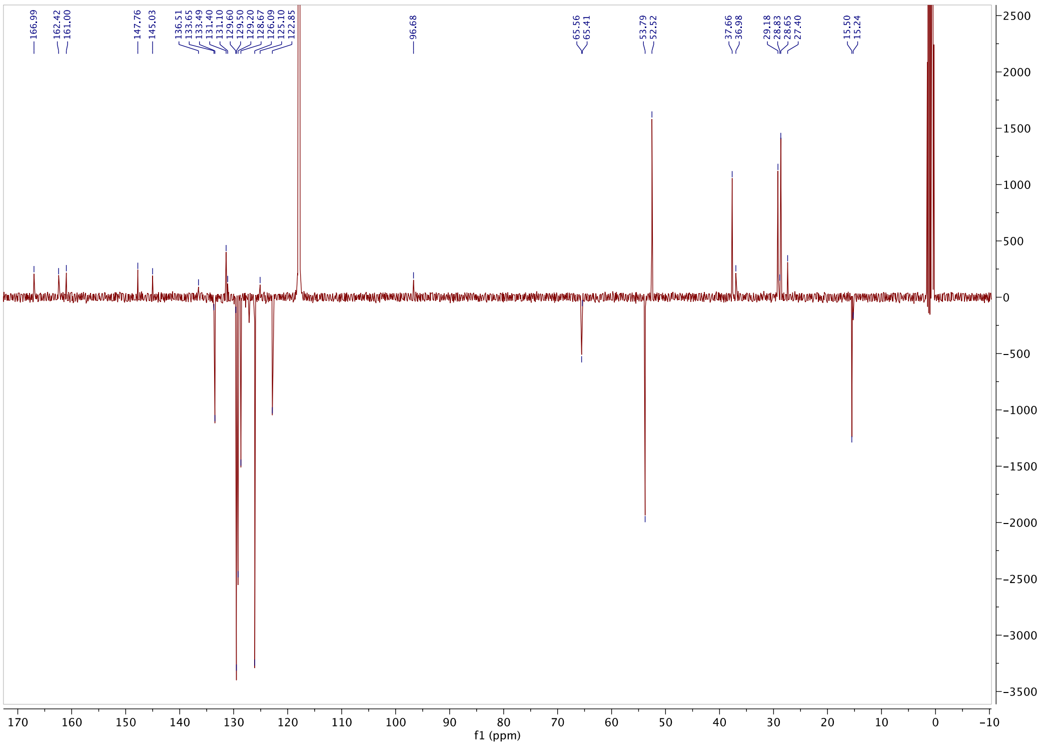

Compound **21h** (^{1}H NMR [400 MHz] and DEPT [101 Hz] spectra, CD_3CN)

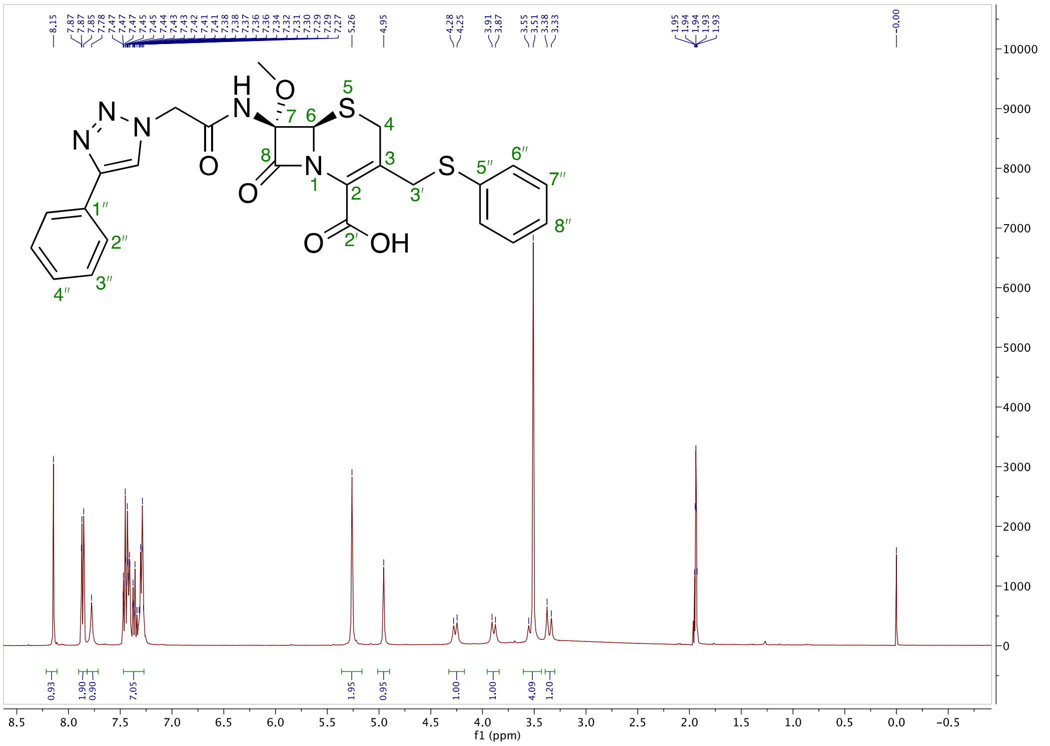

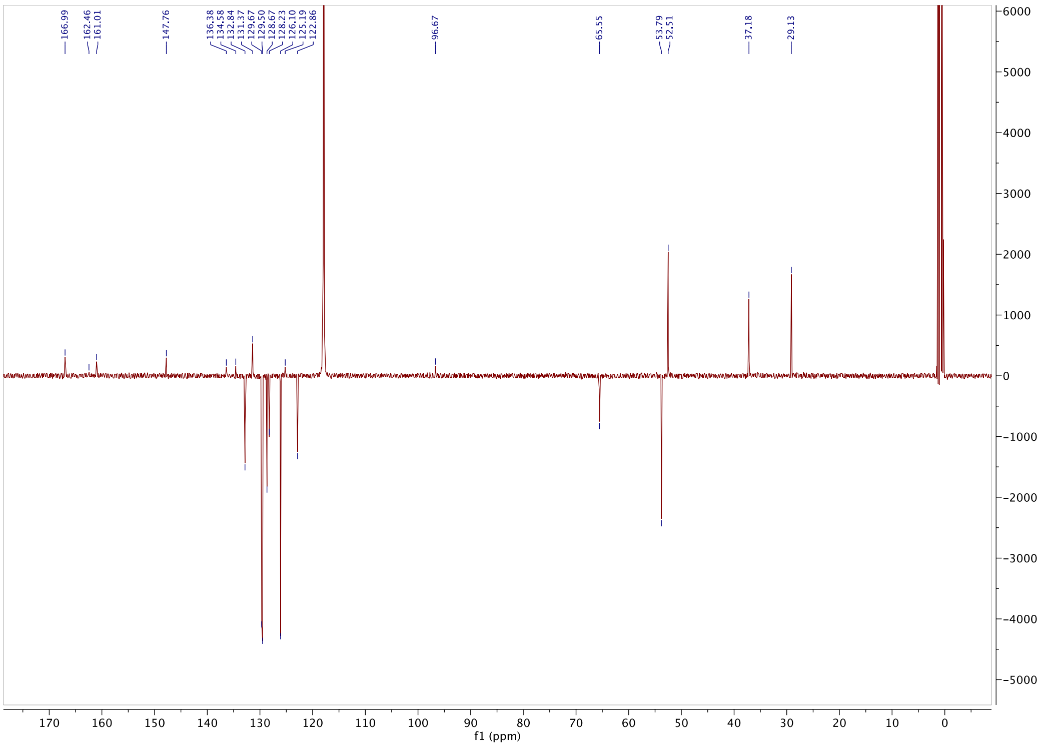

HPLC purity traces of 21a–h

Compound **21a**

Analysis Report

<Sample Information>

Sample Name	: ywc5-066		
Sample ID	: ywc5-066		
Data Filename	: ywc5-067.lcd		
Method Filename	: Purity check 10min.lcm		
Batch Filename	:		
Vial #	: 1-1	Sample Type	: Unknown
Injection Volume	: 5 uL		
Date Acquired	: 29/10/2022 11:21:56 AM	Acquired by	: System Administrator
Date Processed	: 29/10/2022 11:36:51 AM	Processed by	: System Administrator

<Chromatogram>

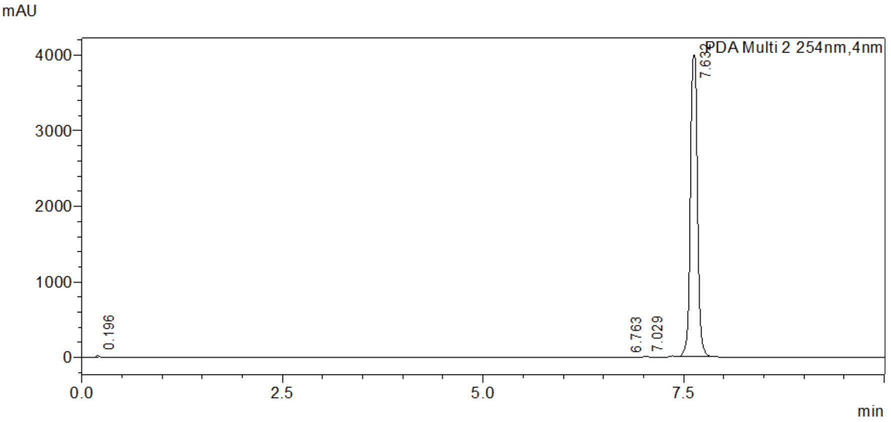

<Peak Table>

PDA Ch2 254nm

Peak#	Ret. Time	Peak Start	Peak End	Area	Height	Area%	Height%
1	0.196	0.160	0.224	49036	26968	0.198	0.671
2	6.763	6.688	6.816	6550	1594	0.026	0.040
3	7.029	7.008	7.061	3763	2101	0.015	0.052
4	7.632	7.413	7.925	24733856	3990169	99.761	99.237
Total				24793204	4020832	100.000	100.000

C:\LabSolutions\Data\Pyne\Wendy\ywc5-067.lcd

Compound **21b**

29/10/2022 11:58:49 AM Page 1 / 1

Analysis Report

<Sample Information>

Sample Name	: ywc5-068		
Sample ID	: ywc5-068		
Data Filename	: ywc5-068.lcd		
Method Filename	: Purity check 10min.lcm		
Batch Filename	:		
Vial #	: 1-1	Sample Type	: Unknown
Injection Volume	: 10 uL		
Date Acquired	: 29/10/2022 11:42:33 AM	Acquired by	: System Administrator
Date Processed	: 29/10/2022 11:56:48 AM	Processed by	: System Administrator

<Chromatogram>

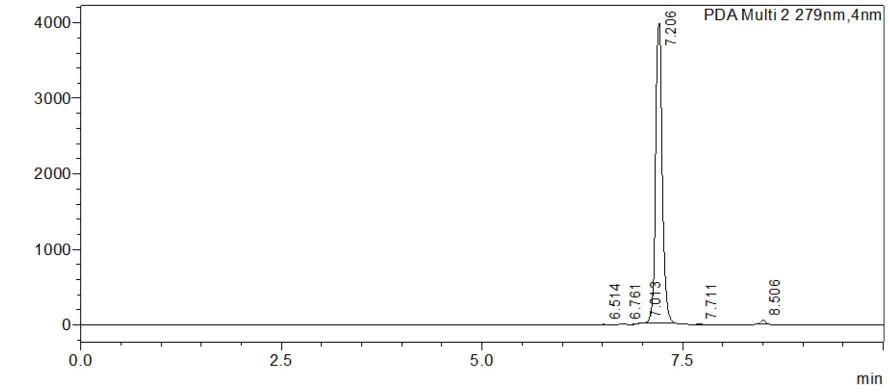

<Peak Table>

PDA Ch2 279nm

Peak#	Ret. Time	Peak Start	Peak End	Area	Height	Area%	Height%
1	6.514	6.475	6.571	13316	4286	0.057	0.106
2	6.761	6.720	6.805	19477	6459	0.084	0.160
3	7.013	6.848	7.019	2294	479	0.010	0.012
4	7.206	7.029	7.413	22992852	3969711	98.675	98.266
5	7.711	7.659	7.765	26705	7664	0.115	0.190
6	8.506	8.416	8.587	247065	51160	1.060	1.266
Total				23301708	4039759	100.000	100.000

C:\LabSolutions\Data\Pyne\Wendy\ywc5-068.lcd

Compound **21c**

Analysis Report

<Sample Information>

Sample Name	: ywc5-069		
Sample ID	: ywc5-069		
Data Filename	: ywc5-069.lcd		
Method Filename	: Purity check 10min.lcm		
Batch Filename	:		
Vial #	: 1-1	Sample Type	: Unknown
Injection Volume	: 10 uL		
Date Acquired	: 29/10/2022 12:00:14 PM	Acquired by	: System Administrator
Date Processed	: 29/10/2022 12:10:15 PM	Processed by	: System Administrator

<Chromatogram>

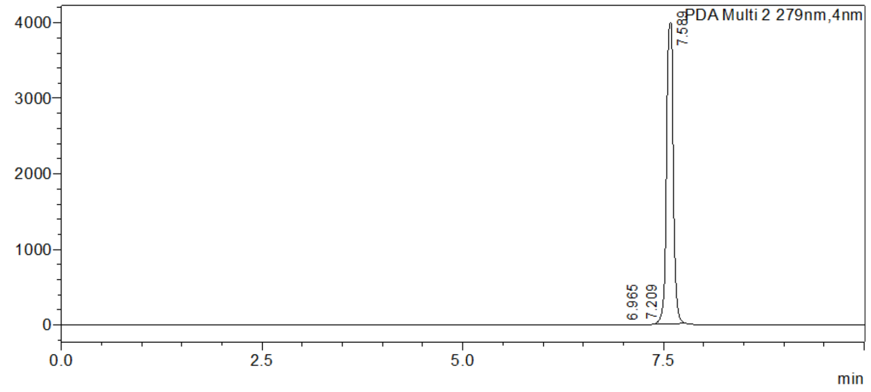

<Peak Table>

PDA Ch2 279nm

Peak#	Ret. Time	Peak Start	Peak End	Area	Height	Area%	Height%
1	6.965	6.944	6.997	1912	1065	0.008	0.027
2	7.209	7.147	7.275	19575	4479	0.083	0.112
3	7.589	7.371	7.776	23485063	3981756	99.909	99.861
Total				23506550	3987300	100.000	100.000

C:\LabSolutions\Data\Pyne\Wendy\ywc5-069.lcd

Compound **21d**

Analysis Report

<Sample Information>

Sample Name	: ywc5-072		
Sample ID	: ywc5-072		
Data Filename	: ywc5-072.lcd		
Method Filename	: Purity check 10min.lcm		
Batch Filename	:		
Vial #	: 1-1	Sample Type	: Unknown
Injection Volume	: 5 uL		
Date Acquired	: 29/10/2022 12:16:43 PM	Acquired by	: System Administrator
Date Processed	: 29/10/2022 12:26:44 PM	Processed by	: System Administrator

<Chromatogram>

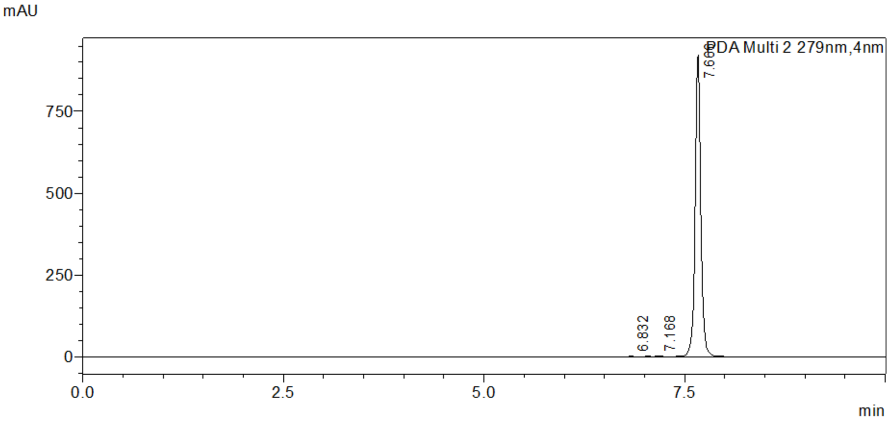

<Peak Table>

PDA Ch2 279nm

Peak#	Ret. Time	Peak Start	Peak End	Area	Height	Area%	Height%
1	6.832	6.773	6.997	1611	1498	0.035	0.162
2	7.168	6.997	7.317	14663	2181	0.323	0.236
3	7.666	7.381	8.021	4529855	920497	99.642	99.602
Total				4546129	924176	100.000	100.000

C:\LabSolutions\Data\Pyne\Wendy\ywc5-072.lcd

Compound **21e**

Analysis Report

<Sample Information>

Sample Name	: ywc5-073		
Sample ID	: ywc5-073		
Data Filename	: ywc5-073.lcd		
Method Filename	: Purity check 10min.lcm		
Batch Filename	:		
Vial #	: 1-1	Sample Type	: Unknown
Injection Volume	: 10 uL		
Date Acquired	: 29/10/2022 12:33:00 PM	Acquired by	: System Administrator
Date Processed	: 29/10/2022 12:43:01 PM	Processed by	: System Administrator

<Chromatogram>

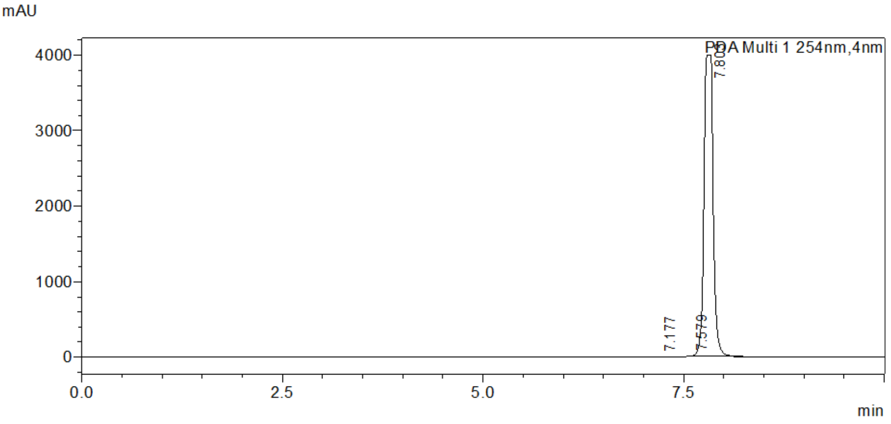

<Peak Table>

PDA Ch1 254nm

Peak#	Ret. Time	Peak Start	Peak End	Area	Height	Area%	Height%
1	7.177	7.136	7.221	9789	3046	0.031	0.076
2	7.579	7.488	7.616	21860	6442	0.069	0.161
3	7.803	7.616	8.267	31510429	3982781	99.900	99.762
Total				31542078	3992269	100.000	100.000

C:\LabSolutions\Data\Pyne\Wendy\ywc5-073.lcd

Compound **21f**

Analysis Report

<Sample Information>

Sample Name	: ywc5-074		
Sample ID	: ywc5-074		
Data Filename	: ywc5-074.lcd		
Method Filename	: Purity check 10min.lcm		
Batch Filename	:		
Vial #	: 1-1	Sample Type	: Unknown
Injection Volume	: 10 uL		
Date Acquired	: 29/10/2022 12:49:12 PM	Acquired by	: System Administrator
Date Processed	: 29/10/2022 12:59:13 PM	Processed by	: System Administrator

<Chromatogram>

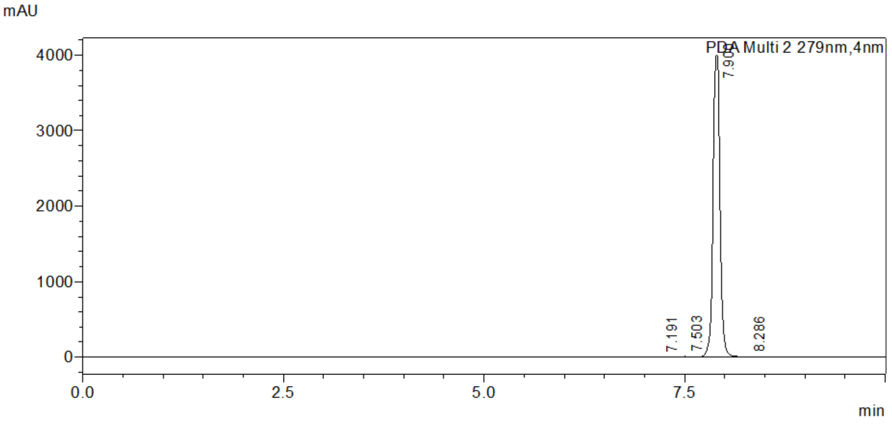

<Peak Table>

PDA Ch2 279nm

Peak#	Ret. Time	Peak Start	Peak End	Area	Height	Area%	Height%
1	7.191	7.093	7.264	10862	2557	0.045	0.064
2	7.503	7.435	7.680	22170	5619	0.093	0.140
3	7.900	7.680	8.181	23877440	3992990	99.847	99.759
4	8.286	8.213	8.373	3634	1489	0.015	0.037
Total				23914106	4002654	100.000	100.000

C:\LabSolutions\Data\Pyne\Wendy\ywc5-074.lcd

Compound **21g**

Analysis Report

<Sample Information>

Sample Name	: ywc5-079		
Sample ID	: ywc5-079		
Data Filename	: ywc5-079.lcd		
Method Filename	: Purity check 10min.lcm		
Batch Filename	:		
Vial #	: 1-1	Sample Type	: Unknown
Injection Volume	: 10 uL		
Date Acquired	: 29/10/2022 1:21:08 PM	Acquired by	: System Administrator
Date Processed	: 29/10/2022 1:31:09 PM	Processed by	: System Administrator

<Chromatogram>

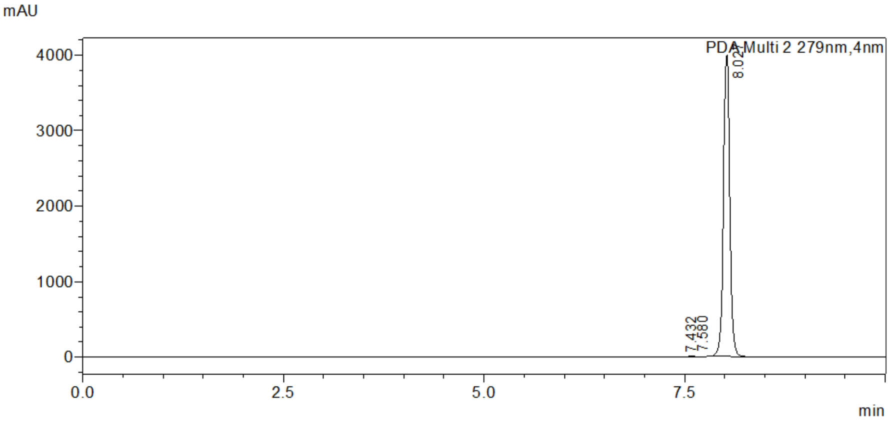

<Peak Table>

PDA Ch2 279nm

Peak#	Ret. Time	Peak Start	Peak End	Area	Height	Area%	Height%
1	7.432	7.392	7.477	3856	1237	0.018	0.031
2	7.580	7.509	7.691	51738	9044	0.235	0.226
3	8.027	7.808	8.309	21967301	3986870	99.748	99.743
Total				22022895	3997151	100.000	100.000

C:\LabSolutions\Data\Pyne\Wendy\ywc5-079.lcd

Compound **21h**

Analysis Report

<Sample Information>

Sample Name	: ywc5-078		
Sample ID	: ywc5-078		
Data Filename	: ywc5-078.lcd		
Method Filename	: Purity check 10min.lcm		
Batch Filename	:		
Vial #	: 1-1	Sample Type	: Unknown
Injection Volume	: 5 uL		
Date Acquired	: 29/10/2022 1:05:06 PM	Acquired by	: System Administrator
Date Processed	: 29/10/2022 1:15:07 PM	Processed by	: System Administrator

<Chromatogram>

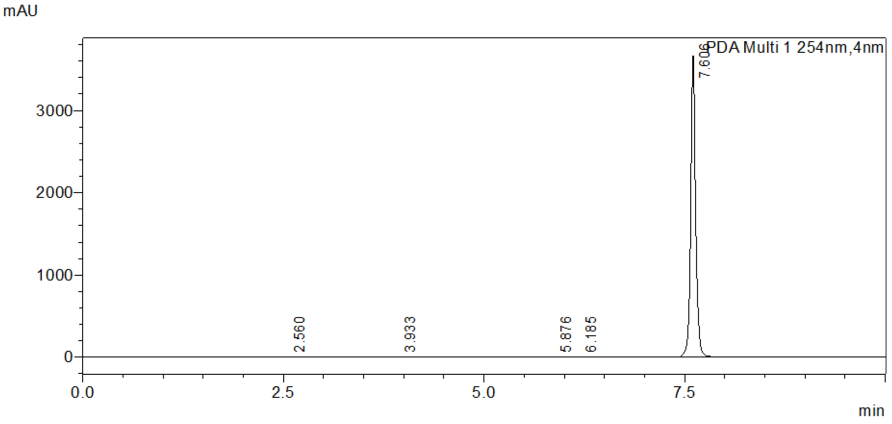

<Peak Table>

PDA Ch1 254nm

Peak#	Ret. Time	Peak Start	Peak End	Area	Height	Area%	Height%
1	2.560	2.453	3.456	52209	897	0.336	0.024
2	3.933	3.840	4.064	304	40	0.002	0.001
3	5.876	5.803	5.941	444	99	0.003	0.003
4	6.185	6.101	6.197	92	28	0.001	0.001
5	7.606	7.413	7.861	15501988	3668536	99.659	99.971
Total				15555037	3669601	100.000	100.000

C:\LabSolutions\Data\Pyne\Wendy\ywc5-078.lcd
